# A novel method of consensus pan-chromosome assembly and large-scale comparative analysis reveal the highly flexible pan-genome of *Acinetobacter baumannii*

**DOI:** 10.1186/s13059-015-0701-6

**Published:** 2015-07-21

**Authors:** Agnes P. Chan, Granger Sutton, Jessica DePew, Radha Krishnakumar, Yongwook Choi, Xiao-Zhe Huang, Erin Beck, Derek M. Harkins, Maria Kim, Emil P. Lesho, Mikeljon P. Nikolich, Derrick E. Fouts

**Affiliations:** J. Craig Venter Institute (JCVI), Rockville, MD USA; Department of Emerging Bacterial Infections, Bacterial Diseases Branch, Walter Reed Army Institute of Research, Silver Spring, MD USA; Multidrug-resistant organism Repository and Surveillance Network, Bacterial Diseases Branch, Walter Reed Army Institute of Research, Silver Spring, MD USA

## Abstract

**Background:**

Infections by pan-drug resistant *Acinetobacter baumannii* plague military and civilian healthcare systems. Previous *A. baumannii* pan-genomic studies used modest sample sizes of low diversity and comparisons to a single reference genome, limiting our understanding of gene order and content. A consensus representation of multiple genomes will provide a better framework for comparison. A large-scale comparative study will identify genomic determinants associated with their diversity and adaptation as a successful pathogen.

**Results:**

We determine draft-level genomic sequence of 50 diverse military isolates and conduct the largest bacterial pan-genome analysis of 249 genomes. The pan-genome of *A. baumannii* is open when the input genomes are normalized for diversity with 1867 core proteins and a paralog-collapsed pan-genome size of 11,694 proteins. We developed a novel graph-based algorithm and use it to assemble the first consensus pan-chromosome, identifying both the order and orientation of core genes and flexible genomic regions. Comparative genome analyses demonstrate the existence of novel resistance islands and isolates with increased numbers of resistance island insertions over time, from single insertions in the 1950s to triple insertions in 2011. Gene clusters responsible for carbon utilization, siderophore production, and pilus assembly demonstrate frequent gain or loss among isolates.

**Conclusions:**

The highly variable and dynamic nature of the *A. baumannii* genome may be the result of its success in rapidly adapting to both abiotic and biotic environments through the gain and loss of gene clusters controlling fitness. Importantly, some archaic adaptation mechanisms appear to have reemerged among recent isolates.

**Electronic supplementary material:**

The online version of this article (doi:10.1186/s13059-015-0701-6) contains supplementary material, which is available to authorized users.

## Background

*Acinetobacter baumannii* is a Gram-negative, non-fermenting coccobacillus that can be found in soil and water, but in recent decades has been recognized as an emerging multidrug-resistant (MDR) nosocomial pathogen causing pneumonia, bacteremia, meningitis, and skin/soft-tissue infection associated with trauma [1–5]. The Centers for Disease Control and Prevention (CDC) estimates that each year in the US there are 12,000 healthcare-associated *Acinetobacter* infections, 63 % of which are MDR [6]. In 2010 an expert panel deemed MDR organisms one of the top five infectious threats to the US Military [7]. Infections with *A. baumannii* resistant to nearly every available antibiotic complicate the care of many patients [8, 9]. Surveillance for asymptomatic colonization among injured service members reveals *A. baumannii* to be one of the common Gram-negative MDR pathogens isolated along with *Acinetobacter calcoaceticus* and *Klebsiella pneumoniae* [10].

The genetic factors that contribute to the success of *A. baumannii* as a pathogen, such as biofilm formation, ability to compete for and sequester iron in nutrient-deprived environments, and resistance to multiple broad-spectrum antibiotics, have been areas of intense study. In a recently published study of 97 clinical isolates collected from military treatment facilities, 80 % were found to be MDR with markers known to confer resistance to β-lactams, aminoglycosides, macrolides, tetracycline, phenicol, quaternary amines, streptothricin, sulfonamides, and diaminopyrimidine [11]. Drug resistance is manifested by a number of well-characterized mechanisms, including inactivation of drugs (e.g., β-lactamases, cephalosporinases, carbapenemases), prevention of drug entry through outer membrane alterations, removal of the drugs via efflux pumps, and mutations in drug targets [12–18]. In addition, *A. baumannii* has the capacity to up-regulate expression of resistance mechanisms [19–24] and acquire new determinants on genomic regions called resistance islands (RIs) [25], especially in environments such as hospitals where broad spectrum antibiotics are in use [26].

Previous *A. baumannii* comparative genomics studies used modest sample sizes to study representative strains causing infections worldwide. Di Nocera et al. [27] compared seven *A. baumannii* strains, including three of the most frequent strains responsible for epidemics in Mediterranean hospitals. Sahl et al. [28] compared 23 isolates, including three they sequenced, for the presence/absence of invasion- and colonization-specific genes and conducted a pan-genome analysis of six complete genomes. Whole genome phylogenetic analysis of 136 *Acinetobacter* genomes was used to shed light on the expansion of the genus occurring through the gain and loss of genes and conservation of pathogenesis associated genes in the *Acinetobacter calcoaceticus-baumannii* complex [29]. Recently, pan-genome analysis on 34 [30] and 35 [31] *A. baumannii* isolates was conducted.

Since the use of a single reference genome would limit our understanding of gene order and content to a single isolate, comparisons with all available related genomes would be preferable. Thus, a consensus representation of multiple genomes would provide a better framework for comparison than a single reference genome. Methods for constructing the consensus of bacterial strains do not yet exist as far as we know; however, methods do exist to reconstruct contiguous regions of ancestral eukaryotic genomes based on evolutionary breakpoints or rearrangements [32–34]. These methods would fail to assemble a consensus prokaryotic genome by not capturing variable regions acquired via horizontal gene transfer events that were nonexistent in the ancestor. In addition, methods that rely on rearrangements will not work with draft genomes. These limitations necessitated the development of a new program, *gene_order.pl*, which computes the consensus pan-genome from the output generated by our pan-genome ortholog clustering tool, PanOCT [35].

Here we compare genomic features from the largest number of *A. baumannii* isolates of clinical and military relevance using a pan-genome analysis of 249 publicly available *A. baumannii* isolates, of which 50 were sequenced at the J. Craig Venter Institute (JCVI) for this study. The 249 isolates were collected over several decades and also represented a global collection obtained from hospitals in the US and around the world. First, using *gene_order.pl* as described above, we assembled the first consensus “pan-chromosome” independent of any pre-assigned genome reference and identified both invariant (core) and variable (flexible) regions within the chromosome, which are key components that define a bacterial strain. Second, we utilized a comparative genomics approach on 249 genomes to analyze the diversity of RIs and virulence factors of *A. baumannii*. Our results revealed that decades-old isolates already encoded a vast collection of genetic determinants and mechanisms to confer antibiotic resistance and survival adaptations. We demonstrated the existence of novel RIs and isolates with increased number of RI insertions over time. Clusters of genes for carbon source utilization, siderophore production, pilus assembly and resistance mechanisms were highly variable, and some of these may have reemerged, sometimes in different genomic locations, among modern isolates. These analyses will provide insight into the evolution of *A. baumannii* as a nosocomial pathogen and directly aid the future efforts for large-scale epidemiological studies of this continuously evolving MDR organism.

## Results

### Genome sequencing of new *A. baumannii* isolates from the military healthcare system

A total of 50 isolates identified as *A. baumannii* from the US military healthcare system were chosen for whole genome shotgun sequencing based on novel clustering by pulsed-field gel electrophoresis (PFGE; Additional file 1), increased prevalence in the military healthcare system, or pan-drug resistance profiles (e.g., Multidrug-resistant Organism and Surveillance Network (MRSN) isolates; Table 1; Additional file 2). These strains were isolated between 2003 and 2011 and comprised 23 different known sequence types (STs) from multilocus sequence typing (MLST) with one potentially novel predicted ST. Seventeen of the isolates were sequenced with a genome finishing status of “improved high-quality draft” (IHQD) (Table 1; Additional file 2), which included manual finishing through sequence gap closure, PCR to link physical ends, or automated gap closure.The remaining isolates were sequenced to a “high-quality draft” (HQD) status. On average, the genomes assembled into 65 contigs (range 3 to 197), 4,023,048 bp in length (range 3,740,684 to 4,454,613 bp) with 3885 predicted protein-coding sequences (range 3505 to 4406). Antibiotic susceptibility profiles and predicted resistance mechanisms are presented in Additional file 3. For one isolate, Naval-83, an amino acid substitution previously not observed in *Acinetobacter* (Glu88Lys) was identified in *parC*, which was recently shown to confer resistance to levofloxacin in *Haemophilus influenza* [36].

**Table 1:** Select genomic features and metadata of *A. baumannii* genomes sequenced in this study

Number	Strain	Accession	G+C %	Finishing status^†^	Number of contigs	Number of proteins	Length (bp)	MLST ST	MLST allelic profile^§^	Origin/site	Country	City	Year	Reference
1)	OIFC137	AFDK00000000	36.9	IHQD	4	3871	4,081,420	3	3-3-2-2-3-1-3	Catheter tip	USA	Washington, DC	2003	
2)	OIFC032	AFCZ00000000	40.8	IHQD	4	3718	3,893,886	32	1-1-2-2-3-4-4	Wound	Germany	Landstuhl	2003	[8]
3)	OIFC109	ALAL00000000	38.4	IHQD	13	3945	4,107,121	3	3-3-2-2-3-1-3	Right residual limb wound	USA	Washington, DC	2003	[8]
4)	OIFC143	AFDL00000000	37.3	IHQD	8	4265	4,441,327	25	3-3-2-4-7-2-4	Thigh wound	USA	Washington, DC	2003	
5)	OIFC189	AFDM00000000	44.1	IHQD	10	3849	4,043,115	2	2-2-2-2-2-2-2	Wound	USA	Bethesda, MD	2003	[88]
6)	Canada BC-5	AFDN00000000	38.0	IHQD	3	3787	3,998,016	1	1-1-1-1-5-1-1	Clinical isolate	Canada*	NA	2007	
7)	Naval-17	AFDO00000000	42.9	IHQD	21	3848	4,009,964	2	2-2-2-2-2-2-2	Wound	USA	Bethesda, MD	2006	[87]
8)	Naval-18	AFDA00000000	37.6	IHQD	11	4406	4,454,613	25	3-3-2-4-7-2-4	Wound	USA	Bethesda, MD	2006	[87]
9)	Naval-81	AFDB00000000	36.5	IHQD	5	3981	4,080,872	3	3-3-2-2-3-1-3	Blood	USA	Bethesda, MD	2006	
10)	IS-123	ALII00000000	38.7	IHQD	20	4013	4,063,081	3	3-3-2-2-3-1-3	Wound	Iraq	Baghdad	2009	
11)	OIFC074	AMDE00000000	40.5	HQD	66	3815	3,935,888	19	1-2-1-1-5-1-1	Clinical isolate	Germany	Landstuhl	2003	
12)	OIFC098	AMDF00000000	39.5	HQD	72	3659	3,812,112	10	1-3-2-1-4-4-4	Clinical isolate	Germany	Landstuhl	2003	
13)	OIFC180	AMDQ00000000	40.1	HQD	141	3942	3,986,823	2	2-2-2-2-2-2-2	Clinical isolate	USA	NA	2003	
14)	Naval-13	AMDR00000000	40.6	HQD	64	3948	4,107,737	3	3-3-2-2-3-1-3	Wound	USA	Bethesda, MD	2006	[87]
15)	IS-235	AMEI00000000	41.0	HQD	76	3981	4,060,387	1	1-1-1-1-5-1-1	Blood	Iraq	Baghdad	2008	[88]
16)	IS-251	AMEJ00000000	39.6	HQD	72	3908	4,007,286	1	1-1-1-1-5-1-1	Respiratory tract	Iraq	Baghdad	2008	[88]
17)	OIFC0162	AMFH00000000	39.4	HQD	55	3856	4,078,399	412	1-52-2-2-67-4-5	Trachea	USA	Washington, DC	2003	[8]
18)	Naval-72	AMFI00000000	41.2	HQD	52	3607	3,840,453	405	5-3-16-4-29-1-60	Wound	USA	Bethesda, MD	2006	[87]
19)	Naval-83	AMFK00000000	39.7	HQD	103	4000	4,106,603	20	3-1-1-1-5-1-1	Wound	USA	Bethesda, MD	2006	[87]
20)	OIFC110	AMFL00000000	40.3	HQD	53	3818	3,981,666	515	56-3-2-2-9-4-14	Clinical isolate	Germany	Landstuhl	2003	
21)	IS-143	AMGE00000000	41.1	HQD	93	3883	4,020,019	414	2-2-2-2-2-37-2	Wound	Iraq	Baghdad	2008	
22)	IS-116	AMGF00000000	40.0	HQD	40	3779	3,952,511	136	3-2-19-25-5-2-5	Wound	Iraq	Baghdad	2008	
23)	WC-692	AMGG00000000	39.7	HQD	79	4004	4,183,446	513	56-3-55-2-9-4-14	Intact skin surface	Iraq	NA	2008	
24)	IS-58	AMGH00000000	40.9	HQD	61	3944	4,063,888	1	1-1-1-1-5-1-1	Respiratory tract	Iraq	Baghdad	2008	[88]
25)	WC-487	AMZR00000000	39.5	HQD	121	3994	4,115,076	410	20-26-26-14-26-16-23	Skin	USA	Bethesda, MD	2008	
26)	WC-348	AMZT00000000	39.2	HQD	61	3897	4,108,488	412	1-52-2-2-67-4-5	Intact skin surface	Iraq	NA	2008	
27)	Naval-113	AMZU01000000	49.7	HQD	130	4002	4,095,626	2	2-2-2-2-2-2-2	Wound	USA	Bethesda, MD	2006	[87]
28)	Naval-82	AMSW00000000	38.2	HQD	197	3969	3,908,929	428	3-1-2-3-6-1-16	Blood	USA	Bethesda, MD	2006	[87]
29)	Naval-2	AMSX00000000	39.5	HQD	114	4074	4,126,550	2	2-2-2-2-2-2-2	Blood	USA	Bethesda, MD	2006	[87]
30)	Naval-21	AMSY00000000	40.0	HQD	75	3829	3,923,796	19	1-2-1-1-5-1-1	Wound	USA	Washington, DC	2006	[87]
31)	Canada BC1	AMSZ00000000	39.7	HQD	66	3825	3,936,404	1	1-1-1-1-5-1-1	Nosocomial infection	Canada	NA	2007	
32)	WC-A-694	AMTA00000000	39.7	HQD	82	3830	4,008,103	3	3-3-2-2-3-1-3	Clinical isolate	USA	Washington, DC	2008	
33)	OIFC035	AMTB00000000	43.0	HQD	44	3741	3,972,611	403	3-2-6-1-3-4-59	Groin wound	USA	Washington, DC	2003	
34)	Naval-57	AMFP00000000	40.7	HQD	138	3838	3,953,596	155	3-2-2-2-44-4-4	Wound	USA	Bethesda, MD	2006	
35)	OIFC087	AMFS00000000	39.1	HQD	96	3922	4,004,682	32	1-1-2-2-3-4-4	Perineum	USA	Washington, DC	2003	
36)	OIFC099	AMFT00000000	40.1	HQD	75	3748	3,918,177	32	1-1-2-2-3-4-4	Environmental sample	USA	Washington, DC	2003	[8]
37)	WC-A-92	AMFU00000000	38.1	HQD	151	3802	3,838,812	431	1-4-2-1-70-1-2	Clinical isolate	USA	Washington, DC	2007	
38)	OIFC065	AMFV00000000	39.5	HQD	54	3893	4,029,646	136	3-2-19-25-5-2-5	Left leg	USA	Washington, DC	2003	
39)	OIFC047	AMFW00000000	39.2	HQD	39	3505	3,740,684	Novel	1-75-2-2-67-1-2	Perineum	USA	Washington, DC	2003	
40)	OIFC338	AMFX00000000	40.1	HQD	108	4081	4,155,681	2	2-2-2-2-2-2-2	Clinical isolate	USA	Washington, DC	2003	
41)	OIFC111	AMFY00000000	40.8	HQD	44	3732	3,988,061	49	3-3-6-2-3-1-5	Perineum	USA	Washington, DC	2003	
42)	Naval-78	AMFZ00000000	39.9	HQD	112	3950	4,053,379	2	2-2-2-2-2-2-2	Wound	USA	Bethesda, MD	2006	[87]
43)	AA-014	AMGA00000000	39.4	HQD	61	3618	3,857,932	158	41-42-13-1-5-4-14	Wound	Iraq	Al Anbar	2008	
44)	MRSN 3405	JPIA00000000	38.3	IHQD	64	3958	4,082,715	94	1-2-2-1-5-1-1	Wound	USA	Washington, DC	2011	[15]
45)	MRSN 3527	JPHZ00000000	38.7	IHQD	46	4101	4,206,186	81	1-1-1-1-5-1-2	Wound	USA	Washington, DC	2011	[15]
46)	MRSN 3942	JPHY00000000	38.4	IHQD	69	3849	3.975,719	94	1-2-2-1-5-1-1	Wound	USA	Washington, DC	2011	[15]
47)	MRSN 4106	JPHX00000000	38.6	IHQD	62	3824	3,952,684	94	1-2-2-1-5-1-1	Wound	USA	Washington, DC	2011	[15]
48)	MRSN 58	JPHW00000000	39.8	IHQD	40	3866	3,974,176	1	1-1-1-1-5-1-1	Wound	USA	Washington, DC	2010	[20]
49)	MRSN 7339^‡^	JPHV00000000	39.3	IHQD	34	3787	3,955,466	1	1-1-1-1-5-1-1	Wound	USA	Washington, DC	2004	
50)	MRSN 7341^‡^	JPIB00000000	39.4	IHQD	52	3766	3,911,280	2	2-2-2-2-2-2-2	Respiratory	USA	Washington, DC	2004	

^†^Improved high-quality draft (IHQD); high-quality draft (HQD)

^§^ *cpn60*:*fusA*:*gltA*:*pyrG*:*recA*:*rplB*:*rpoB*

*Sample isolated from a soldier evacuated via Landstuhl Regional Medical Center

^‡^Isolated from the same individual

*MLST* multilocus sequence typing, *NA* not available, *NI* not identified

### Pan-genome

Despite the intensive effort to characterize *A. baumannii* and the sizable number of whole genome comparisons published in the past decade [26, 29, 37–40], the size of the pan-genome remains unknown. We set out to determine the pan-genome of *A. baumannii*. Using PanOCT [35], a total of 22,281 orthologous protein clusters were identified from a collection of all *A. baumannii* genomes publicly available at the time of the analysis, which included 50 sequenced in this study plus 199 genomes obtained from GenBank, totaling 249 genomes (Additional files 4 and 5).

**Fig. 1:**
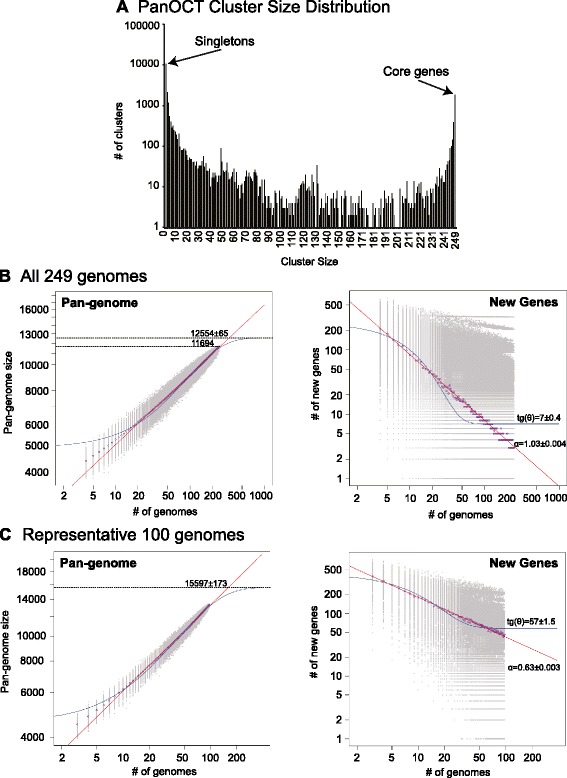
Analysis of the *A. baumannii* pan-genome. The distribution of protein cluster sizes generated from the comparison of 249 *A. baumannii* genomes using PanOCT [35] indicates the number of singleton and core genes (**a**). The pan-genome size (*left panel*) and the number of novel genes discovered with the addition of each new genome (*right panel*) were estimated for all 249 genomes (**b**) and a set of 100 representative genomes identified by hierarchical clustering of all 249 genomes (**c**) using a pan-genome model based on the original Tettelin et al. model [42]. *Purple circles* are the median of each distribution (*gray circles*). Power law (*red lines*) and exponential (*blue lines*) regressions were plotted to determine α (open/closed status) and tg(θ), the average extrapolated number of strain-specific/novel genes, respectively [41]

PanOCT only includes non-paralogs in clusters and uses conserved gene neighborhood to separate duplicated genes. This means that insertion sequence (IS) elements that are in novel contexts will often form singleton clusters even though they are identical in sequence to other IS elements within or between genomes analyzed. When the “core” pan-genome is defined to be all 249 genomes analyzed (100 %), there were 1867 core/universal protein clusters and 10,602 singleton clusters (i.e., clusters with a single member from a single genome) (Fig. 1a). If the core pan-genome were instead defined as clusters having protein members from 95 % or 75 % of the genomes analyzed, the core pan-genome would be 2833 and 3126, respectively.

For the analysis of pan-genome size, we followed the convention of merging clusters of paralogous proteins, which greatly reduced the number of clusters from 22,281 to 11,694. To predict the theoretical maximum pan-genome size (i.e., the total number of genes, including core/universal, novel/unique/strain-specific and periphery/dispensable genes) a pan-genome model was implemented using medians and an exponential decay function [41] (Fig. 1b). The maximum pan-genome size was estimated to be 12,554 ± 65 genes. To determine whether the *A. baumannii* pan-genome is open or closed, the number of new genes identified (i.e., unique or strain-specific genes) for each genome added was determined and fit to a power law function (n = κN^-α^) as described previously [42] (Fig. 1b). Conceptually, a pan-genome is closed when sequencing the genomes of additional isolates fails to expand the pan-genome (i.e., the entire gene repertoire has been discovered) [43]. The exponent (α) indicates whether the pan-genome is open (α ≤ 1) or closed (α > 1) [41]. Using this equation, the pan-genome of *A. baumannii* appears to be barely closed (α = 1.03 ± 0.004; Fig. 1b). For each genome added, the number of new genes was extrapolated by calculating tg(θ) (from an exponential decay function), which was determined to be 7 ± 0.4 (Fig. 1b).

Since a large number of the *A. baumannii* isolates included in this study were of MLST ST 2 (Additional file 4), it is possible the results of the pan-genome state (i.e., open versus closed) were biased toward this dominant ST. Using a phylogenetic tree computed from the BLAST score ratio (BSR) distance matrix generated by PanOCT (Additional file 6), 100 genomes were selected by hierarchical clustering (gold label, Additional file 6). This set of 100 genomes, which represents an even distribution of *A. baumannii* genomic diversity, had a theoretical maximum pan-genome size larger than the combined 249 dataset (by ~3000 genes), with 15,597 ± 173 genes and 57 ± 1.5 new genes discovered for each genome added (Fig. 1c). The pan-genome of the diverse 100 genomes was also open (α = 0.63 ± 0.003; Fig. 1c). In contrast, the theoretical maximum pan-genome size obtained from just the ST 2 genomes decreased to 7980 ± 68 genes and the ST 2 pan-genome was closed (α = 1.08 ± 0.002; Additional file 7).

### Flexible genomic islands

Genomic variations among bacterial strains are often found to be mobile elements (e.g., prophage, plasmids, integrated elements), or variable or “flexible” regions that encode genes involved in cell surface structures (e.g., O-antigen, capsular polysaccharides, teichoic acid, S-layer, flagella, pili, and porins) as well as genes for nutrient utilization. All such highly variable regions have been referred to as flexible genomic islands (fGIs) [44–50].

**Fig. 2:**
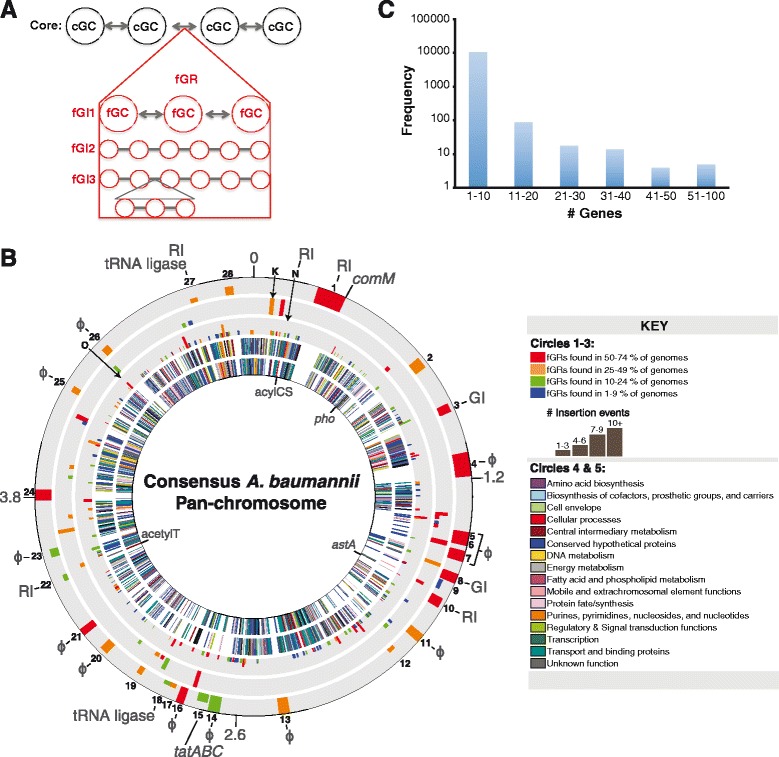
Pan-chromosome and fGIs. The core gene cluster (cGC), flexible gene cluster (fGC) and flexible genomic region (fGR) locations of the *A. baumannii* pan-genome (**a**) were computed from PanOCT output and are illustrated as a circle where each concentric circle is numbered from the outermost to the innermost circle (**b**). fGR locations are depicted in circles 1 (>20,000 bp), 2 (10,001–20,000 bp), and 3 (1000–10,000 bp) on a core backbone of genes in circles 4 (positive strand) and 5 (negative strand). Refer to the key for details on color representations, circle number and bar height. Key fGRs are noted by black numbering and letters *K* (K-antigen), *N* (Novel), and *O* (O-antigen) as in Table 2. Gray numbers indicate positions on the pan-chromosome in megabase pairs. Predicted functions are noted in gray words and the following abbreviations: *RI* resistance island, *GI* genomic island, *ɸ* phage. Genes associated with RI insertions are labeled in *gray* and indicated by a *black line*. The frequency of individual fGIs within six size class bins was also determined (**c**)

As a prerequisite to identifying fGIs in the pan-genome, a consensus core backbone and fGI assemblies of the pan-genome were computed using *gene_order.pl* (Additional file 8). This algorithm uses output generated by PanOCT to link core gene clusters (cGCs) based on the consensus of the layout of the cGCs in individual genomes (Fig. 2a). The cGCs were defined as containing genes from 75 % or more of the 249 genomes, resulting in a consensus core “pan-chromosome” of *A. baumannii* composed of 3126 genes whose coding regions totaled 2,988,228 bp. When the maximum sizes of all fGIs were inserted into the core backbone, the maximum size of the pan-chromosome increased to 5,070,600 bp, which is 1,047,552 bp (~20 %) larger than the average genome size of 4,023,048 bp. The constructed pan-chromosome had a circular topology (rings 4 and 5 of Fig. 2b), indicating that cGCs were linked together forming a circle as expected, even though the majority of genome assemblies comprising the pan-genome are in draft status or possibly incomplete. In addition to the chromosome, seven additional circular “assemblies” were determined that encode between 2 and 120 genes. Five of the circular assemblies were identified as sharing homology to known *A. baumannii* plasmids pABTJ2 [51], pAB2 [52], pRAY [53], and p4ABAYE [54]. Two of these circular assemblies were of bacteriophage and IS element origin.

In addition to generating a consensus core backbone, *gene_order.pl* identified the location of flexible genomic regions (fGRs), which are variable regions between cGCs of the pan-chromosome (Fig. 2a). These fGRs are composed of a collection of fGIs (Additional file 9). A given fGI is an instance of genomic sequence variation observed at the fGR. Each fGI in turn is made up of individual linear assemblies of flexible genomic clusters (Fig. 2a). In order to avoid and filter out spurious fGIs due to random IS elements or bad gene calls, we required any fGIs carrying less than three genes in length to be present in at least 10 % of the genomes analyzed. To be included within an fGR, we required fGIs of three or more genes in length to be present in at least three genomes. The fGRs are illustrated on the outer rings 1–3 of Fig. 2b. The majority of fGIs contained between one and ten genes (Fig. 2c), which are composed of IS elements, gene duplications, the O-antigen biosynthesis cluster (labeled “O” in Fig. 2b) and other small variable biosynthetic gene clusters. There were 89 fGIs encoding 11–20 genes (ring 2, Fig. 2b) and 41 fGIs encoding 21+ genes (ring 1, Fig. 2b). The largest fGI encoded 97 genes, was 79,689 bp in length, similar to phage 3 in ACICU [37], and highly prevalent (present in 151 genomes).

### fGIs in the largest fGRs

The largest fGI assemblies within the 20+ kb fGR size class were analyzed for functionality, their potential role in virulence, survival, drug resistance, and evidence of lateral transfer. While many fGRs were targets for insertion of fGIs that encode bacteriophage components (fGRs 4–7, 11, 13, 14, 16, 20, 21, 23, 25, 26), we identified metabolic pathways, drug resistance genes, and potential virulence factors as well as unusual duplications of typical core genes that were inserted within the largest fGRs. Some of these fGRs contained fGIs that were reported previously, such as the putative “alien islands”, a.k.a. “pAs”, reported in the MDR *A. baumannii* strain ACICU [38] (Table 2).

**Table 2:** Analysis of select fGIs from the *A. baumannii* pan-chromosome

fGR					Functional categories										
Region number^+^	End5	End3	Span (bp)	Number of fGIs	RI	GI	Phage	Metabolic	House-keeping	Extracellular polysaccharide	Description of largest fGI-encoded functions^£^	pA_ICU_^¶^	Flanking^†^ core ACICU loci	fGR id^‡^	Largest fGI assemblies^§^
K	68497	82812	14316	17						X	K-antigen	1	00074/00087	CL_INS_4	105
N	152392	155346	2955	3	X						Novel; acetyltransferase, fragment of composite IS26 transposon	-	00139 (acylCS)/00147	CL_INS_12	791*
1	243163	350187	107025	38	X						*comM*, aminoglycoside/hydroxyurea antibiotic resistance kinase, streptomycin 3″-kinase, transporter, major facilitator family protein	3	00219/00242	CL_INS_20	58*
2	702940	747393	44454	7		X					Copper/heavy metal resistance	-	00567/00568	CL_INS_49	16
3	906910	938373	31464	9		X					Phage 1 in ACICU, but no core phage genes	6	00684/00702	CL_INS_65	64
4	1103071	1189920	86850	62			X				Toxin/anti-toxin system, large terminase, methylase	-	00861/00869	CL_INS_74	9
5	1401709	1425417	23709	43			X				phage protein F-like	-	01048/01056	CL_INS_93	29
6	1425814	1449657	23844	31			X				Arc-like protein, lysozyme			CL_INS_94	76
7	1472644	1510578	37935	16			X				Major capsid, prohead protease, portal, large and small terminase, head-tail adaptor, tail protein, lysozyme, antitermination protein Q, integrase			CL_INS_97	14
8	1561693	1595358	33666	11		X					Zeta toxin, phage/plasmid-like protein, recombinase	-	01106/01110	CL_INS_101	52
9	1603966	1639890	35925	2				X			*bla*, *hlyD*, phenylpropanoid catabolism, porin, acetaldehyde dehydrogenase; * ompA*-like, *yadA*-like	-	01115/01116	CL_INS_104	38, 260*
10	1666240	1704336	38097	7				X			Aldehyde dehydrogenase, vanillin dehydrogenase, porins, transporters	-	01136 (*astA*)/01153	CL_INS_107	28
11	1818532	1852125	33594	12			X				Lysis protein, tail, tail assembly, tape measure	-	01222/01224	CL_INS_120	50
12	1928539	1956234	27696	2				X			*bla*, *hlyD*, phenylpropanoid catabolism, porin, acetaldehyde dehydrogenase	-	01256/01257	CL_INS_128	38
13	2402650	2440626	37977	32			X				Major capsid, prohead protease, portal, large and small terminase, head-tail adaptor, tail protein, lysozyme, antitermination protein Q, integrase	-	01626/01627	CL_INS_164	14
14	2664622	2705871	41250	11			X				Inovirus-like; zonula occludens toxin, coat protein B, replication protein	-	01813/01815	CL_INS_180	110
15	2716186	2754267	38082	6		X			X		*tatABC*, TonB receptors, ABC transporters	-	01824/01827	CL_INS_182	33
16	2802271	2828589	26319	15			X				Phage-associated protein, phage protein F-like, site-specific recombinase	-	01849/01864	CL_INS_187	40
17	2850997	2872608	21612	2				X			Oxidoreductase, aldehyde dehydrogenase	-	01886/01887	CL_INS_190	62
18	2874184	2899845	25662	2				X	X		tRNA ligase, aldehyde dehydrogenases, Homoprotocatechuate/hydroxyphenylacetate degradation, transporter	-	01887/01888	CL_INS_191	37
19	2990911	3011439	20529	4				X			Medium chain fatty acid ligase, transporter, oxidoreductase	-	01936/01949	CL_INS_199	121
20	3143872	3176853	32982	7			X				Mu-like; Gam-like protein, terminase, methylase, Mu protein F-like, Mu-like major head, tail sheath-like, Mu Gp45, baseplate J-like	-	02064/02066	CL_INS_211	15
21	3250042	3277383	27342	12			X				lysozyme, baseplate, phage protein F-like, phage-associated protein	-	02139/02236	CL_INS_216	25
22	3465244	3487854	22611	3		X					Novel 7.8 kb region; salicylate monooxygenase	-	02398/02399 (acetylT)	CL_INS_237	338*
23	3569029	3597828	28800	6			X				Phage	-	02457/02470	CL_INS_246	19
24	3790117	3828819	38703	12				X			Rubredoxin, MFS transporter, prevent host death, aldehyde dehydrogenase, methylmalonate-semialdehyde dehydrogenase	29	02595/02624	CL_INS_259	35
25	4223758	4247211	23454	6			X				Integrase, CII, large and small terminase, portal, prohead protease, major capsid, head-tail connector, head-tail joining, tail	-	03014/03015	CL_INS_287	59
O	4387696	4395870	8175	5						X	O-antigen	-	03146/03149	CL_INS_297	168
26	4420246	4450038	29793	6			X				P2-like; integrase, tape measure, tail proteins, baseplate J-like, lysozyme, baseplate assembly, large and small terminase, major capsid, capsid scaffolding protein, portal	-	03157/03161	CL_INS_299	17
27	4818799	4844895	26097	2	X				X		Novel; *bla*, Tn7, sulfur transport, *fur*, tRNA ligase	-	03502/03503	CL_INS_334	39
28	4960555	4989495	28941	5		X					*ompA*-like protein, Tnp	-	03594/03595	CL_INS_346	148

^+^Cross reference with Fig. 4B

^£^Only functions from select fGIs are listed. Other elements and functions may be encoded within selected fGRs

^†^Not all fGIs are present in ACICU

^¶^Largest fGI in fGR similar in composition to the “alien islands” reported by Iacono et al. [38]

^‡^Cross reference Additional files 5 and 21

^§^Cross reference Additional files 18 and 21

*Not largest fGI in region

### Virulence genes in fGRs

In *A. baumannii*, the outer membrane protein OmpA is associated with biofilm formation [55], resistance to antibiotics [56] and increased cytotoxicity of outer membrane vesicles in cell cultures [57], where a number of OmpA and OmpA-like proteins were present in outer membrane vesicle preparations. Although several OmpA domain proteins were found in the core pan-genome, we also located SmpA/OmlA family proteins and multiple OmpA domains in some fGIs within fGR 9 (Fig. 2b, Table 2). In addition to OmpA-like adhesins, we identified a YadA-like domain protein in fGR 9, which in *Yersinia* is known to be a major virulence factor functioning in adhesion and complement evasion [58].

fGIs were also identified that encode proteins with putative roles in iron regulation. For example, a homolog of the ferric uptake regulator protein (Fur), which is required for iron homoeostasis and defense against reactive oxygen species [59], was identified in an fGI within fGR 27 (Fig. 2b, Table 2). There is an additional copy of *fur* found in the core pan-genome, which was previously identified as conserved between *Acinetobacter baylyi* and *A. baumannii* strains [54]. Additionally, putative TonB-dependent transporters/receptors were identified in fGR 15 (Fig. 2b, Table 2). TonB-dependent transporters/receptors are outer membrane proteins that bind and transport nutrients for energy metabolism, iron-chelating siderophores, and other metal-containing complexes [60], have been previously shown to be involved in bacterial virulence in some *A. baumannii* strains and were horizontally transferred [61], as is consistent with being within a fGI. At least four other TonB-like transporter genes were identified within smaller fGIs.

### Metabolic pathways within fGIs

Three fGRs (10, 18, and 24; Fig. 2b, Table 2) were identified whose predicted protein functions fell into central metabolism and biosynthetic pathway role categories. A number of enzymes of the aldehyde dehydrogenase family, such as vanillin dehydrogenase, acyl-CoA dehydrogenase, and succinate-semialdehyde dehydrogenase, were identified in fGIs. In bacteria, the action of alcohol dehydrogenase and aldehyde dehydrogenase on alcohol produces organic acids like acetic acid and eventually acetyl-CoA. The acetyl-CoA produced enters fatty acid metabolism and the tricarboxylic acid cycle. It has already been reported that low concentrations of ethanol can stimulate growth of *A. baumannii* and also increase its pathogenicity towards some organisms [62].

Additionally, enzymes for the breakdown of aromatic compounds indicate metabolic versatility in *A. baumannii* to possibly enable survival on alternative carbon, sulfur, and nitrogen sources [63]. For instance, homoprotocatechuate/hydroxyphenylacetate degradation (fGR 18) and phenylpropanoid degradation (fGR 9) pathways can provide intermediates for the tricarboxylic acid cycle. A phenylpropanoid/aromatic degradation pathway (fGR 9) was also previously mentioned as conserved catabolic regions (*pca-qui* genes) in *A. baumannii* strain AYE and *A. baylyi* strain ADP1 [54].

### House-keeping genes in fGIs

We also observed two house-keeping genes in fGIs (tRNA ligase genes and *tat*ABC system). tRNA ligases (a.k.a. aminoacyl tRNA synthetases or “aaRSs”) are typically single copy essential genes with rare instances of duplications seen in few bacteria, *Escherichia coli* [64] and *Bacillus subtilis* [65, 66] being two such examples. We found that 11 of the 249 sequenced *A. baumannii* genomes contain one or more tRNA synthetase duplications (*tyrS*, *cysS*, *thrS*; fGRs 18, 27), with three genomes carrying *cysS* and *thrS* duplications, and one genome with all three duplications. Twin-arginine translocation (Tat) system protein translocases TatA, TatB, and TatC [67] were observed in eight of the sequenced genomes (fGR 15), but we were unable to identify an effector protein with a Tat secretion signal that may have co-transferred with the *tatABC* operon.

### RIs in fGRs

Because RIs are composed of IS elements, composite transposons, and integrons, which are by definition mobile and therefore “flexible”, we predicted that our algorithm would identify them as fGIs, but it was unclear where they would insert into the core pan-chromosome. There are four known hot spots for insertion, including *comM* [26, 68–70], *pho* [37, 71], *astA* [69], and an acetyltransferase (acetylT) gene (a.k.a. HPA2 in [40]). Three fGRs (1, 10, and 22; Table 2) were discovered, corresponding to the known locations within or adjacent to *comM*, *astA*, and acetylT, respectively; however, we did not observe an fGR/fGI near *pho*. Drug resistance (DR) genes in RI-associated fGRs were only observed at the *comM* (fGR 1) locus, which comprised 13 of the 38 fGIs and 31 drug resistance genes (Additional file 10). In addition to the known RIs, a putative novel RI was discovered in an fGI 26,097 bp in length, encoding a metallo-beta-lactamase (ACIN5143_A3078 and ACINNAV18_0027) and located within fGR 27 (Table 2, Fig. 2b), residing in two military isolates sequenced in this study (OIFC143 and Naval-18).

### Identification of RI signatures

As RIs are made up of one or more transposable elements and most of the sequenced *A. baumannii* genomes are not finished and are, therefore, represented as multiple genomic contigs, RIs are often difficult to characterize. Even with the use of the novel pan-chromosome consensus-building algorithm described above, RIs appear to be fragmented and represented as multiple fGIs. Therefore, to better identify RI insertion events in draft genomes, a high-throughput bioinformatics approach was developed and implemented. This approach characterized RI signatures rather than complete RI structures. RI signatures are defined as both the genomic location and the type of RI insertion identified in an individual isolate. The approach searches for insertions within known RI insertion hot spots *comM*, *pho*, *astA*, and acetylT, and identifies homology with a group of carefully selected representative RIs to minimize redundancy from among those previously reported in *A. baumannii*, including AbaR3 [37], AbaR4 [70], AbGRI1 and AbGRI2 [69], and Tn*1548* [72] (Additional file 11).

**Fig. 3:**
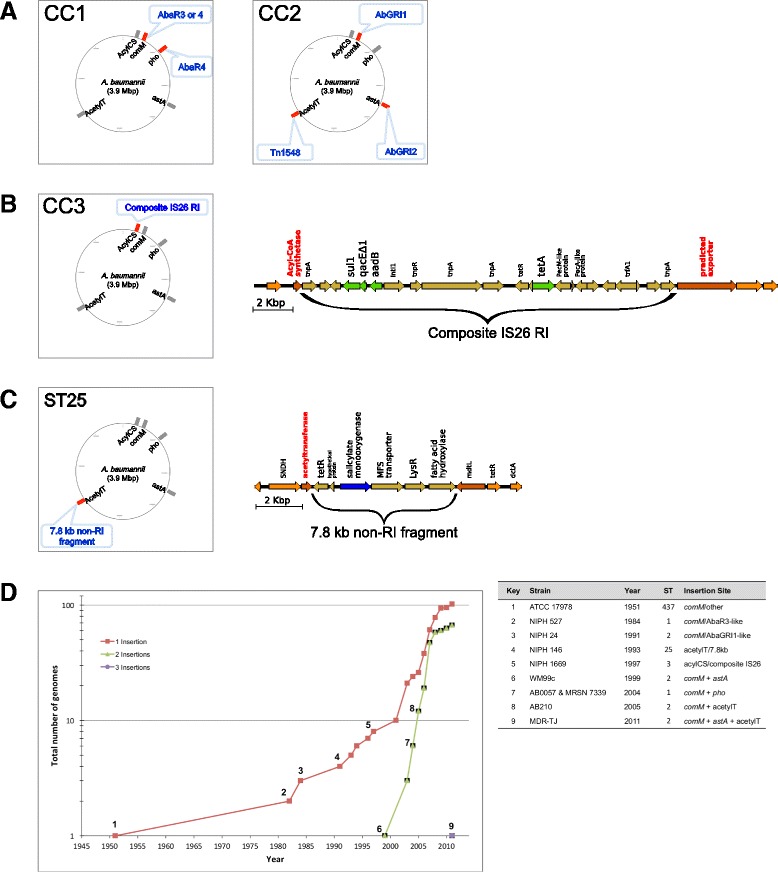
Clone-type specific RI signatures and RI insertion frequencies over time. **a–c** Genomic locations of RI signatures specific to clonal complexes (CCs) 1–3 and ST 25. In CC1 isolates, RI insertions are detected in the *comM* and/or *pho* loci (**a**). In CC2 isolates, RI insertions are found in the *comM* locus, and in addition the *astA*, or acetylT locus. A novel RI insertion (composite IS26) was identified in CC3 isolates at the acyl-CoA synthase (acylCS) locus (**b**). Gene annotation of the composite IS26 RI is shown to the right. Drug resistance genes (*green*); immediate flanking genes acylCS and a transporter protein (*dark orange*). A novel GI was identified in ST 25 isolates at the acetylT locus (**c**). Gene annotation of the 7.8 kb GI is shown to the right. Salicylate monooxygenase (*blue*); immediate flanking genes acetylT and *mdtL* (*dark orange*). A cumulative frequency graph showing the number of RI-positive isolates carrying single, double, or triple RI insertions collected between 1950 and 2011 (**d**). Isolates that represent the first occurrence of a given RI signature are labeled on the graph (numbered 1–9)

Using this bioinformatics approach, a total of 173 out of 247 (70 %) *A. baumannii* genomes analyzed were scored as RI-positive and assigned RI signatures (Additional file 12A–G). Individual clone types showed insertion site preferences and carried specific RI signatures (Fig. 3a–c). While RI insertions in *comM* were common among multiple clone types, insertions outside of *comM* were only detected at the *pho* locus in clonal complex 1 (CC1) isolates, and only at the *astA* or acetylT loci in CC2 isolates (Fig. 3a; Additional file 12b–d). Two distinct types of RIs were identified at the *comM* locus of RI-positive isolates: AbaR3 or AbaR4 in CC1 (22 out of 26 isolates) and predominantly AbGRI1 in CC2 (101 out of 105 isolates) (Fig. 3a; Additional file 12a, b). At non-*comM* loci, only a single type of RI insertion was detected; either AbaR4 at *pho* in CC1 isolates, or in CC2 isolates, AbGRI2 at *astA* or Tn*1548* at acetylT (Fig. 3a; Additional file 12b-d). Among the group of 123 CC1 and CC2 isolates identified to carry major RI signatures, 67 isolates (54 %) carried more than one RI insertion in the genome versus 56 (46 %) carrying single RI insertions (Additional file 12b).

To determine the mechanism of RI inheritance (i.e., vertical or horizontal) and to understand their evolution in individual clonal lineages, a whole genome single nucleotide polymorphism (SNP) tree was constructed for all isolates analyzed (including four non-*baumannii* outgroups) (Additional file 13). The SNP tree was defined by ~150,000 variant positions located on the backbone of the genomes by excluding regions with unusually high SNP density (Additional file 14).

Phylogenetic relationships of the isolates as shown by the SNP tree were similar to those in the BSR tree in that *A. baumannii* isolates were grouped by MLST type with exceptions for certain allelic differences within CC2. Many of the genomes that cluster between strains of the major STs were off by one allele from the major ST, making them a member of a CC [73]. However, MRSN 4106, 3405 and 3942 (i.e., ST94) differed from ST 1 by two alleles, suggesting possible horizontal gene transfer of MLST markers in these strains. It is clear from both the BSR tree and the SNP tree that the military isolates cover a spectrum of genome diversity, confirming the observed diversity via PFGE (Additional file 1).

When the RI signatures were superimposed onto the SNP tree, specific patterns of RI distribution were observed across different sequence types (Additional file 13). For example, the distribution of AbGRI1, which is predominantly found in CC2 isolates, appeared largely to be the result of vertical inheritance. It is interesting to note that an entire clade does not carry the AbGRI1 RI (triangle, Additional file 13). In contrast AbaR3, which is mostly found in CC1 strains, showed a more scattered pattern of inheritance with seemingly equal numbers with and without this RI. However, it should be noted that the absence of a detectable RI signature in this approach could be due to the incompleteness of the draft genome assemblies. Additional examples of apparent clonal or vertical inheritance were insertions in *pho* in a subgroup of CC1 isolates, two novel insertions discussed in the next section, including a 7.8 kb non-RI gene insertion in acetylT in the entire group of ST 25 isolates and a composite IS26 insertion in acyl-CoA synthase (acylCS) in the entire group of CC3 isolates (Additional file 13).

### Identification of novel RIs and GIs

During the analysis of RI signatures, we identified a novel RI insertion detected at a genomic region (ACICU positions 157,224–165,463 nucleotides) flanked by acylCS (ACICU_00319) and a predicted transporter protein (ACICU_00143). The novel RI replaces an 8 kb genomic region with an 18 kb RI identical to a previously reported composite IS26 transposon carrying a class I integron (GenBank accession JX041889) [74]. The composite RI carries two antibiotic resistance gene cassettes, including *sulI-qacEdelta1-aadB-intI1* (resistance to sulfonamides and gentamycin) and *tetR-tetA* (resistance to tetracycline). The gene structure of this novel RI is shown in Fig. 3b. A fragment of the RI was also detected within fGR “N” (Fig. 2b, Table 2). This composite IS26 RI was detected exclusively in all eight of the CC3 isolates analyzed, including six sequenced in this study (Additional file 12b, e). Seven of these isolates were MDR strains collected from the military healthcare system between 2003 and 2009 from wound, blood, catheter, or unknown sources. The earliest sequenced CC3 isolate was collected in the Netherlands in 1997 and contained only the 5′ fragment of the composite IS26 RI, which carried only one resistance gene cassette, *sulI-qacEdelta1-aadB-intI1*, rather than the full length version (Additional file 15).

In addition to the composite IS26 RI, we also identified a novel non-RI 7.8 kb genomic island (GI) juxtaposed to the acetylT locus in the absence of the Tn*1548* RI insertion commonly found at this location (Fig. 3c; Additional file 16). This novel non-RI insertion was also detected within fGR 22 (Fig. 2b, Table 2). The 7.8 kb GI shared over 90 % identity at the nucleotide level with *A. calcoaceticus* PHEA-2 and carried six annotated open reading frames (ORFs), including genes encoding a fatty acid hydroxylase and a salicylate monooxygenase. Salicylate monooxygenase, normally absent from the *A. baumannii* genome, is involved in the conversion of salicylate to catechol, which could possibly be used as a building block for the construction of catecholate-type siderophores. The acetylT/7.8 kb insertion was detected among all seven isolates of the non-major sequence type ST 25 (Additional file 12b, f). Two of these isolates, OIFC143 and Naval-18, were sequenced in this study.

### Evolution of RI insertion site usage from single to multiple RI insertions

To provide insight into how RI signatures and insertion site usage have evolved over time, insertion site usage was plotted by cumulative frequency (Fig. 3d). Three phases of site usage were observed, with a single RI insertion site in 1951, double insertions in 1999, and more recently, triple insertions in 2011 (Fig. 3d). During the first phase, single insertions were detected either at the *comM*, acetylT (7.8 kb) or acylCS loci. During the second phase, RI insertions were detected at *comM* in conjunction with a second insertion at *pho*, *astA* or acetylT. Finally, triple insertions were observed at *comM*, *astA*, and acetylT in the MDR-TJ isolate. These results showed a rapid increase in the number of RI insertions during the course of evolution of *A. baumannii* for antibiotic resistance. Analysis of additional genome sequences will help to further confirm the above observations.

### The gain and loss of virulence gene content

To better determine the presence or absence of specific gene clusters associated with virulence and survival, we studied the distribution and conservation of known virulence genes across all isolates. We detected the gain and loss of gene clusters at both the protein and nucleotide levels based on centroid-to-ortholog derived BSR analysis followed by whole genome sequence alignments. Among the ten classes of known virulence/survival mechanisms analyzed, including a collection of 178 genes (Additional file 17), three classes of genes (type I pili, siderophores, and efflux pumps) showed distinct gain/loss variations among the isolates. A heat map generated based on centroid-to-ortholog derived BSR is shown in Additional file 18 (BSR values in Additional file 19). A summary of the diversity of three virulence properties (i.e., adhesion, iron acquisition, and efflux) among the isolates analyzed is shown in Table 3 and Additional file 20.

**Table 3:** Diversity of virulence gene content across *A. baumannii* isolates

							Gene clusters			
Isolates	Genome category	Source	Year	ST	Allele summary	Country	1	2	3	4
**Type I pili**										
**AYE**	Global	Urinary	2001	1	1-1-1-1-5-1-1	France	+	+	+	
**ACICU**	Global	Internal	2005	2	2-2-2-2-2-2-2	Italy	+	+	-	
**SDF**	Global	Miscellaneous	2000	17	3-29-30-1-9-1-4	France	-	-	-	
NIPH 335	Global	Respiratory	1994	10	1-3-2-1-4-4-4	Czech Republic	-	+	+	
OIFC098*	WRAIR	Miscellaneous	2003	10	1-3-2-1-4-4-4	Germany	-	+	+	
**MDR-ZJ06**	Global	Blood	2006	2	2-2-2-2-2-2-2	China	-^1^	+	-	
UH clade B^2^	US hospital	Respiratory^3^	2007	2	2-2-2-2-2-2-2	USA	-^1^	+	-	
NIPH 60	Global	Respiratory	1992	34	8-1-14-3-12-1-13	Czech Republic	+	-	+	
NIPH 528	Global	Unknown	1982	2	2-2-2-2-2-2-2	Netherlands	+	+	-^4^	
**MDR-TJ**	Global	Miscellaneous	Before 2011	2	2-2-2-2-2-2-2	China	+	+	-^4^	
OIFC143*	WRAIR	Wound	2003	25	3-3-2-4-7-2-4	USA	+	+	-^4^	
**Siderophores**										
**AYE**	Global	Urinary	2001	1	1-1-1-1-5-1-1	France	+	-	+	-
**ACICU**	Global	Internal	2005	2	2-2-2-2-2-2-2	Italy	+	-	+	-
**SDF**	Global	Miscellaneous	2000	17	3-29-30-1-9-1-4	France	-	-	-	-
NIPH 190	Global	Unknown	1993	9	3-1-5-3-6-1-3	Czech Republic	-	-	+	-
NIPH 410 ^5^	Global	Blood	1996	39	10-4-3-2-13-1-2	Czech Republic	-	-	+	-
OIFC0162*	WRAIR	Respiratory	2003	412	1-52-2-2-67-4-5	USA	-	-	+	-
OIFC047* ^*5*^	WRAIR	Miscellaneous	2003	Novel	1-75-2-2-67-1-2	USA	-	-	+	-
Naval-82*	WRAIR	Blood	2006	410	3-1-2-3-6-1-16	USA	-	-	+	-
WC-348*	WRAIR	Skin	2008	412	1-52-2-2-67-4-5	Iraq	-	-	+	-
ATCC 17978	Global	Miscellaneous	1951	437	3-2-2-2-30-4-28	NA	+	+^6^	+	-
6013113	Global	Skin	2007	81	1-1-1-1-5-1-2	England	+	+	+	-
6013150	Global	Skin	2007	81	1-1-1-1-5-1-2	England	+	+	+	-
MRSN 3527*	MRSN	Wound	2011	81	1-1-1-1-5-1-2	USA	+	+	+	-
MRSN 3405*	MRSN	Wound	2011	94	1-2-2-1-5-1-1	USA	+	+	+	-
MRSN 3942*	MRSN	Wound	2011	94	1-2-2-1-5-1-1	USA	+	+	+	-
MRSN 4106*	MRSN	Wound	2011	94	1-2-2-1-5-1-1	USA	+	+	+	-
WC-487* ^*7*^	WRAIR	Skin	2008	410	20-26-26-14-26-16-23	Iraq	-	-	-	+^8^
**Efflux pumps**										
**AYE**	Global	Urinary	2001	1	1-1-1-1-5-1-1	France	+	+	+	
**ACICU**	Global	Internal	2005	2	2-2-2-2-2-2-2	Italy	+	+	+	
**SDF**	Global	Miscellaneous	2000	17	3-29-30-1-9-1-4	France	-	+	+	
NIPH 60	Global	Respiratory	1992	34	8-1-14-3-12-1-13	Czech Republic	-	+	+	
NIPH 80	Global	Blood	1993	37	3-2-2-2-7-1-2	Czech Republic	-	+	+	
NIPH 615	Global	Respiratory	1994	12	3-5-7-1-7-2-6	Czech Republic	-	+	+	
NIPH 410 ^5^	Global	Blood	1996	39	10-4-3-2-13-1-2	Czech Republic	-	+	+	
OIFC047* ^*5*^	WRAIR	Miscellaneous	2003	Novel	1-75-2-2-67-1-2	USA	-	+	+	
OIFC111*	WRAIR	Miscellaneous	2003	49	3-3-6-2-3-1-5	USA	-	+	+	
AB900	WRAIR	Skin	2003	49	3-3-6-2-3-1-5	USA	-	+	+	
AB_TG27343	Global	Wound	2005	422	26-72-2-2-29-4-5	USA	-	+	+	
AB_1536-8	Global	Unknown	2006	413	1-3-2-2-5-8-12	USA	-	+	+	
AB_1583-8	Global	Unknown	2006	422	26-72-2-2-29-4-5	USA	-	+	+	
Naval-72*	WRAIR	Wound	2006	405	5-3-16-4-29-1-60	USA	-	+	+	
ZWS1122	Global	Blood	2011	2	2-2-2-2-2-2-2	China	-	+	+	
ZWS1219	Global	Blood	2011	2	2-2-2-2-2-2-2	China	-	+	+	
**BJAB0715**	Global	Miscellaneous	5/2007–4/2008	23	1-3-10-1-4-4-4	China	-	+	+	

Isolate name: finished genomes (bold), pre-2000 isolates (underline), sequenced in this study (italics with asterisk)

Specific gain or loss of gene clusters with respect to majority of isolates analyzed and reference genomes AYE, ACICU, SDF shown as “+” and “-” signs, respectively

(1) A 42 kb deletion was detected in ST2 strain MDR-ZJ06 and the UH clade B isolates, in contrast to the 17 kb deletion observed in ST10 strains NIPH 335 and OIFC098

(2) Nine UH clade B isolates carry a deletion of the type I pili *csu* gene cluster [40]

(3) Six out of nine UH clade B isolates which showed a loss of the type I pili *csu* gene cluster are of respiratory origin

(4) Loss of type I pili cluster 3 was detected in 133 isolates, including ST2, ST25, ST79, ST113, and ST215. Only three isolates are shown. Similar gene loss was not detected in ST1 or ST3 isolates

(5) Two isolates (NIPH 410, OIFC047) had a dual loss of siderophore cluster 1 and efflux cluster 1 (AdeABC)

(6) Insertion of siderophore cluster 2 was detected at 3 Mb in ATCC 17978, which differed from the location identified in ST81 and ST94 isolates at 3.8 Mb (coordinates based on ACICU genome)

(7) WC-487 is a non-baumannii *Acinetobacter* sp. isolate

(8) Siderophore cluster 4 was also detected in *A. baumannii* 8399 [78, 80]

*ATCC* American Type Culture Collection, *NA* not available, *WRAIR* Walter Reed Army Institute of Research

**Fig. 4:**
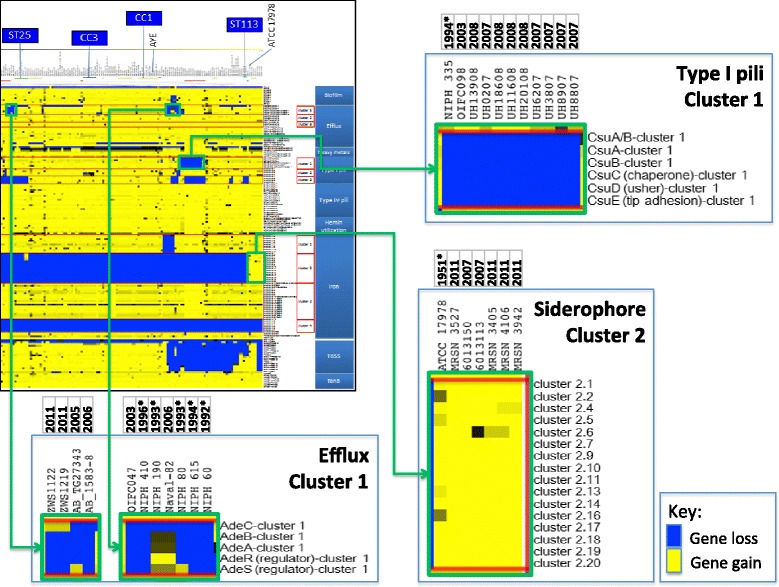
Virulence and fitness factors displaying variable gene content based on centroid-to-ortholog BSR. Specific genomic regions involved in the assembly of type I pili, siderophore production and efflux were highly variable and showed specific gain or loss of entire gene clusters in isolates analyzed. In the BSR-based heat map, the presence, absence, and low similarity of a protein ortholog compared with its centroid is shown in *yellow* (BSR = 1, presence), *blue* (BSR = 0, absence), and *gray* (low similarity or truncated), respectively. The year of collection is shown above strain names. Isolates collected prior to year 2000 are indicated with an *asterisk*. In summary, gene gain/loss events involved in virulence and survival were detected in decades-old isolates and appeared to have reemerged among recent isolates. The list of virulence genes analyzed and the full size version of the BSR-derived heat map are provided in Additional files 17 and 19, respectively

**Fig. 5:**
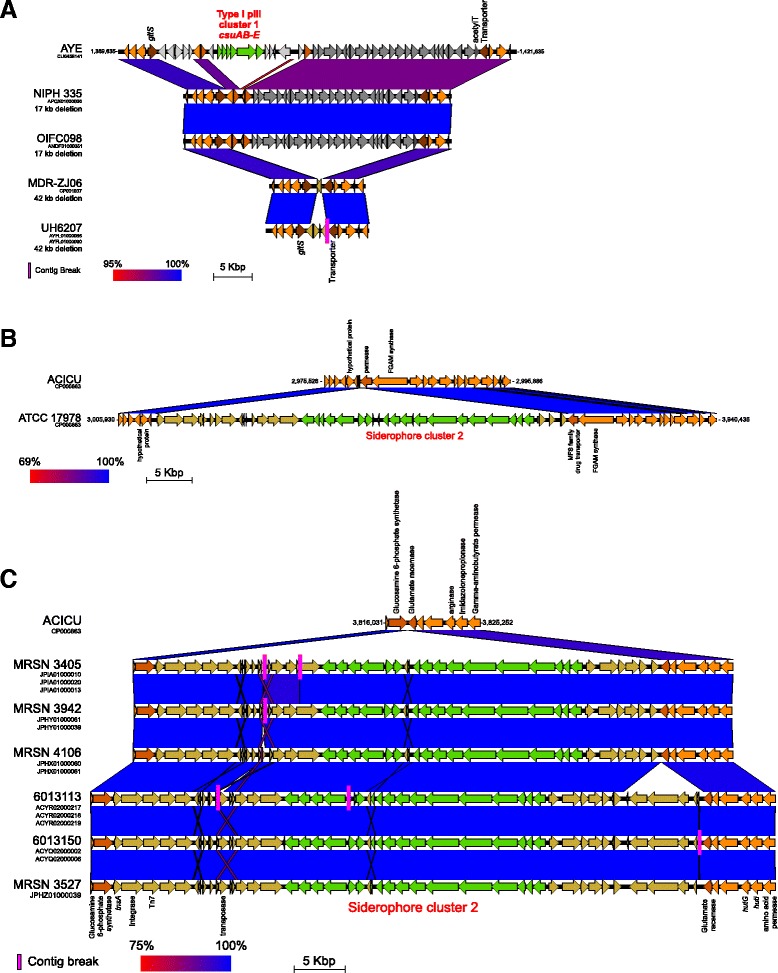
Loss of pili cluster 1 (*csu* gene cluster) and gain of siderophore cluster 2 in specific isolates. **a** Two types of deletion were observed which led to a complete loss of the type I pilus *csuAB-E* gene cluster. A novel 17 kb deletion was detected in NIPH 335 and OIFC098, whereas a previously reported 42 kb deletion was found in MDR-ZJ06 and nine UH clade B isolates (e.g., UH6207). **b** Siderophore cluster 2 was detected only in a small subset of isolates across all 249 analyzed. Two apparently independent molecular events were observed among the siderophore cluster 2-positive isolates. In decades-old isolate ATCC 17978, insertion of the gene cluster was detected at a genomic position corresponding to 3.0 Mbp of the ACICU reference genome. **c** In the remaining siderophore cluster 2-positive modern isolates (e.g., MRSN 3405), insertion was detected at a different location, which corresponds to 3.8 Mbp of the ACICU reference genome. Since ATCC 17978 was isolated in 1951 while other isolates were isolated more recently between 2007 and 2011, the acquiring of siderophore cluster 2 among modern isolates could be an example of the reemergence of a survival mechanism of *A. baumannii*. The functional significance of siderophore cluster 2 is yet to be determined. Key: pairwise nucleotide identity shown in *red* to *blue* (100 % identity) color scale; contig breaks (*pink vertical bars*); open reading-frames (*thick arrows*); type I pilus cluster 1 and siderophore cluster 2 genes (*green*); deleted genes (*gray scale*); genes bordering insertions/deletions (*dark orange* and *brown*); other flanking genes (*orange*); other genes (*light brown*)

The *csuAB-E* gene cluster has been shown to encode a chaperone-usher type I pili system [75] and is functionally characterized [76, 77]. *A. baumannii* also encodes two additional related type I pili clusters [78]. The presence of the *csuAB-E* gene cluster has been shown to be variable among relatively smaller subsets of *A. baumannii* genomes studied [31, 40, 78]. We observed the deletion of the *csu* gene cluster (i.e., type I pili cluster 1) only in certain ST 2 and ST 10 isolates as 42 and 17 kb deletions, respectively (Table 3, Fig. 4). Deletions of the *csu* gene cluster in ST 2 strains have been previously reported [40, 79], but the 17 kb deletion is a novel discovery. These *csu* deletions appeared to be the result of independent molecular events based on observations that the deletions occurred in different lineages as shown on the SNP tree (Additional file 13), and the distinct sizes of the deletions (Fig. 5a). Furthermore, type I pili cluster 2 was detected across all isolates except two strains, NIPH 60 and SDF. Type I pili cluster 3 was present among CC1 and CC3 isolates but absent from all ST 2, ST 25, ST 79, ST 113, and ST 215 isolates (Additional files 20 and 21; total = 113 isolates) as shown in the centroid-ortholog BSR-derived heat map (Additional file 18). It should be noted that by taking into account the overall genomic content of type I pili, strain MDR-ZJ06 and nine UH clade B isolates encoded a single type I pilus represented by cluster 2. The functional significance for type I pili expressed from different clusters is yet to be determined. Interestingly, six out of nine UH clade B isolates originated from respiratory samples, and all have been reported to be MDR (Table 3).

Siderophores are iron uptake machinery for bacterial survival and virulence under limiting iron conditions and are encoded in five known clusters/genomic islands in *A. baumannii* [78, 80]. We observed that ST 1 (e.g., AYE), ST 2 (e.g., ACICU) and most isolates analyzed in general carried siderophore cluster 1 (A1S_1647 to A1S_1657) and cluster 3 (A1S_2372 to A1S_2392) (Table 3; Additional file 18), which were also part of the core pan-genome. However, siderophore cluster 1 was missing in four US military Walter Reed Army Institute of Research (WRAIR) isolates sequenced in this study (strains OIFC0162, OIFC047, Naval-82, and WC-348) and two additional isolates (NIPH 190 and NIPH 410) (Table 3; Additional file 22). Despite belonging to different sequence types, all six isolates shared close phylogenetic distances as shown on the SNP tree (Additional file 13). We showed that siderophore cluster 3, encoding the key *A. baumannii* siderophore acinetobactin, was detected among all isolates analyzed except SDF (from body louse) and the non-*A. baumannii* isolate WC-487.

In addition, we also observed the acquisition of a siderophore gene cluster among specific isolates. Siderophore cluster 2 was rarely found in *A. baumannii* and only previously reported in two isolates: ATCC 17978 (Figs. 4 and 5b) collected in 1951 and *A. baylyi* ADP1 [78]. Siderophore cluster 2 was also found on an fGI (Assembly_fGI 41, Additional file 8). In our analysis, we detected cluster 2 in six additional isolates belonging to ST 81 and ST 94 collected between 2007 and 2011 (Table 3, Figs. 4 and 5c). The six isolates shared a common insertion site for siderophore cluster 2 at 3.8 Mbp different from that of ATCC 17978 at 3.0 Mbp (reference genomic coordinates were based on the ACICU genome, which does not carry the insertion). The six isolates are also phylogenetically distinct from ATCC 17978 as shown on the SNP tree (Additional file 13). Among the six isolates, four were isolated from wound samples of the US military MRSN collection sequenced in this study. Further studies are needed to determine if siderophore cluster 2 is associated with different iron availability in military wound samples. Lastly, siderophore cluster 4, which was previously identified in *A. baumannii* isolate 8399 [78, 80], was only identified in one isolate in this study, the non-*A. baumannii* isolate WC-487 (Table 3).

Efflux pumps are outer membrane proteins that drive the expulsion of antimicrobials leading to resistance against aminoglycosides, β-lactams, chloramphenicol, erythromycin and tetracycline [81]. We noted that the AdeABC efflux (A1S_1823 to A1S_1825) gene cluster was deleted in a small set of isolates across multiple strain types (Table 3). Similar to SDF, two isolates, OIFC047 and NIPH 410, which are phylogenetically closely related as shown on the SNP tree (Additional file 13), showed a dual loss of the AdeABC efflux cluster and the siderophore cluster 1 (Table 3). Determining the functional consequence of the gene loss will aid in the characterization of the significance of these specific virulence determinants.

## Discussion

In this study, the draft genome sequences of 50 *A. baumannii* isolates from the military healthcare system were determined and analyzed within the framework of a 249 isolate pan-genome, to identify the genetic determinants underlying MDR and virulence properties in the context of strain diversity and evolution. Using a novel graph-based approach, we identified highly variable and dynamic genomic content of the *A. baumannii* genome, which may be the result of its rapid adaption and survival in both biotic and abiotic environments through the gain and loss of gene clusters controlling fitness. Importantly, our results show that some of the adaptation mechanisms (e.g., gain/loss of pili and siderophore gene clusters) existed in decades-old isolates and appeared to have reemerged among recent isolates. This study will provide a valuable framework and genetic landmarks for surveillance, prediction of outbreaks, and understanding the epidemiology of globally distributed isolates.

### *A. baumannii* pan-genome

To determine whether the genomic diversity of *A. baumannii* has been captured among all sequenced isolates (i.e., a closed pan-genome) and to understand how the 50 selected military isolates were evolutionarily related to previously sequenced isolates, we conducted, to our knowledge, the first *A. baumannii* pan-genome analysis on the most expansive set of isolates, including 249 genomes. We observed 1867 core (100 % membership), 2833 core (95 % membership) protein clusters and a paralog-collapsed pan-genome cluster size of 11,694 proteins. For comparison, in a pan-genome study of 186 *E. coli* strains (~1 Mbp larger than *A. baumannii* and ~1000 more genes per genome), there were 1702 core (100 %), 3051 core (95 %), and a pan-genome cluster size of 16,373 proteins [82]. This shows that even though the average genome size of *E. coli* is larger by ~1 Mbp, the core pan-genome cluster size is similar to *A. baumannii*. However, the larger pan-genome cluster size observed in *E. coli* (by ~5000 proteins) may reflect a higher proportion of variable/flexible regions within the pan-genome of *E. coli* compared with *A. baumannii*.

### Pan-genome open or closed?

Our initial analysis of all 249 genomes suggested that the pan-genome was closed; however, after determining that around half of the genomes were from highly related strains of MLST CC2, we tested whether inclusion of highly similar strains can alter the pan-genome state (e.g., open versus closed). We used hierarchical clustering to normalize the diversity of strains chosen for inclusion and showed that the pan-genome was open when restricting to a diverse set of 100 genomes. To test whether this was the result of undersampling rather than removal of highly similar genomes, we conducted a parallel analysis on about 100 CC2 isolates and showed that the pan-genome was closed. These results suggest that the inclusion of the entire set of 249 strains in the pan-genome state calculation can bias the outcome, resulting in a closed pan-genome. We concluded that including many closely related strains (i.e., from an outbreak) in a pan-genome study could bias the results of the pan-genome state (open versus closed). We suggest using a normalization step to choose strains for inclusion in the study or taking a bootstrapping approach as we did: first run all genomes to identify ortholog/paralog clusters, build a BSR tree, normalize isolate collection for diversity, then re-run the analysis a second time using the final strain list. The bootstrap approach may also be useful in situations where a non-target contaminant strain has been sequenced or an isolate has been misidentified, thus also serving as a quality control step.

### Assembly of core proteins and fGIs into a pan-chromosome

To facilitate analysis and interpretation of this large pan-genome dataset, an unsupervised approach was developed and implemented through a novel graph-based algorithm to assemble ortholog clusters of core proteins (75 % core definition) into the first reference-independent consensus core “pan-chromosome” of a bacterial species. This formed the foundation for the identification and placement along the core pan-chromosome of fGIs that are highly flexible and variable across the group of isolates. Both circular and linear “assemblies” were produced, where the core pan-chromosome clusters assembled into a circle of 3126 genes, which is roughly the size estimated for the 95 % core definition. The linear cluster assemblies were the fGIs that can be placed on the core pan-chromosome, making up fGRs, while the non-core circular cluster assemblies were identified as plasmids and circular phage genomes. There is increased interest in these extra-chromosomal prophages as sources of virulence factors [83] and as vehicles for rapid adaptation to changing environments in a “carrier state” [84]. The circular nature of these extra-chromosomal phage genomes is often not observed; however, our novel assembly algorithm can distinguish both circular and linear forms of prophages as circular and linear fGIs, respectively.

Overall, analysis of genes encoded on fGIs confirms previously identified catabolic diversity and reiterates the versatility and adaptation of *A. baumannii* to survive and thrive in a variety of environments where nutrients are scarce. The occurrence of alcohol and aldehyde dehydrogenase genes in fGIs of hospital-isolated strains could be an indication of the ability of *Acinetobacter* to thrive in the presence of ethanol disinfectant reagents. With regard to the unexpected finding of additional copies of essential housekeeping genes such as the tRNA synthetases, we do not know the function of these additional copies or the purpose for having additional copies. Experiments will have to be conducted to determine whether these fGIs carrying the aaRS genes can complement the core aaRS genes and under what conditions they may be expressed.

### Virulence factor diversity and strain fitness for host survival

It is noteworthy that among the ten classes of genes and gene clusters previously shown to be associated with virulence and fitness, to our surprise, a high genomic diversity was observed in genes involved in adhesion (type I pili assembly), iron acquisition (siderophore production), and efflux pumps among the 249 isolates studied.

Functional characterization of type I pili cluster 1 (*csuAB-E*, A1S_2213 to A1S_2218) has shown that its expression is required for biofilm formation and attachment to abiotic surfaces such as plastic medical devices (e.g., ventilator tubes and catheters). Interestingly, a *csu*-deficient strain showed a loss of long appendages while retaining short pili on the cell surface and also enhanced attachment to an increased number of bronchial epithelial cells [75]. As previously reported [36], we also observed a relatively higher incidence of *csu*-deleted isolates in respiratory samples among the UH clade B isolates which belong to CC2. It is tempting to hypothesize that the loss of the *csu*-encoded pili is related to niche specialization for increased invasiveness or enhanced survival at specific sites of infection, such as the respiratory epithelium.

Siderophores are iron-scavenging systems utilized by pathogens to survive in mammalian host environments. Besides siderophore gene cluster 3 (which encodes the well characterized acinetobactin system), it is unclear what types of siderophores (e.g., catecholate, phenolate, hyroxamate, carboxylate or mixed) are produced from the other three siderophore clusters in the *A. baumannii* genome. It is conceivable that the acquisition of cluster 2, specifically among the four US military MRSN wound-isolates, is to produce a novel or stealth type of iron scavenger to circumvent host iron defense systems (e.g., catechol-type siderophore inhibitor siderocalin [85]) or outcompete other bacteria.

Also potentially pertinent to iron scavenging, a novel 7.8 kb non-RI GI with a best match to the environmental isolate *A. calcoaceticus* PHEA-2 was identified at the acetylT hot spot among all seven ST 25 isolates analyzed. One of the ORFs located on the 7.8 kb GI encodes salicylate monooxygenase, which converts salicylate to catechol. In principle, catechol can directly serve as an iron carrier or building block for siderophore synthesis. Functional analysis will be necessary to determine whether the specific acquisition of this salicylate monooxygenase can increase the capacity for iron acquisition, making it a novel mechanism that can reinforce and diversify siderophore production in this pathogen.

WC-487 is one of the strains sequenced in this study and originally thought to be *A. baumannii*. Both WC-487 and SDF showed a general loss or absence of genes for the virulence factors analyzed. The lack of key virulence factors in the human louse strain SDF supports the idea that although currently classified as *A. baumannii*, SDF has adapted to a life style different from that of a human pathogen. For WC-487, multiple lines of evidence, such as placement on both the BSR and SNP trees and the absence of key virulence determinants, suggest that WC-487 is truly not *A. baumannii*. Indeed, matrix assisted laser desorption ionization time-of-flight (MALDI-TOF) mass spectrometry results suggest that WC-487 instead belongs to *Acinetobacter nosocomialis* (X-Z Huang, manuscript in preparation).

### Dynamics of drug resistance genes and RIs

Drug resistance genes are acquired via IS elements and small composite transposons. The association with IS elements, which are repetitive and classically result in the misassembly of sequence data, are also problematic during the assembly of protein clusters. Using the fGI approach, we only detected drug resistance genes associated with an RI in the *comM* hot spot, but not the other three hot spots. Even with this limitation, we were able to identify three of the four known RI insertional hot spots as fGRs. Our algorithm was also able to identify a potentially novel RI, encoding a putative metallo-beta-lactamase in two of our sequenced military isolates. We identified a ~38 kb fGI within the *astA* region that is similar in size to the ~40 kb deletion that is known to have occurred in some strains [40], which highlights the point that fGIs can be insertions or deletions.

Interestingly, analysis of the drug resistance profiles and genome sequences of the military isolates revealed a potentially novel *parC* mutation (Glu88Lys) in strain Naval-83, which could be associated with quinolone resistance in *A. baumannii*. This mutation has been shown to confer resistance to a third generation quinolone (levofloxacin) in *Haemophilus influenzae* [36] and may, therefore, by analogy also do so in *A. baumannii*. Incidentally, we also observed this same mutation in *A. baumannii* 1656-2 [86]; however, its resistance to levofloxacin was not communicated, stressing the need to publish antibiotic drug resistance profiles alongside genomic data.

### Vertical and horizontal transmission of RIs

Two major questions of RI transmission are whether they are vertically or horizontally acquired and in how many genomic locations they can reside. Since the presence of IS elements resulted in fragmented genomic and pan-chromosome assemblies, we developed a high-throughput three-step bioinformatics approach to define the type and location of RIs in individual isolates to answer these questions. The approach included the identification of gene fragments at insertion hot spots, recruitment of genomic contigs using RI references and confirmation for the presence of antibiotic resistance gene cassettes. Based on the classification of isolates by RI signatures and phylogenetic distance defined by a SNP tree, our results revealed that clonal expansion and vertical inheritance of specific RI signatures are commonly observed (e.g., CC1, CC2, CC3, ST 25). Additionally, the accumulation of RIs at multiple hot spots within an isolate also suggested a combined dual mode of transmission that includes both vertical transmission of the *comM*-RI and horizontal acquirement of RIs at secondary locations.

### Dynamics of RI insertions and virulence/fitness determinants

Since the *A. baumannii* isolates analyzed in this study were collected throughout several decades between 1951 and 2011, we had an opportunity to follow the evolution of genomic determinants such as RIs and virulence factors during this timeframe. Comparing single to multi-RI existence in an individual genome, the ratio is 6:1 (n = 7) in pre-2000 isolates versus 0.9:1 (n = 125) in post-2000 isolates. Despite the limited sample size within the pre-2000 group, there is a prevalence of multi-RI insertions among modern isolates. Specifically, by considering the same set of insertional hot spots among pre-2000 and post-2000 isolates, the post-2000 isolates have a higher prevalence of these sites occupied, which likely resulted from selective pressure from the increased use of antibiotics in recent years and possibly higher sampling rates post-2000. There were also strains with no RI insertion detected. Considering the draft status of most genomes analyzed, more isolates need to be finished, particularly for older isolates that are not well represented.

It is interesting to note that the earliest gain or loss events can be traced back to isolates collected from two or more decades ago. For example, the lack of different type I pili clusters were first observed in strains NIPH 528 (ca. 1982), NIPH 60 (ca. 1992), and NIPH 335 (ca. 1994), which are among the oldest strains in this dataset (Additional file 20). Similarly, the earliest isolates showing the presence of siderophore cluster 2 and the absence of siderophore cluster 1 were ATCC 17978 (ca. 1951) and NIPH 190 (ca. 1993), respectively. These results suggest the early existence of genetic determinants controlling virulence and pathogenesis in decades-old isolates and their recent reemergence amongst modern isolates as shown in this study.

## Conclusions

We conducted the largest bacterial pan-genome analysis (249 genomes) of *A. baumannii* and determined that this pan-genome is open when the input genomes are normalized for diversity. A novel graph-based algorithm was developed and implemented to assemble ortholog clusters of core proteins into the first reference-independent “pan-chromosome” of a bacterial species, which was essential for mapping fGIs to fGRs. We concluded that the observed PFGE diversity of the 50 selected military isolates was mostly due to differences in fGI content rather than chromosomal rearrangements as no rearrangements of large contigs were detected; however, our ability to detect rearrangements is limited due to the fragmented nature of the genome assemblies.

We utilized a comparative genomics approach to analyze the diversity of RIs and virulence factors of *A. baumannii*. We demonstrated the existence of novel RIs and isolates with an increased number of RI insertions over time. Clusters of genes for carbon utilization, siderophore production, and pili assembly were highly variable, which may contribute to the success of *A. baumannii* in surviving and adapting to different and changing environments. A vast collection of genetic determinants and mechanisms to control antibiotic resistance and survival adaptations existed in decades-old isolates, and these genetic mechanisms appear to have reemerged among modern isolates, sometimes in different genomic locations. The comprehensive comparisons of the highly variable and flexible genomic features in the context of whole genome phylogeny will serve as genetic landmarks for surveillance and prediction of outbreaks, understanding the epidemiology of globally distributed isolates and identifying clonal origins of nosocomial infections of *A. baumannii* across healthcare institutions.

## Materials and methods

### Ethical statement

Per WRAIR Policy 12-09, the use of bacterial isolates without associated human data does not require a determination from the institutional review board or Human Subjects Protection Branch, the corresponding regulatory office.

### Strain isolation and verification

All 50 strains sequenced in this study were isolated at US military healthcare facilities [15, 87, 88] and identified as *A. baumannii* by standard automated biochemical analysis as described previously [8]. PFGE and 16S rRNA typing was also used to further validate species-level classification from genomic DNA prepared as described [20].

### Antimicrobial susceptibility tests

Antimicrobial susceptibility tests were performed on all isolates at the Walter Reed Army Medical Center clinical laboratory using the commercially available BD Phoenix NMIC/ID133 panel (Becton, Dickinson and Company, Franklin Lakes, NJ, USA). Susceptibility was determined according to Phoenix criteria and CLSI M-100-S-19, Vol.29, No.3 2009. For MRSN 58, antimicrobial susceptibility tests were performed using the commercially available Siemens MicroScan panel.

### Genome sequencing

The genomes of 50 *A. baumannii* isolates were sequenced at JCVI by Illumina HiSeq (2 × 100 bp), or a combination of Illumina HiSeq and 454 FLX Titanium. Additionally, MiSeq (2 × 150 bp), IonTorrent PGM, 454 libraries, and OpGen optical restriction maps generated by WRAIR were available to aid in gap closure for certain MRSN strains. Briefly, paired-end libraries were constructed for each sequencing technology from randomly nebulized genomic DNA in the 300–800 bp (Illumina) and 2–3 kb (454) size ranges following manufacturer recommendations. Sequence reads were generated with a target average read depth of ~20–30 fold (454) and ~60 fold (Illumina) coverage.

### Draft genome assembly

Sequences for the non-MRSN isolates were assembled using the Celera Assembler version 6.1 [89]. Assembled contigs undergoing further genome finishing (n = 10) and automated gap closure (n = 7) were ordered based on alignment against the best-matching complete *A. baumannii* reference genome using NUCmer [90]. Mapped contigs were never broken even if the contig matched different regions of the reference genome — the longest match was used for placement. Mapping merely entailed ordering and orienting the contigs with small spacers inserted between the contigs. As a result, all core gene adjacency information within the contigs was retained. Ten of the 42 genomes underwent manual gap closure to elevate the genome status to IHQD (Table 1).

For the seven MRSN isolates, we explored several assembly strategies to integrate the JCVI Illumina HiSeq data with data generated through various sequencing platforms by WRAIR. We decided to employ a pipeline that combined *de novo* assembly followed by automated reference-guided gap closure to resolve short and uncomplicated gaps <3.5 kb in length. JCVI sequence reads were assembled with Velvet version 1.0.19 [91] and optimized using the VelvetOptimiser 2.2.0 [92]. The Velvet assembly served as the backbone while other *de novo* assemblies of the WRAIR libraries built with Celera assembler version 7.0 or Velvet version 1.0.19 served as references from which the gap sequences would be predicted. In the first round of gap closure, optical maps (OpGen) were used to validate the assembly as well as to order and orient the backbone contigs using SOMA [93]. Automated gap closure consisted of the following processes: 1) to determine gap regions, consecutive contig ends were identified by alignment against the consensus sequence generated from various *de novo* assemblies using NUCmer [90]; 2) the identified contig ends were used to recruit reads from the JCVI Illumina paired-end library using Burrows-Wheeler Aligner (BWA) 0.7.3 [94]; 3) the recruited reads were assembled using the CLC command line tool *clc_mapper* from *clc-assembly-cell* v.4.0.11 [95] by mapping the recruited reads from step 2 to the gap regions from step 1 to generate a new consensus sequence for each of the gaps; 4) the contigs, and if available, the new gap sequences from step 3, were stitched together to resolve the gaps; 5) the CLC *clc_find_variations* command line tool, also from *clc-assembly-cell* v.4.0.11, was run to validate the new consensus sequence by determining the existence of any 0× coverage regions. If any 0× regions were found, the original gap remained. BLAST v.2.2.28 [96] was then used to select the closest matching complete *A. baumannii* genome in GenBank to serve as the reference for scaffolding the resulting contigs from the first round of the automated gap closure, using NUCmer [90] alignments. The contigs then proceeded through a second round of the automated gap closure process.

### Annotation

Contigs were annotated for protein- and RNA-encoding features using the JCVI automated annotation pipeline essentially as described previously [44, 47, 97, 98] except hidden Markov models were run using HMMER3 [99].

### Identification of antibiotic resistance genes

Genes conferring drug resistance were identified using the RGI (Resistance Gene Identifier, version 2) tool in CARD (Comprehensive Antibiotic Resistance Database) [100]. For each genome in this study (Table 1) a multi-FASTA composite file was loaded into RGI and the output saved for further parsing. Results were filtered by selecting the highest percent identity match for each ORF. Genes that were regulators or modulators were filtered out. Genes identified were classified by their antibiotic resistance ontology assigned by CARD; ontologies are based on resistance mechanisms, determinants and targets.

Several other genes were identified by BLAST analysis. A database of additional drug resistance genes was compiled from the GenBank accessions of previously curated lists [17, 101]. Genomes were searched against this database using BLASTP and unique ORFs not already identified by RGI were examined. Matches with >90 % amino acid identity were assigned a classification.

### MLST analysis

MLST was determined using an in-house automated pipeline that first searches for homologs of each gene of the typing schema (*cpn60:fusA:gltA:pyrG:recA:rplB:rpoB*) from [73, 102], using BLASTN [96]. MLST homologs were extracted from the genome sequence and compared with an MLST allele database to generate the allele number and ST for each genome.

### Pan-genome analysis

Clusters of orthologous proteins were generated (Additional file 5) by version 3.18 of PanOCT [35] using default parameters (Additional file 23). In order to plot “power law and exponential regressions for new genes discovered with the availability of additional genome sequences”, as defined by Tettelin et al. [41], we adapted the R scripts, *compute_pangenome.R* and *plot_pangenome.R*, from Park et al. [103] and developed a Perl script, *paralog_matchtable.pl*. Since PanOCT does not place paralogs into its ortholog clusters, but does produce a *paralogs.txt* file that specifies which clusters are paralogs, an in-house PERL script, *paralogs_matchtable.pl*, was created to merge paralogous clusters (Additional file 23). This is necessary because analysis of core and novel genes has historically been defined for clusters containing all paralogs [42, 103–107]. In the past, core and novel pan-genome plots were computed from all possible combinations in genome order, but this is computationally prohibitive when the number of genomes is over 100. To overcome this limitation, *compute_pangenome.R* was modified to randomly sample without replacement a subset of 500 combinations in genome order of addition. The output of this script is a set of data where each row contains columns for core, dispensable, unique, and genes novel for the last genome added. The *plot_pangenome.R* script computes the medians of the *compute_pangeome.R* output and uses the nonlinear least squares, *nls*, function in R to find power law and exponential models to fit the medians.

Consensus assemblies of the core and the flexible parts of the *A. baumannii* pan-genome were calculated using outputs from PanOCT. The consensus core pan-chromosome was computed by running an in-house PERL script, *gene_order.pl*, using the PanOCT *75_core_adjacency_vector.txt*, *0_core_adjacency_vector.txt*, and the centroids.fasta output files as input (Additional file 23). The *75_core_adjacency_vector.txt* file lists the set of adjacent core gene clusters (called “adjacencies”) and specifies which genomes contain them. Core gene clusters are defined as gene clusters conserved in at least some threshold number of genomes (e.g., 75 %). A core gene cluster is adjacent to another core gene cluster in a given genome if the representative cluster members for that genome are adjacent (i.e., they are on the same contig and have no other core genes between them). The consensus assembly of the core gene clusters is the set of adjacencies supported by the largest number of genomes (Additional file 8).

Conceptually, the order and orientation of these clusters can be depicted as linear or circular arrangements, analogous to sequence assembly. The linear paths can result from contig breaks, linear chromosomes or plasmids, or because there is a disagreement in the juxtaposition of neighboring core clusters between two or more genomes. The circular paths can represent circular chromosomes, plasmids or occasionally small elements that are inverted in different genomes.

fGIs were defined in [50] as GIs encoding similar types of functions (e.g., O-antigen, phage, pili), having the same genomic location, but a variable gene content. We define fGIs more loosely to be variable (i.e., “flexible”) linear assemblies of noncore genes present between core gene clusters. These assemblies were constructed and the fGIs identified using the same *gene_order.pl* script; however, the PanOCT output file *0_core_adjacency_vector.txt* is used (0 % threshold) as input so that all gene clusters are considered, not just core gene clusters (Additional file 9). The fGIs are not allowed to extend into core gene clusters already in the core pan-genome; rather, they are terminated at a core gene cluster and the core gene cluster is labeled as an fGI insertion site.

### Pan-genome tree

A UPGMA (unweighted pair group method with arithmetic mean) tree was constructed using the mean of the BSR as described previously [108]. The PanOCT output file *100_pairwise_BSR_distance_matrix_phylip.txt* was used as input for Neighbor [109, 110] to build an unrooted tree. This PanOCT output file is a Phylip-style distance matrix derived from the pairwise mean BSR of core proteins present in 100 % of genomes where a single value is presented for each pair of genomes in the pan-genome.

### SNP tree

A phylogenetic tree was inferred from SNPs identified among 253 *Acinetobacter* genomes. SNPs were identified by kSNP [111] with a requirement that at least 80 % of the genomes (i.e., 203 genomes) have a nucleotide at a given SNP position in order for the SNP to be considered for inclusion in downstream analysis. A total of 207,619 identified SNP positions were further filtered to remove SNPs in regions likely undergoing recombination by detecting regions with unusually highly SNP density. For this filtering step, a set of pairwise SNPs was identified between the finished genome of *A. baumannii* ACICU and related ST 2 genomes using the SNP export functionality within progressiveMauve [112]. The pairwise SNP density was computed based on ACICU positions shared among a subset of genomes with the fewest total number of pairwise SNPs. Any regions with higher than 10 SNPs/kb for any strain were considered as potential recombination regions. After filtering out these regions there were 152,995 presumed non-recombinant SNPs. These SNPs were used to generate a maximum-likelihood tree using RAxML [113] with 100 bootstrap replicates.

### Identification of RI signatures

A high-throughput approach was developed to identify RI signatures for 249 isolates, including draft genome assemblies. In draft genomes, RIs are often poorly assembled due to repetitive elements. In this approach, the RI signatures were determined based on: (i) junction fragments of target genes, (ii) sequence similarities to previously reported RIs and homology to antibiotic resistance cassettes within individual RIs. First, the presence of target gene junction fragments was identified using a BLASTN search against five target genes known to harbor RIs in *A. baumannii* (Additional file 11). A summary of target gene lengths when intact or carrying junction fragments in the presence of RI insertion is shown in Additional file 12h. Second, contigs identified to carry the target gene and junction fragments were searched against a collection of carefully selected RIs, which captures the diversity of RIs identified to-date (Additional file 11), using BLAST and NUCmer [90]. RI signature assignments were performed based on BLAST similarity and mummerplot alignments to the set of reference RIs. An example RI signature assignment for *comM* is shown in Additional file 12i.

### Gain/loss of virulence factor gene clusters

For every gene of interest, the average BSR value between the centroid protein of the ortholog cluster and the ortholog protein identified in each isolate were obtained from the PanOCT output (e.g., *pairwise_in_cluster.txt* and *centroids.fasta*). We use the term “centroid”, which is technically the medoid, to be a representative protein sequence of a cluster whose average dissimilarity to all the sequences in the cluster is minimal [114]. An average BSR value close to 1 indicates identical sequences, a value near 0 indicates absence of the protein in the given isolate, and a value around 0.5 suggests a truncation of the protein with respect to the centroid. A matrix of averaged BSR values between centroid-ortholog pairs was constructed with genes as rows and isolates as columns (Additional file 19).

To identify initial evidence of gain or loss of gene clusters at the ortholog protein level, the centroid-to-ortholog BSR matrix was analyzed using hierarchical clustering in MeV version 4.9.0 [115]. Additionally, regions of gene cluster gain/loss and flanking genomics sequences were then examined at the genomic contig level using pairwise sequence alignments by MUMmer version 3.0 [116]. Alignments of genome contigs comparing selected reference genomes and isolates of interest to show gain/loss of gene clusters were plotted using EasyFig [117]. The set of *A. baumannii* virulence genes analyzed is shown in Additional file 17. The genomic locations of virulence genes and RI insertions studied are summarized in Additional file 24.

### Software, genome sequences, and raw data availability

The pan-genome analysis software developed and implemented in this study, including *panoct.pl* version 3.18, *paralog_matchtable.pl*, and *gene_order.pl* are publicly available in the code section of the PanOCT SourceForge page [118], under the GNU General Public License.

GenBank accession numbers for the 50 genomes that were sequenced, assembled and annotated in this study are listed in Table 1 and Additional file 2. The whole genome sequencing data are available at [119] under the following accession numbers: AFCZ00000000, AFDA00000000, AFDB00000000, AFDK00000000, AFDL00000000, AFDM00000000, AFDN00000000, AFDO00000000, ALAL00000000, ALII00000000, AMDE00000000, AMDF00000000, AMDQ00000000, AMDR00000000, AMEI00000000, AMEJ00000000, AMFH00000000, AMFI00000000, AMFK00000000, AMFL00000000, AMFP00000000, AMFS00000000, AMFT00000000, AMFU00000000, AMFV00000000, AMFW00000000, AMFX00000000, AMFY00000000, AMFZ00000000, AMGA00000000, AMGE00000000, AMGF00000000, AMGG00000000, AMGH00000000, AMSW00000000, AMSX00000000, AMSY00000000, AMSZ00000000, AMTA00000000, AMTB00000000, AMZR00000000, AMZT00000000, AMZU01000000, JPHV00000000, JPHW00000000, JPHX00000000, JPHY00000000, JPHZ00000000, JPIA00000000, JPIB00000000. The raw sequence reads generated at JCVI are available at [120] under the following accession numbers: SRR1945422, SRR1945425, SRR1945426, SRR1945427, SRR1945428, SRR1945430, SRR1946597, SRX027591, SRX027592, SRX027593, SRX027595, SRX027596, SRX027597, SRX031272, SRX031273, SRX031275, SRX032336, SRX101596, SRX101598, SRX101601, SRX101602, SRX101603, SRX101604, SRX101606, SRX101793, SRX101794, SRX101795, SRX101796, SRX101797, SRX101798, SRX101800, SRX110040, SRX110090, SRX110091, SRX110092, SRX110095, SRX110096, SRX110097, SRX110098, SRX110099, SRX110100, SRX110101, SRX110102, SRX110103, SRX110104, SRX110105, SRX110106, SRX110107, SRX110109, SRX110110, SRX110111, SRX110112, SRX110113, SRX110114, SRX110115, SRX110118, SRX110119, SRX110120, SRX110121, SRX110122, SRX110123, SRX110124, SRX110125, SRX110126.

The combined JCVI-annotated fasta-formatted amino acid sequences and combined attribute files required for *panoct.pl* are available as Additional files 25 and 26, respectively. An essential set of raw output files from *panoct.pl* and *gene_order.pl* is also available in Additional file 27.

## Additional files

Additional file 1: Figure S1. Pulse field gel electrophoresis (PFGE) of *A. baumannii* isolates collected from the Military healthcare system. A dendrogram was produced based on the analysis of PFGE banding patterns. Genomic DNA was digested with ApaI, separated by clamped homogenous electric fields (CHEF) gel electrophoresis and analyzed with the Dice coefficient as described previously [15]. Isolates with greater than or equal to 90 % similarity are considered to be the same strain. Strain names are noted at the right and their corresponding MLST sequence types in parentheses. Additional file 2: Table S1. GenBank identifiers for *A. baumannii* genomes sequenced at JCVI in this study. Additional file 3: Table S2. Antibiotic resistance susceptibility profiles and predicted resistance mechanisms for *A. baumannii* genomes sequenced in this study. Additional file 4: Table S3. All *A. baumannii* isolates used in this study. Additional file 5:
**Text file of all PanOCT clusters.**
Additional file 6: Figure S2. Phylogenetic tree of the *A. baumannii* pan-genome. A dendrogram was constructed based on the mean of the pairwise BLASTP score ratios (BSRs) of core protein clusters that were present in 100 % of all 249 *A. baumannii* isolates constituting the pan-genome. The BSR tree was generated from the PanOCT-derived BSR distance matrix using the Interactive Tree of Life (iTOL). The five most abundant MLST sequence types with available genome sequence are illustrated by color highlights (see inset key). The 50 isolates sequenced in this study are noted with a *red bar* on the outside of the tree. Genomes chosen for sub-sampling of the pan-genome by hierarchical clustering are marked with a *gold bar* on the outside of the tree. Additional file 7: Figure S3.
*A. baumannii* pan-genome of ST 2 genomes. The pan-genome size (*left column*) and the number of novel genes discovered with the addition of each new genome (*right column*) were estimated for 111 ST 2 genomes using a pan-genome model based on the original Tettelin et al. model [42]. *Purple circles* are the median of each distribution (*gray circles*). Power law (*red lines*) and exponential (*blue lines*) regressions were plotted to determine α (open/closed status) and tg(θ), the average extrapolated number of strain-specific/novel genes, respectively. Additional file 8:
**Text file of cluster assembly composition.**
Additional file 9:
**Text file of the location and composition of fGRs.**
Additional file 10: Table S4. Chromosomally encoded antibiotic resistance genes found within fGIs and fGRs. Additional file 11: Table S5. Resistance island target sites. Additional file 12: Table S6.
**a** Distribution of RI-positive isolates among *A. baumannii* genomes analyzed. **b** Summary of major RI signatures identified among CC1, CC2, CC3, and ST 25 isolates. All RI signatures identified in (**c**) CC1, (**d**) CC2, (**e**) CC3, (**f**) ST25, and (**g**) other isolates. **h** A list of RI target genes showing total gene length detected when intact and having no RI insertion, or carrying a RI insertion with junction fragments. **i** Total gene length of the *comM* target gene detected in a collection of finished *A. baumannii* genomes. Additional file 13: Figure S4.
*A. baumannii* whole genome SNP tree. A whole genome SNP tree was constructed for 249 *A. baumannii* genomes and four *Acinetobacter* spp. genomes. Major clonal complexes (CC1, CC2, and CC3), ST 25, and US hospital isolates forming CC2 UH clades A-E are highlighted with a colored background (see key) [40]. A colored box following the strain name marks the 50 isolates sequenced in this study (*red*) and the finished public reference genomes (*blue*). The annotation table (*right*) summarizes (i) RI signatures and (ii) virulence factor diversity reported in this study. See main text for a more detailed description. Briefly, (i) RI insertions were examined at the following gene loci: *comM*, *pho*, *astA*, acetylT, acylCS, and a hypothetical protein (Additional file 11). Specific RI insertion types detected at individual insertion loci were reported. A colored cell in the RI section of the annotation table represents the presence of an RI feature for a given isolate. For example, AbaR3 and AbaR4 type RIs are found at *comM* in CC1 isolates, whereas AbGRI1 type RIs instead are detected at *comM* in CC2 isolates. “*P*” was used to indicate that the Tn*1548* RI was detected on a plasmid in three finished genomes instead of the acetylT locus located on the chromosome (this study) [79]. “*#*” RI was previously reported at the *astA* locus but not in this study [71]. “***”, “**”, and “*S*” represent extreme antibiotic resistance, strong resistance, and susceptible to antibiotics as determined in this study, respectively (Additional file 3). The *black triangle* indicates a branch node where the loss of AbGRI1 insertion at the *comM* locus is suspected. Previously reported Aba-type RIs are listed in black bold to the right of the annotation table. (ii) Virulence factor diversity was detected as specific gain or loss of gene clusters involved in type I pili assembly, siderophore production, or efflux. In general, a colored cell in the virulence section of the annotation table represents the detection of a genomic variation, which in most cases indicates the loss of a gene cluster in a given isolate; the only exception is for siderophore cluster 2 in which a colored cell represents the specific gain of the gene cluster in the isolate. For example, type I pilus cluster 1 (*csu*) was lost in two ST 10 isolates, and also a subset of ST 2 isolates including the entire group of nine UH clade B strains and also the multi-drug resistant strain MDR-ZJ06. In contrast, across all 249 isolates analyzed, siderophore cluster 2 was only detected and thus specifically gained in ATCC 17978, and also six isolates belonging to strain types ST 81 and ST 94. A SNP matrix file containing 150,000 genomic variants for SNP tree construction is provided as Additional file 14. Additional file 14:
**Text file of SNP variants identified across 150,000 genomic positions (ACICU reference coordinates) and 249** ***A. baumannii*** **isolates analyzed.** This file was compressed using tar and 7-Zip (a). Additional file 15: Figure S5. Novel composite IS26 RI inserted into the acylCS gene locus. ACICU was used as a reference to show that an 18 kb composite IS26 RI replaced the original 8 kb genomic region in RI-positive isolates. The composite RI contains two resistance gene cassettes. The oldest isolate was NIPH 1669 (1997), which only carried the 5′ fragment of the composite IS26 RI including one resistance gene cassette. Pairwise nucleotide identity shown in a *red* to *blue* (100 % identity) color scale. Key: ORFs (*arrows*); drug resistance genes located on RI (*green*); deleted genes (*gray*); immediate RI flanking genes acylCS and a predicted exporter (*dark orange*); other flanking genes (*orange*). Additional file 16: Figure S6. Novel genomic fragment inserted into the acetylT gene locus. AB307-0294 was used as a reference to show the RI and non-RI type insertions found at the acetylT locus. **a** A 7.8 kb non-RI fragment was detected juxtaposed to the acetylT locus across all ST 25 isolates analyzed. The annotated salicylate monooxygenase gene located on the genomic fragment could be involved in catechol production. **b** A Tn*1548* RI insertion was detected at the acetylT locus in other isolates (e.g., multi-drug resistance MDR-TJ isolate). Pairwise nucleotide identity shown in a *red* to *blue* (100 % identity) color scale. Key: open reading-frames (*thick arrows*); drug resistance genes located on RI (*green*); RI insertion target acetylT (*dark green*); RI flanking genes (*orange*). Additional file 17: Table S7. Virulence and fitness factors used in this study. Additional file 18: Figure S7. Diversity of virulence and fitness factors based on centroid-to-ortholog BSR. Genomic regions involved in assembly of type I pili, siderophore production, and efflux were highly variable and showed specific gain or loss of the entire gene clusters in isolates analyzed. In the heat map, the presence, absence, and low similarity of a protein ortholog compared with its centroid is shown in *yellow* (BSR = 1), *blue* (BSR = 0), and *gray*, respectively. The list of virulence genes analyzed and the BSR-derived heat map file are provided in Additional files 17 and 19, respectively. Additional file 19: 
**Excel file containing the distance matrix of centroid-ortholog pairs based on BSR.**
Additional file 20: Table S8. Diversity of type I pili gene clusters in isolates analyzed. Additional file 21: Figure S8. Loss of type I pili cluster 3 gene cluster. A complete loss of the type I pilus cluster 3 was observed in all ST 2 isolates (e.g., ACICU, Naval-78, NIPH 528) and additional strain types including ST 25 (e.g., Naval-18, NIPH 146), ST 79 (e.g., UH7907), ST 113, ST 215, and others. Pairwise nucleotide identity shown in a *red* to *blue* (100 % identity) color scale. Key: open reading-frames (*thick arrows*); type I pilus cluster 3 (*green*); genes immediately flanking deletion (*dark orange*); other flanking genes (*orange*). Additional file 22: Figure S9. Loss of siderophore cluster 1 gene cluster. A complete loss of siderophore cluster 1 was observed in six isolates of mixed strain types, but within short phylogenetic distance as shown on the whole genome SNP tree. Pairwise nucleotide identity shown in a *red* to *blue* (100 % identity) color scale. Key: open reading-frames (*thick arrows*); siderophore cluster 1 (*green*); genes immediately flanking deletion (*dark orange*); other flanking genes (*orange*). Additional file 23:
**Command line arguments used for running NCBI.** ***blastall*** ***, panoct.pl*** **,** ***paralog_matchtable.pl*** ***, and gene_order.pl*** **.**
Additional file 24: Figure S10. Genomic locations of RIs and virulence factors analyzed in this study. The ACICU genome was used as a reference backbone to show genomic features analyzed in this study. Note that not all features were detected in the ACICU genome. Additional file 25:
**The JCVI-annotated combined amino acid fasta file used as input to** ***panoct.pl***. This file was compressed using tar and 7-Zip (a). Additional file 26: 
**The combined genome attribute file required to run** ***panoct.pl***, **which contains contig identifiers, protein identifiers, coordinates of protein coding regions, protein annotation, and genome identifiers.** This file was compressed using tar and 7-Zip (a). Additional file 27:
**A minimal set of output files generated by** ***panoct.pl*** **and** ***gene_order.pl.*** Also included are look-up tables to cross reference JCVI internal genome identifiers with GenBank identifiers as well as to cross-reference JCVI internal locus names with GenBank locus names. Note that many more output files were generated, which are informative, but not required to interpret the results of pan-genome analysis, except the output file of paralog_matchtable.pl, which was too large and easily made from the provided files. This file was compressed using tar and 7-Zip (a).

## Abbreviations

aaRS: aminoacyl tRNA synthetase
acetylT: acetyltransferase
acylCS: acyl-CoA synthase
ATCC: American Type Culture Collection
bp: base pair
BSR: BLAST score ratio
BWA: Burrows-Wheeler Aligner
CARD: Comprehensive Antibiotic Resistance Database
CC: clonal complex
CDC: Centers for Disease Control and Prevention
cGC: core gene cluster
fGI: flexible genomic island
fGR: flexible genomic region
GI: genomic island
HQD: high-quality draft
IHQD: improved high-quality draft
IS: insertion sequence
JCVI: J. Craig Venter Institute
kb: kilobase
Mbp: megabase pair
MDR: multidrug-resistant
MLST: multilocus sequence typing
MRSN: Multidrug-resistant Organism and Surveillance Network
ORF: open reading frame
PCR: polymerase chain reaction
PFGE: pulsed-field gel electrophoresis
RGI: Resistance Gene Identifier
RI: resistance island
SNP: single nucleotide polymorphism
ST: sequence type
Tat: twin-arginine translocation
WRAIR: Walter Reed Army Institute of Research
