# Supplementary figures and images for: A novel method of consensus pan-chromosome assembly and large-scale comparative analysis reveal the highly flexible pan-genome of Acinetobacter baumannii
Source: Genome Biol. 2015 Jul 21;16(1):143. doi: 10.1186/s13059-015-0701-6 (PMC4507327; doi:10.1186/s13059-015-0701-6)

Figure S1

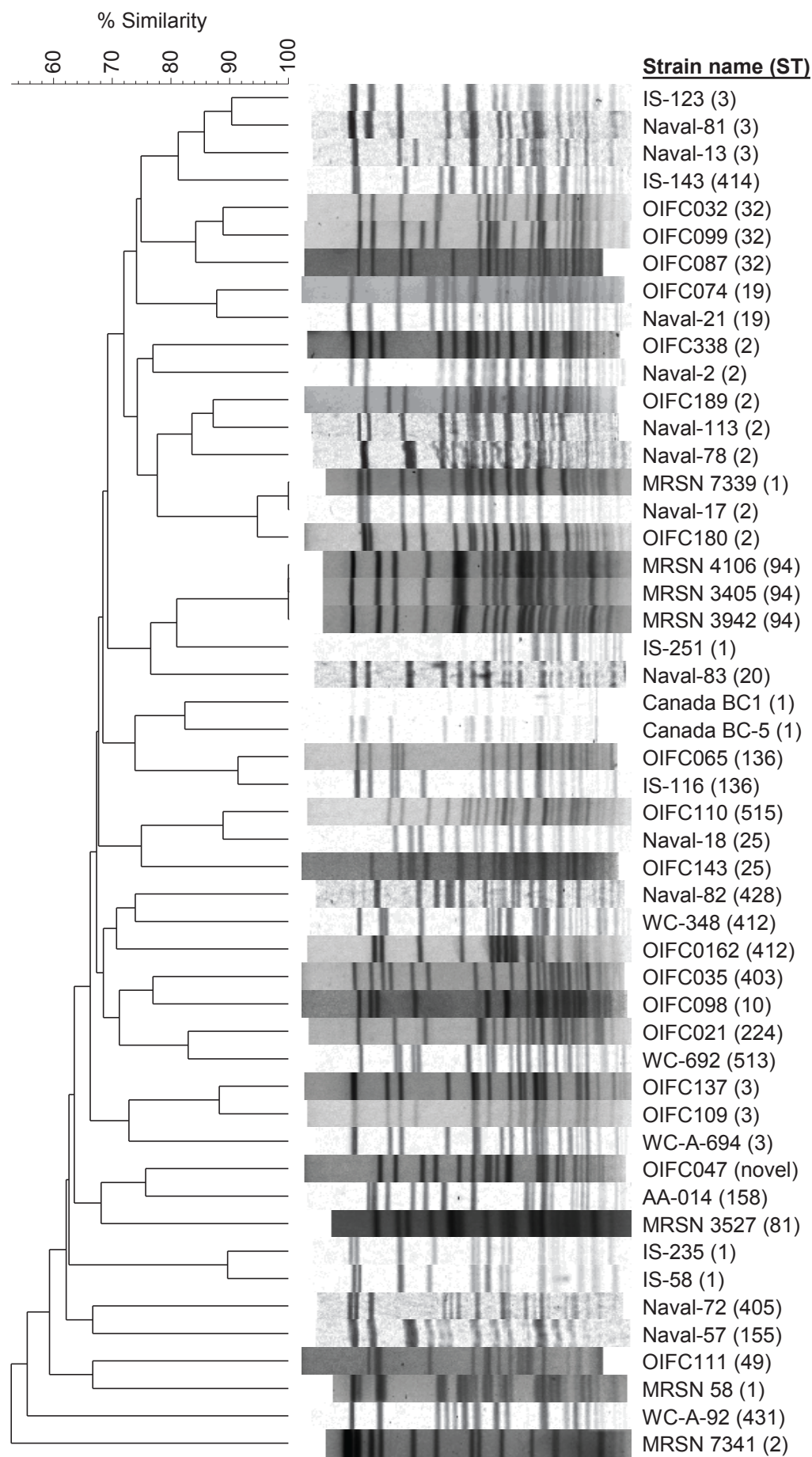

Supplement: Additional file 1: Figure S1. — Pulse field gel electrophoresis (PFGE) of A. baumannii isolates collected from the Military healthcare system. A dendrogram was produced based on the analysis of PFGE banding patterns. Genomic DNA was digested with ApaI, separated by clamped homogenous electric fields (CHEF) gel electrophoresis and analyzed with the Dice coefficient as described previously [15]. Isolates with greater than or equal to 90 % similarity are considered to be the same strain. Strain names are noted at the right and their corresponding MLST sequence types in parentheses. [file 13059_2015_701_MOESM1_ESM.pdf]

Figure S2

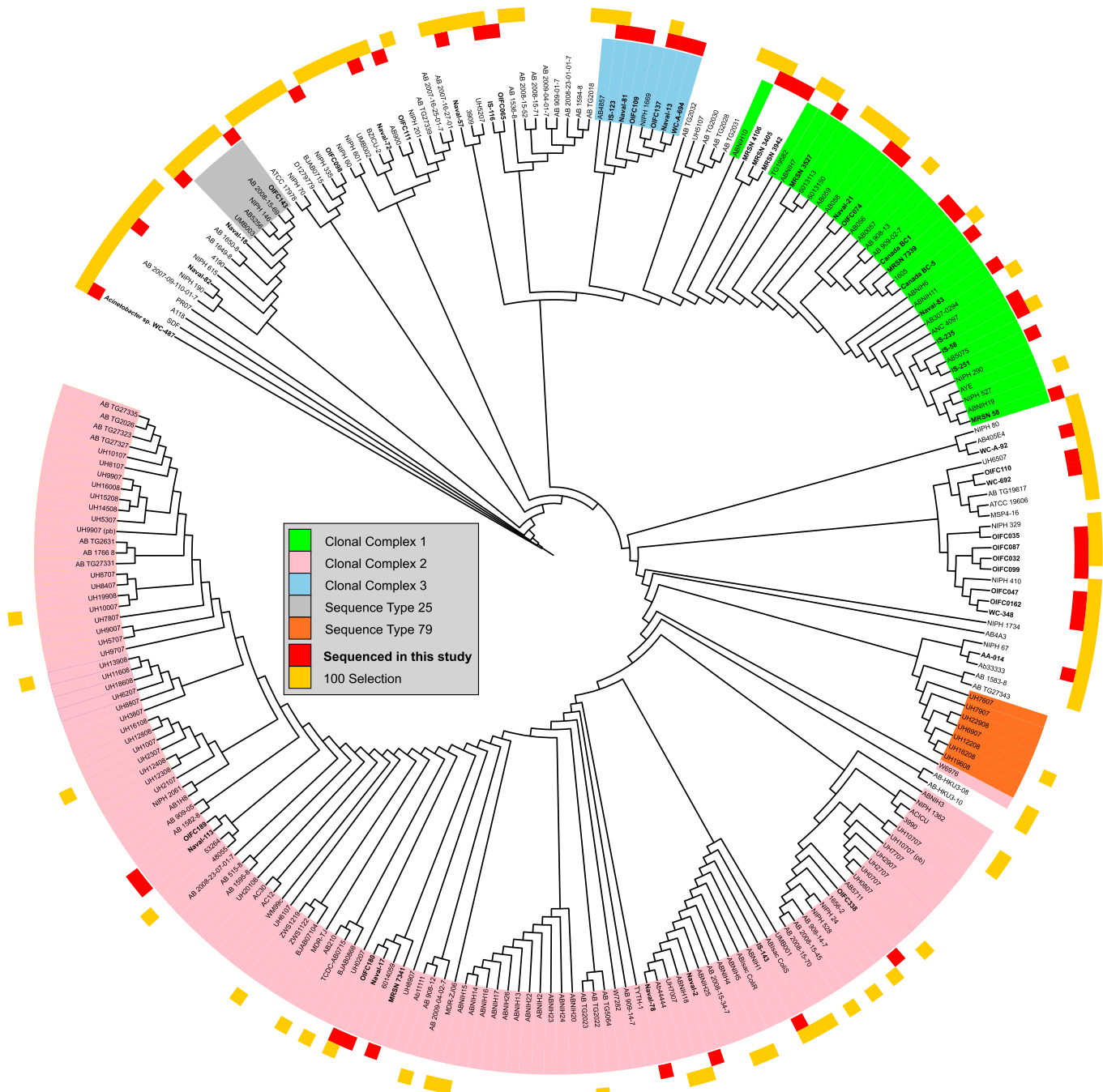

Supplement: Additional file 6: Figure S2. — Phylogenetic tree of the A. baumannii pan-genome. A dendrogram was constructed based on the mean of the pairwise BLASTP score ratios (BSRs) of core protein clusters that were present in 100 % of all 249 A. baumannii isolates constituting the pan-genome. The BSR tree was generated from the PanOCT-derived BSR distance matrix using the Interactive Tree of Life (iTOL). The five most abundant MLST sequence types with available genome sequence are illustrated by color highlights (see inset key). The 50 isolates sequenced in this study are noted with a red bar on the outside of the tree. Genomes chosen for sub-sampling of the pan-genome by hierarchical clustering are marked with a gold bar on the outside of the tree. [file 13059_2015_701_MOESM6_ESM.pdf]

Figure S3

### A. Pan-genome Size of CC2 111 genomes

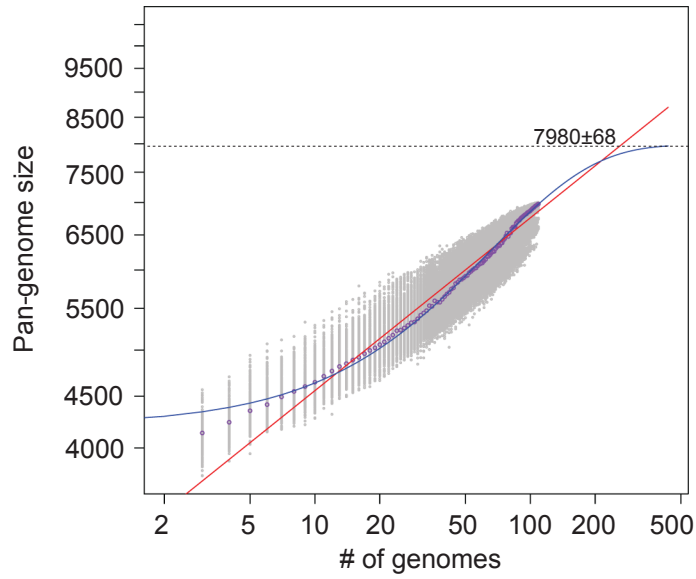

### B. Number of New Genes from CC2 111 genomes

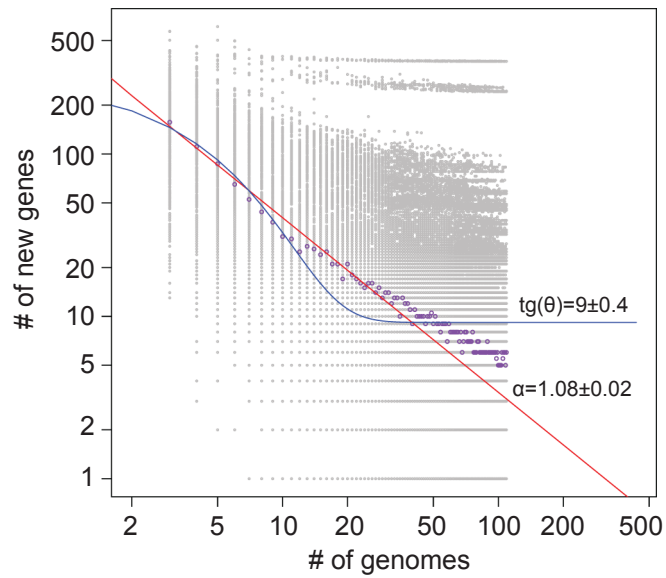

Supplement: Additional file 7: Figure S3. — A. baumannii pan-genome of ST 2 genomes. The pan-genome size (left column) and the number of novel genes discovered with the addition of each new genome (right column) were estimated for 111 ST 2 genomes using a pan-genome model based on the original Tettelin et al. model [42]. Purple circles are the median of each distribution (gray circles). Power law (red lines) and exponential (blue lines) regressions were plotted to determine α (open/closed status) and tg(θ), the average extrapolated number of strain-specific/novel genes, respectively. [file 13059_2015_701_MOESM7_ESM.pdf]

## Figure S4

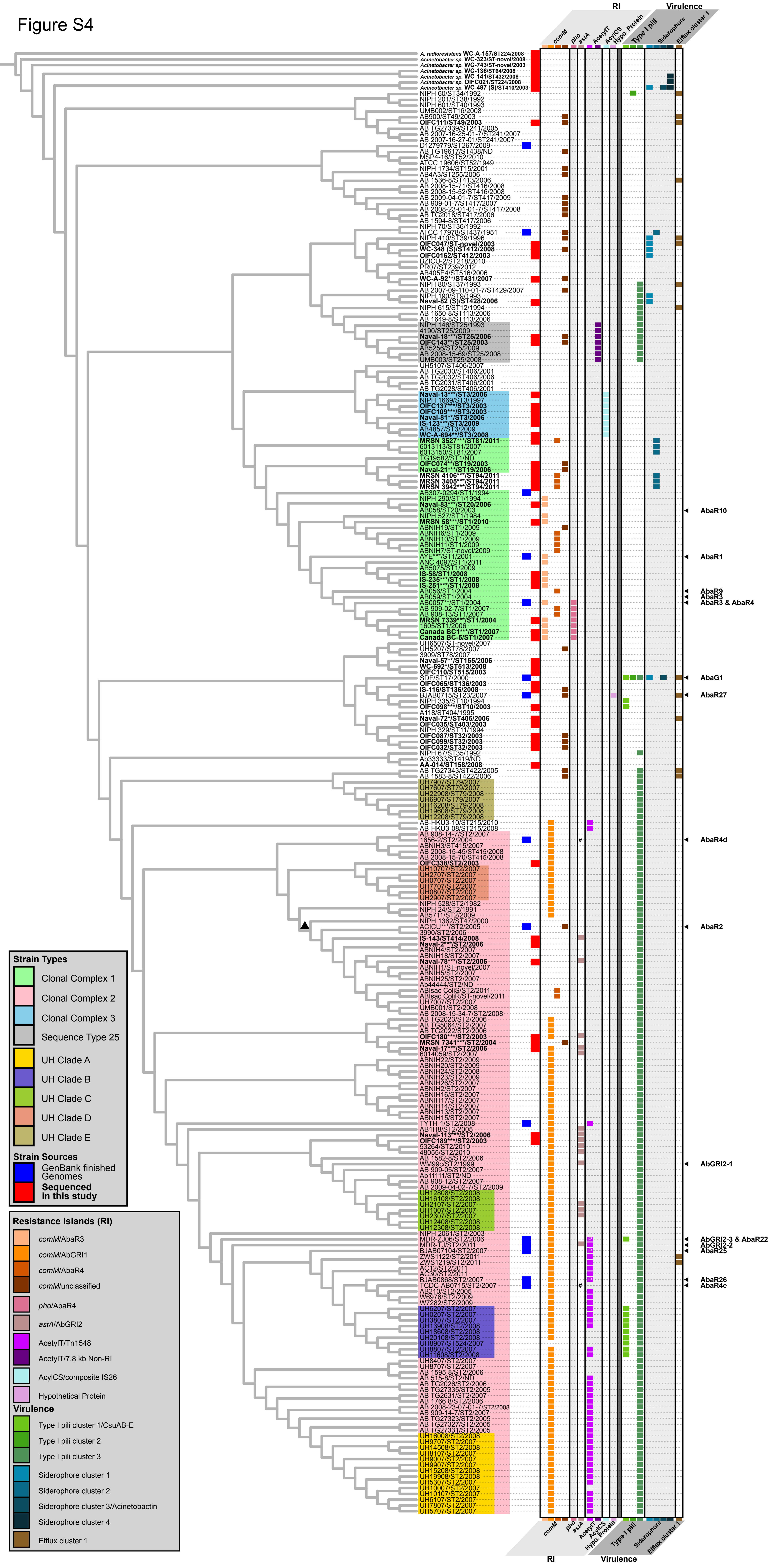

Supplement: Additional file 13: Figure S4. — A. baumannii whole genome SNP tree. A whole genome SNP tree was constructed for 249 A. baumannii genomes and four Acinetobacter spp. genomes. Major clonal complexes (CC1, CC2, and CC3), ST 25, and US hospital isolates forming CC2 UH clades A-E are highlighted with a colored background (see key) [40]. A colored box following the strain name marks the 50 isolates sequenced in this study (red) and the finished public reference genomes (blue). The annotation table (right) summarizes (i) RI signatures and (ii) virulence factor diversity reported in this study. See main text for a more detailed description. Briefly, (i) RI insertions were examined at the following gene loci: comM, pho, astA, acetylT, acylCS, and a hypothetical protein (Additional file 11). Specific RI insertion types detected at individual insertion loci were reported. A colored cell in the RI section of the annotation table represents the presence of an RI feature for a given isolate. For example, AbaR3 and AbaR4 type RIs are found at comM in CC1 isolates, whereas AbGRI1 type RIs instead are detected at comM in CC2 isolates. “P” was used to indicate that the Tn1548 RI was detected on a plasmid in three finished genomes instead of the acetylT locus located on the chromosome (this study) [79]. “#” RI was previously reported at the astA locus but not in this study [71]. “***”, “**”, and “S” represent extreme antibiotic resistance, strong resistance, and susceptible to antibiotics as determined in this study, respectively (Additional file 3). The black triangle indicates a branch node where the loss of AbGRI1 insertion at the comM locus is suspected. Previously reported Aba-type RIs are listed in black bold to the right of the annotation table. (ii) Virulence factor diversity was detected as specific gain or loss of gene clusters involved in type I pili assembly, siderophore production, or efflux. In general, a colored cell in the virulence section of the annotation table represents the detection of a genomi [file 13059_2015_701_MOESM13_ESM.pdf]

Figure S5

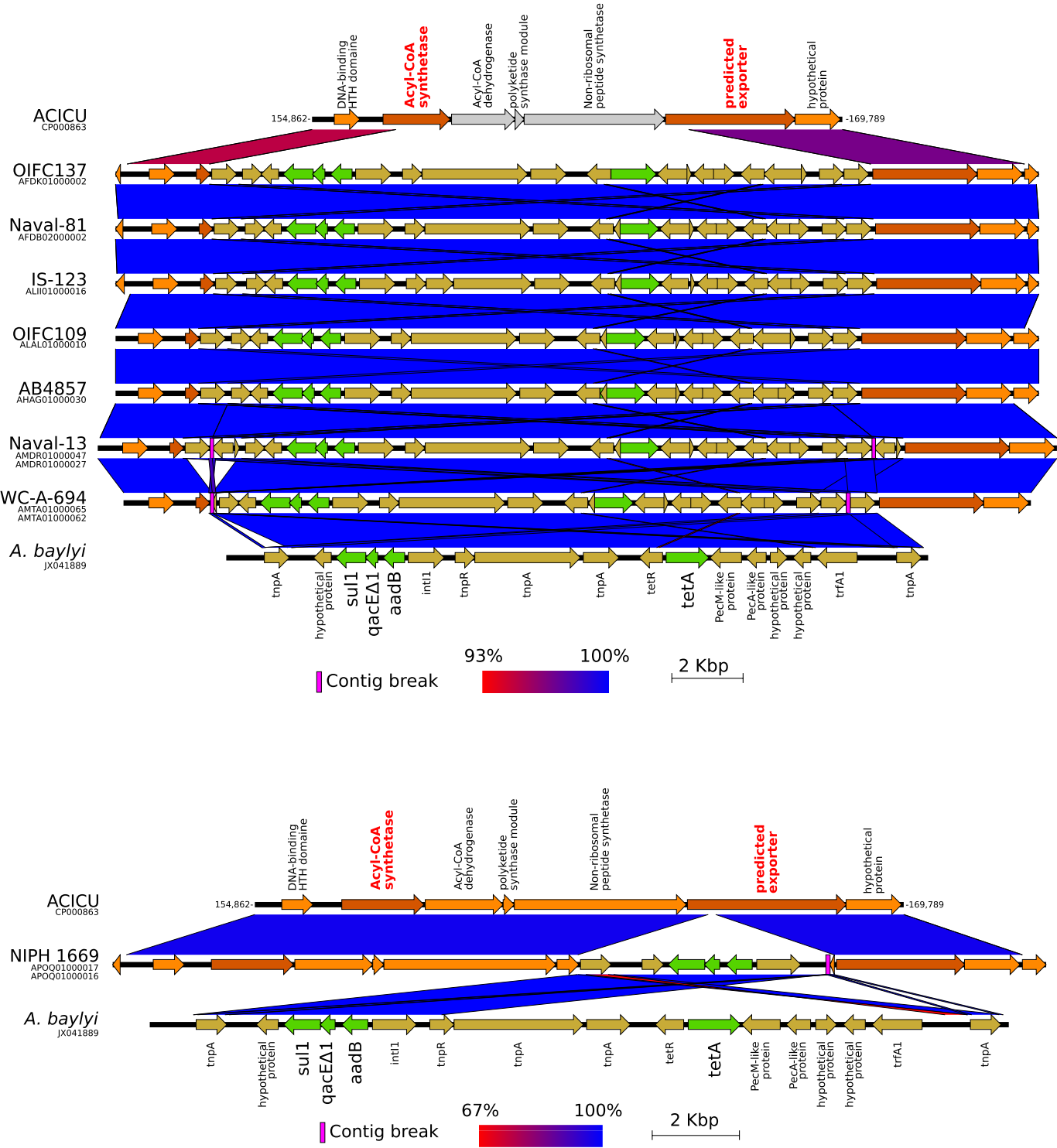

Supplement: Additional file 15: Figure S5. — Novel composite IS26 RI inserted into the acylCS gene locus. ACICU was used as a reference to show that an 18 kb composite IS26 RI replaced the original 8 kb genomic region in RI-positive isolates. The composite RI contains two resistance gene cassettes. The oldest isolate was NIPH 1669 (1997), which only carried the 5′ fragment of the composite IS26 RI including one resistance gene cassette. Pairwise nucleotide identity shown in a red to blue (100 % identity) color scale. Key: ORFs (arrows); drug resistance genes located on RI (green); deleted genes (gray); immediate RI flanking genes acylCS and a predicted exporter (dark orange); other flanking genes (orange). [file 13059_2015_701_MOESM15_ESM.pdf]

Figure S6

A. 7.8 kb non-RI insertion at the acetyIT locus

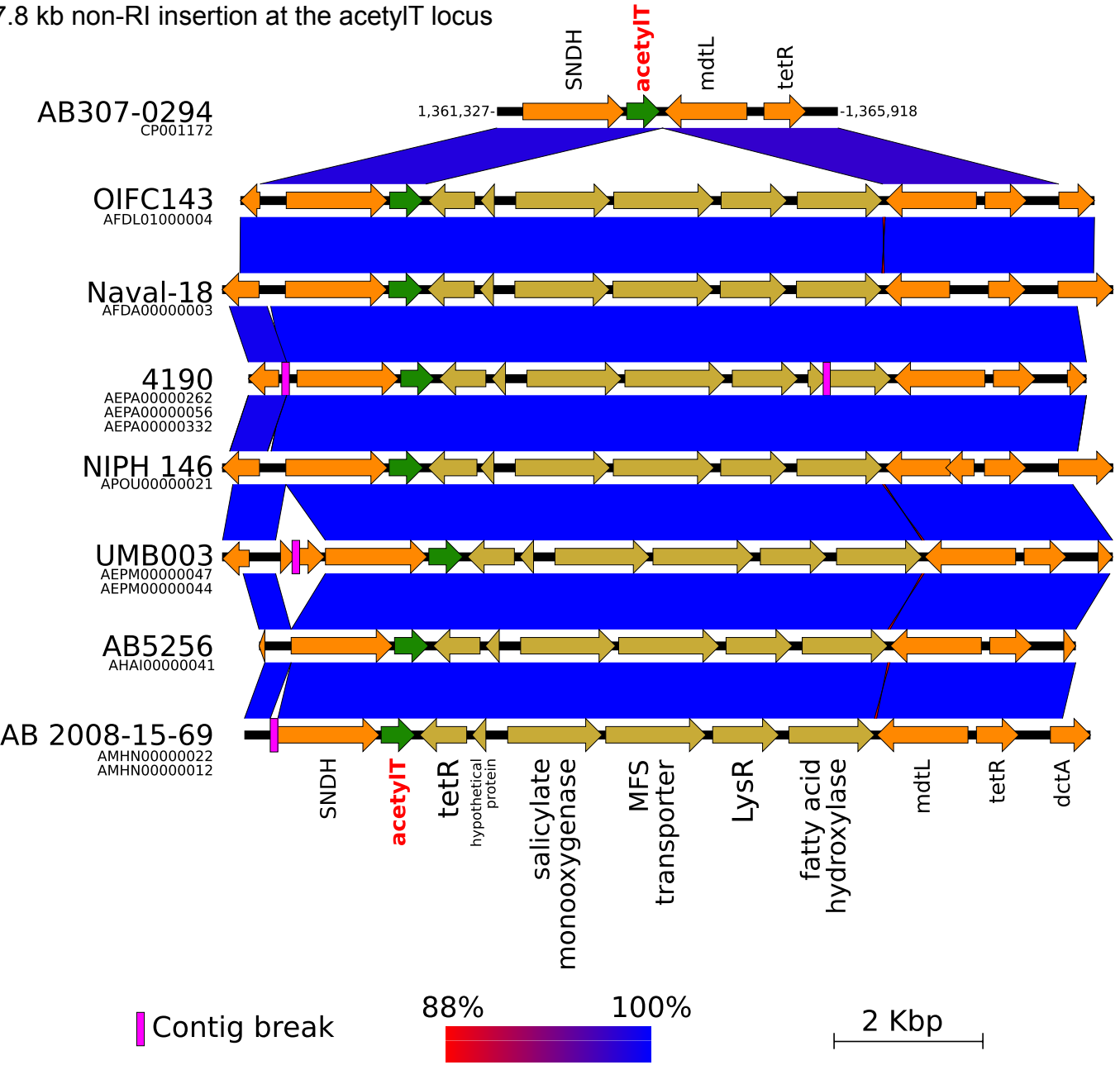

B. RI insertion at the acetyIT locus

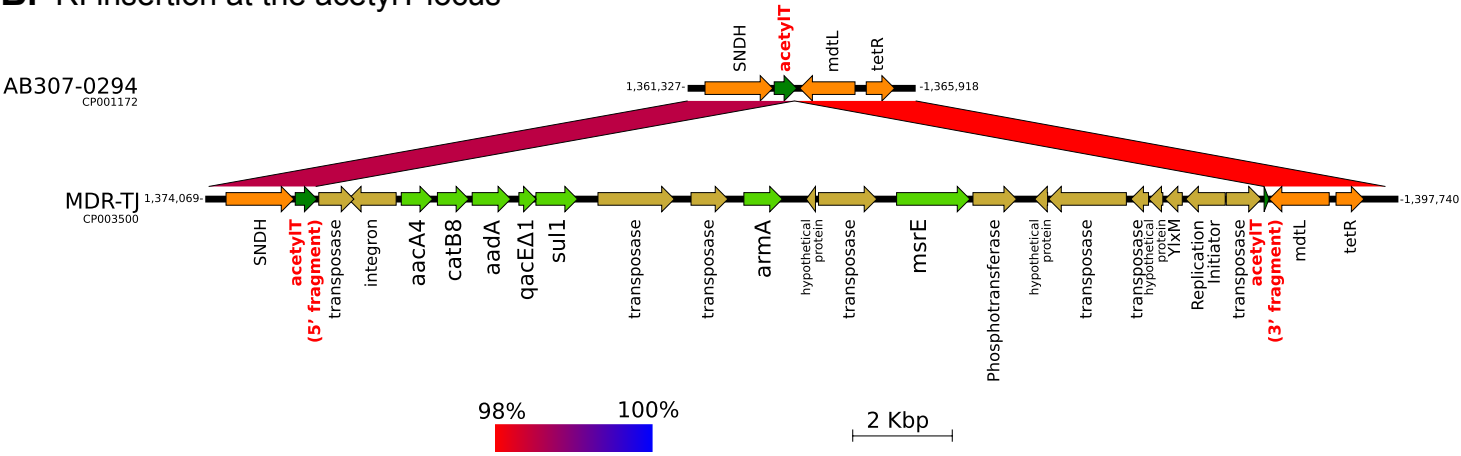

Supplement: Additional file 16: Figure S6. — Novel genomic fragment inserted into the acetylT gene locus. AB307-0294 was used as a reference to show the RI and non-RI type insertions found at the acetylT locus. a A 7.8 kb non-RI fragment was detected juxtaposed to the acetylT locus across all ST 25 isolates analyzed. The annotated salicylate monooxygenase gene located on the genomic fragment could be involved in catechol production. b A Tn1548 RI insertion was detected at the acetylT locus in other isolates (e.g., multi-drug resistance MDR-TJ isolate). Pairwise nucleotide identity shown in a red to blue (100 % identity) color scale. Key: open reading-frames (thick arrows); drug resistance genes located on RI (green); RI insertion target acetylT (dark green); RI flanking genes (orange). [file 13059_2015_701_MOESM16_ESM.pdf]

**Figure S7. Heat map of BSR distance derived from centroid-ortholog pairs**

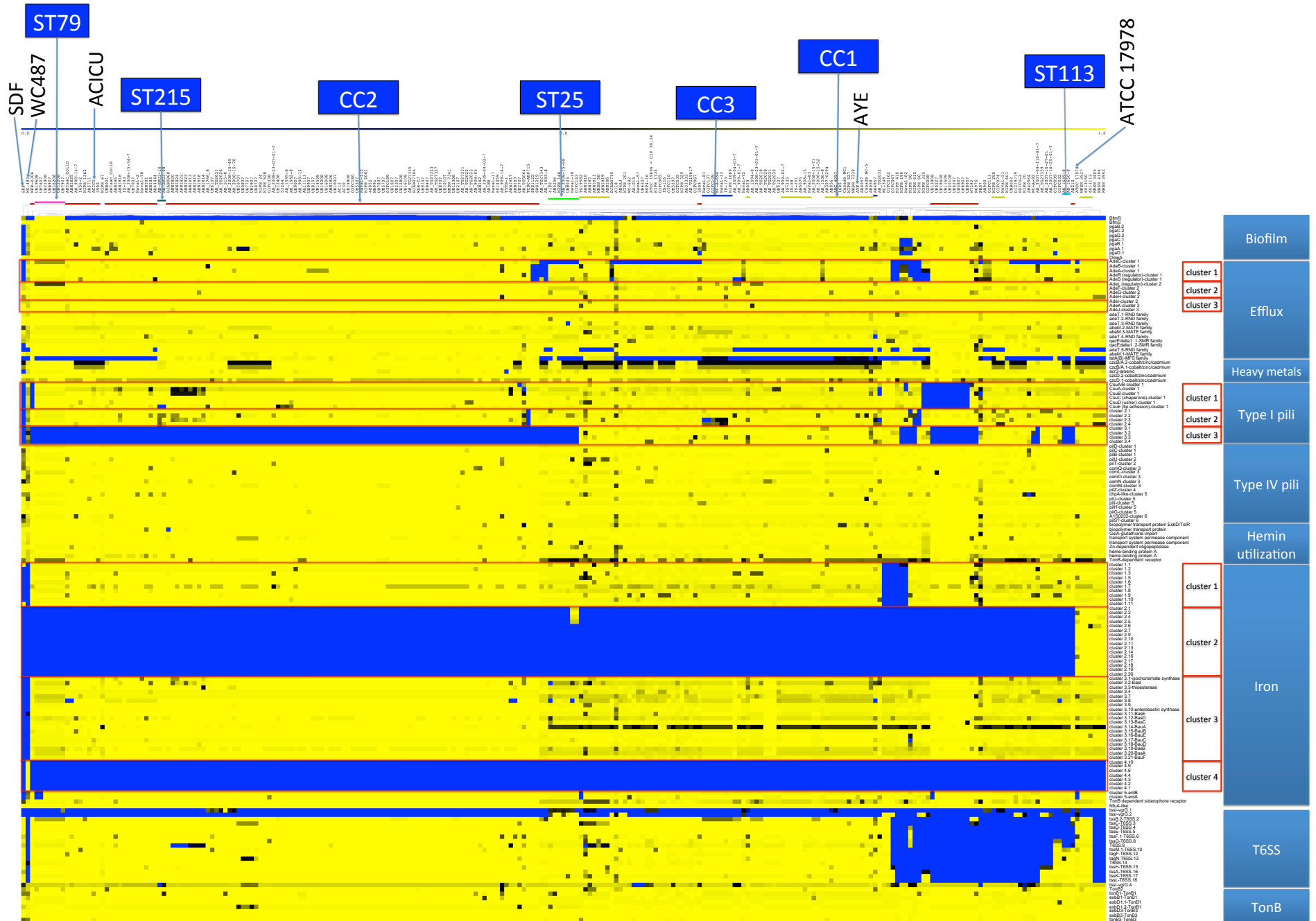

Supplement: Additional file 18: Figure S7. — Diversity of virulence and fitness factors based on centroid-to-ortholog BSR. Genomic regions involved in assembly of type I pili, siderophore production, and efflux were highly variable and showed specific gain or loss of the entire gene clusters in isolates analyzed. In the heat map, the presence, absence, and low similarity of a protein ortholog compared with its centroid is shown in yellow (BSR = 1), blue (BSR = 0), and gray, respectively. The list of virulence genes analyzed and the BSR-derived heat map file are provided in Additional files 17 and 19, respectively. [file 13059_2015_701_MOESM18_ESM.pdf]

Figure S8

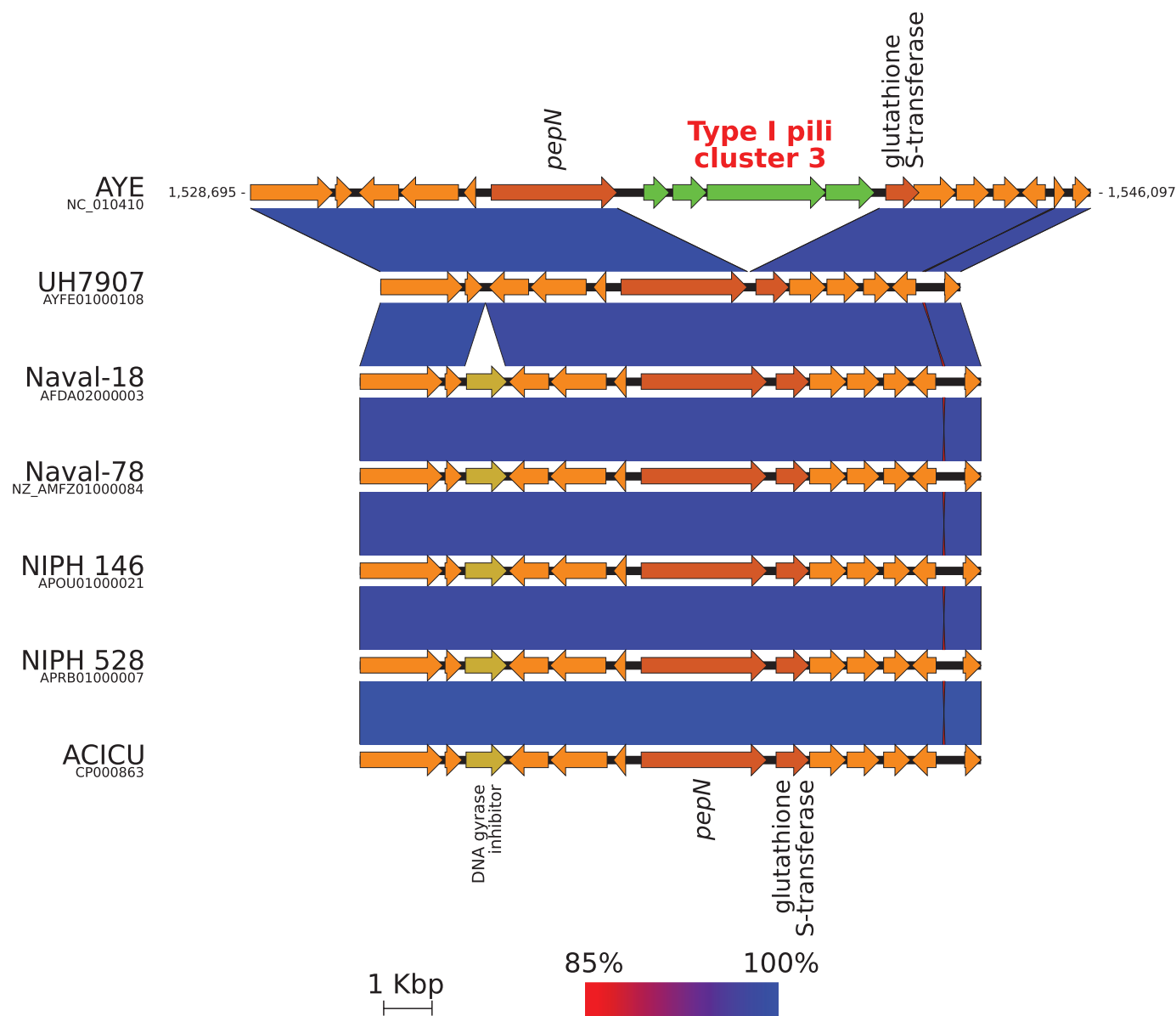

Supplement: Additional file 21: Figure S8. — Loss of type I pili cluster 3 gene cluster. A complete loss of the type I pilus cluster 3 was observed in all ST 2 isolates (e.g., ACICU, Naval-78, NIPH 528) and additional strain types including ST 25 (e.g., Naval-18, NIPH 146), ST 79 (e.g., UH7907), ST 113, ST 215, and others. Pairwise nucleotide identity shown in a red to blue (100 % identity) color scale. Key: open reading-frames (thick arrows); type I pilus cluster 3 (green); genes immediately flanking deletion (dark orange); other flanking genes (orange). [file 13059_2015_701_MOESM21_ESM.pdf]

Figure S9

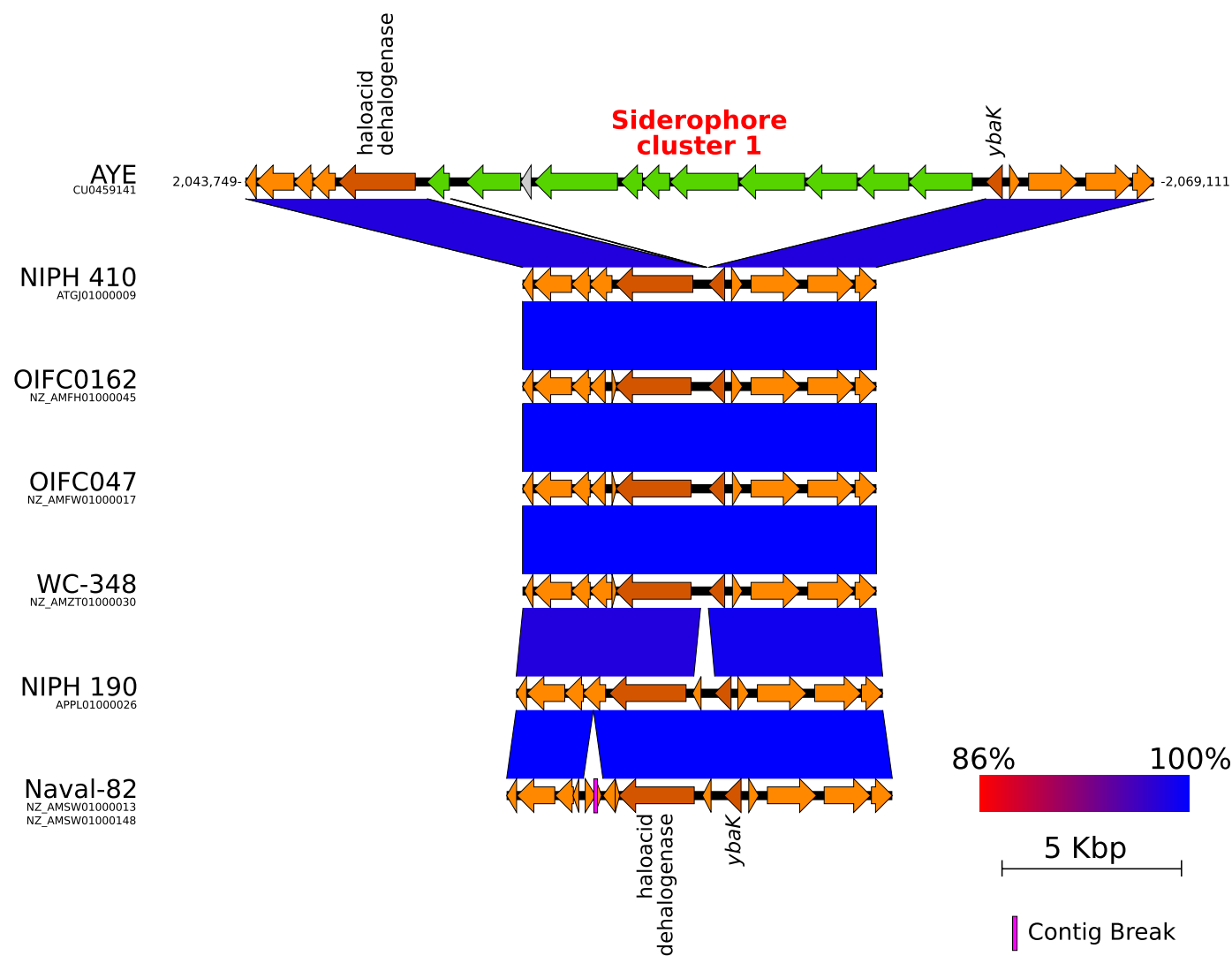

Supplement: Additional file 22: Figure S9. — Loss of siderophore cluster 1 gene cluster. A complete loss of siderophore cluster 1 was observed in six isolates of mixed strain types, but within short phylogenetic distance as shown on the whole genome SNP tree. Pairwise nucleotide identity shown in a red to blue (100 % identity) color scale. Key: open reading-frames (thick arrows); siderophore cluster 1 (green); genes immediately flanking deletion (dark orange); other flanking genes (orange). [file 13059_2015_701_MOESM22_ESM.pdf]

Figure S10

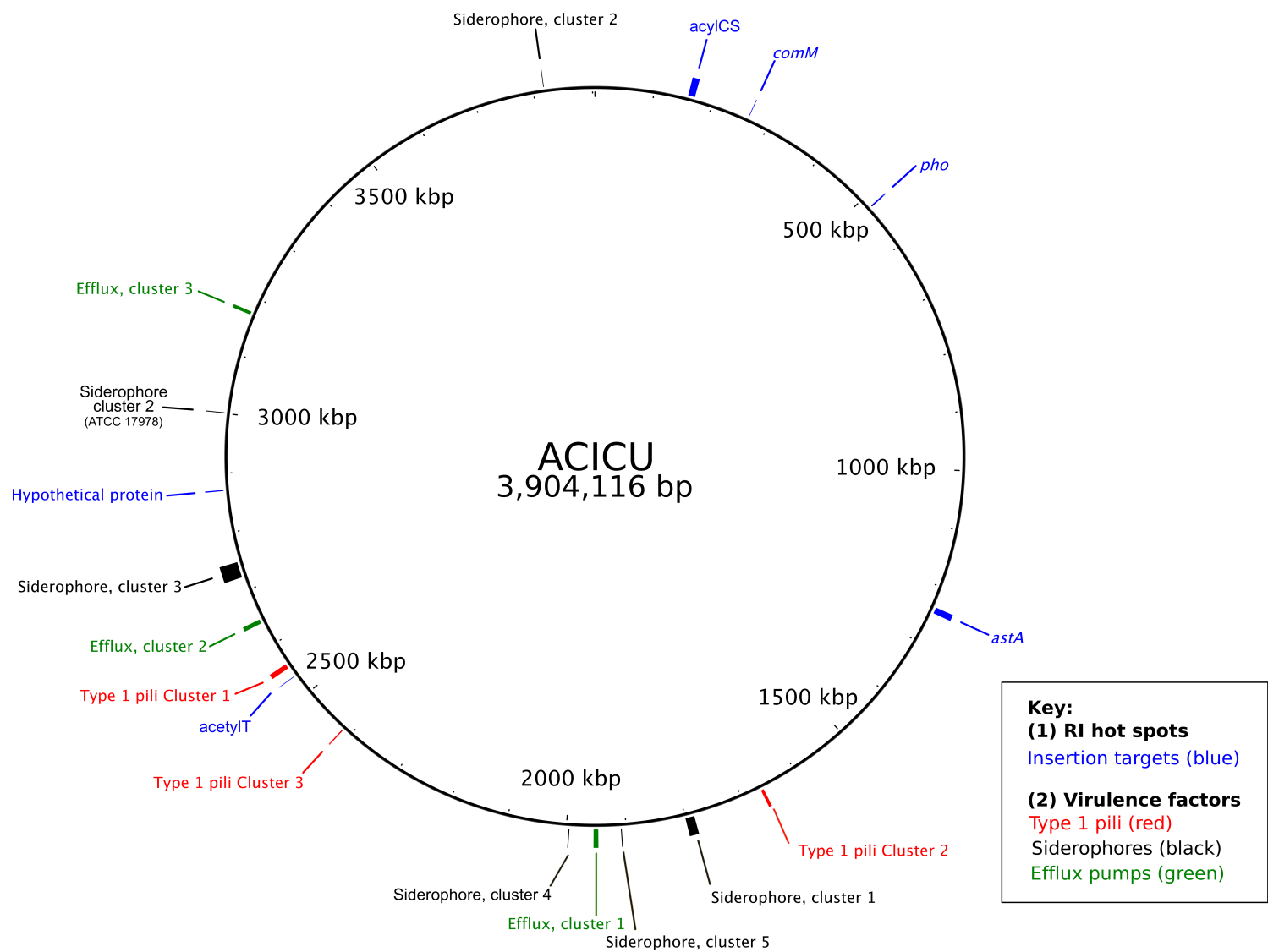

Supplement: Additional file 24: Figure S10. — Genomic locations of RIs and virulence factors analyzed in this study. The ACICU genome was used as a reference backbone to show genomic features analyzed in this study. Note that not all features were detected in the ACICU genome. [file 13059_2015_701_MOESM24_ESM.pdf]
