# Supplementary material for: A novel method of consensus pan-chromosome assembly and large-scale comparative analysis reveal the highly flexible pan-genome of Acinetobacter baumannii
Source: Genome Biol. 2015 Jul 21;16(1):143. doi: 10.1186/s13059-015-0701-6 (PMC4507327; doi:10.1186/s13059-015-0701-6)
Supplement: Additional file 2: Table S1. — GenBank identifiers for A. baumannii genomes sequenced at JCVI in this study. [file 13059_2015_701_MOESM2_ESM.pdf]

Table S1. GenBank Identifiers for *A. baumannii* Genomes Sequenced at JCVI in this Study

#	Strain	Bioproject	Biosample	WGS Master	SRA - 454[†]	SRA - Illumina[‡]
1)	OIFC137	PRJNA53377	SAMN02436470	AFDK00000000	SRX031275	SRX101596, SRX101800
2)	OIFC032	PRJNA53379	SAMN00114924	AFCZ00000000	SRX027592	N.A.
3)	OIFC109	PRJNA53383	SAMN02436409	ALAL00000000	SRX031272	SRX101598, SRX101793
4)	OIFC143	PRJNA53385	SAMN02436631	AFDL00000000	SRX031273	SRX101601, SRX101795
5)	OIFC189	PRJNA53387	SAMN00114925	AFDM00000000	SRX027593	SRX101602, SRX101794
6)	Canada BC-5	PRJNA53389	SAMN02436468	AFDN00000000	SRX027597	SRX101603, SRX101796
7)	Naval-17	PRJNA53391	SAMN02436551	AFDO00000000	SRX027591	SRX101604, SRX101797
8)	Naval-18	PRJNA53393	SAMN00114927	AFDA00000000	SRX027595	N.A.
9)	Naval-81	PRJNA53395	SAMN00114928	AFDB00000000	SRX027596	N.A.
10)	IS-123	PRJNA53397	SAMN02436485	ALII00000000	SRX032336	SRX101606, SRX101798
11)	OIFC074	PRJNA53401	SAMN00761203	AMDE00000000	N.A.	SRX110040
12)	OIFC098	PRJNA53403	SAMN00761228	AMDF00000000	N.A.	SRX110104
13)	OIFC180	PRJNA53405	SAMN00761237	AMDQ00000000	N.A.	SRX110113
14)	Naval-13	PRJNA53407	SAMN00761220	AMDR00000000	N.A.	SRX110096
15)	IS-235	PRJNA53409	SAMN00761221	AMEI00000000	N.A.	SRX110097
16)	IS-251	PRJNA53411	SAMN00761222	AMEJ00000000	N.A.	SRX110098
17)	OIFC0162	PRJNA53413	SAMN00761223	AMFH00000000	N.A.	SRX110099
18)	Naval-72	PRJNA53415	SAMN00761229	AMFI00000000	N.A.	SRX110105
19)	Naval-83	PRJNA53417	SAMN00761238	AMFK00000000	N.A.	SRX110114
20)	OIFC110	PRJNA53419	SAMN00761230	AMFL00000000	N.A.	SRX110106
21)	IS-143	PRJNA53421	SAMN00761239	AMGE00000000	N.A.	SRX110115
22)	IS-116	PRJNA53423	SAMN00761231	AMGF00000000	N.A.	SRX110107
23)	WC-692	PRJNA53425	SAMN00761214	AMGG00000000	N.A.	SRX110090
24)	IS-58	PRJNA53427	SAMN00761215	AMGH00000000	N.A.	SRX110091
25)	WC-487	PRJNA53433	SAMN00761233	AMZR00000000	N.A.	SRX110109
26)	WC-348	PRJNA53437	SAMN00761224	AMZT00000000	N.A.	SRX110100
27)	Naval-113	PRJNA53439	SAMN00761225	AMZU01000000	N.A.	SRX110101
28)	Naval-82	PRJNA53441	SAMN00761226	AMSW00000000	N.A.	SRX110102
29)	Naval-2	PRJNA53443	SAMN00761227	AMSX00000000	N.A.	SRX110103
30)	Naval-21	PRJNA53445	SAMN00761242	AMSY00000000	N.A.	SRX110118
31)	Canada BC1	PRJNA53447	SAMN00761234	AMSZ00000000	N.A.	SRX110110
32)	WC-A-694	PRJNA53449	SAMN00761243	AMTA00000000	N.A.	SRX110119
33)	OIFC035	PRJNA53451	SAMN00761235	AMTB00000000	N.A.	SRX110111
34)	Naval-57	PRJNA53453	SAMN00761216	AMFP00000000	N.A.	SRX110092
35)	OIFC087	PRJNA53459	SAMN00761219	AMFS00000000	N.A.	SRX110095
36)	OIFC099	PRJNA53461	SAMN00761236	AMFT00000000	N.A.	SRX110112
37)	WC-A-92	PRJNA53463	SAMN00761249	AMFU00000000	N.A.	SRX110125
38)	OIFC065	PRJNA53465	SAMN00761244	AMFV00000000	N.A.	SRX110120
39)	OIFC047	PRJNA53467	SAMN00761250	AMFW00000000	N.A.	SRX110126
40)	OIFC338	PRJNA53469	SAMN00761245	AMFX00000000	N.A.	SRX110121
41)	OIFC111	PRJNA53471	SAMN00761246	AMFY00000000	N.A.	SRX110122
42)	Naval-78	PRJNA53473	SAMN00761247	AMFZ00000000	N.A.	SRX110123
43)	AA-014	PRJNA53475	SAMN00761248	AMGA00000000	N.A.	SRX110124
44)	MRSN 3405	PRJNA223610	SAMN02906922	JPIA00000000	N.A.	SRR1945422
45)	MRSN 3527	PRJNA223611	SAMN02906923	JPHZ00000000	N.A.	SRR1945425
46)	MRSN 3942	PRJNA223612	SAMN02906926	JPHY00000000	N.A.	SRR1946597
47)	MRSN 4106	PRJNA223613	SAMN02906927	JPHX00000000	N.A.	SRR1945426
48)	MRSN 58	PRJNA223614	SAMN02906928	JPHW00000000	N.A.	SRR1945427
49)	MRSN 7339	PRJNA223615	SAMN02906929	JPHV00000000	N.A.	SRR1945428
50)	MRSN 7341	PRJNA223616	SAMN02906930	JPIB00000000	N.A.	SRR1945430

[†]GS FLX Titanium platform

[‡]Genome Analyzer II except for MRSN isolates, which were sequenced using HiSeq 2000

N.A. = Not Applicable
