# Supplementary material for: A novel method of consensus pan-chromosome assembly and large-scale comparative analysis reveal the highly flexible pan-genome of Acinetobacter baumannii
Source: Genome Biol. 2015 Jul 21;16(1):143. doi: 10.1186/s13059-015-0701-6 (PMC4507327; doi:10.1186/s13059-015-0701-6)
Supplement: Additional file 3: Table S2. — Antibiotic resistance susceptibility profiles and predicted resistance mechanisms for A. baumannii genomes sequenced in this study. [file 13059_2015_701_MOESM3_ESM.pdf]

Table S2. Antibiotic Resistance Susceptibility Profiles and Predicted Resistance Mechanisms for *A. baumannii* Genomes Sequenced in this Study

	Aminoglycoside				β-Lactam-Carbapenem											Quinolone				Sulfonamide			Tetracycline		Other		
					Carbapenem			Cephalosporin				Penicillin															
Strain	Amikacin	Gentamicin	Tobramycin	#Drug Resistance Genes	Ertapenem	Imipenem	Meropenem	Cefazolin (1st Gen)	Cefepime (4th Gen)	Ceftazidime (3rd Gen)	Ceftriaxone (3rd Gen)	Amoxicillin/Clavulanate	Ampicillin	Aztreonam	#Drug Resistance Genes	Ciprofloxacin (2nd Gen)	Levofloxacin (3rd Gen)	#Drug Resistance Genes	gyrA/parC QRDR Mutations§	Trimethoprim/Sulfamethoxazole	#Sulfonamide Resistance Genes	#Trimethoprim Resistance Genes	Tetracycline	#Drug Resistance Genes	Chloramphenicol	#Drug Resistance Genes	#Antibiotic Efflux Pump Genes
Naval-18	R	R	R	3 (3)	R	R	N.D.	R	R	R	R	R	R	R	6	R	R	1	RR	R	(1)	0	R	0	N.D.	2	24
OIFC137	R	R	R	5 (1)	R	S	N.D.	R	R	R	R	R	R	R	4	R	R	1	RR	R	1 (1)	0	R	2	N.D.	2	25
OIFC180	R	R	R	10	R	S	N.D.	R	R	R	R	R	R	R	6	R	R	1	RR	R	2	0	R	1	N.D.	2	22
Naval-13	R	R	R	4 (2)	R	S	N.D.	R	R	R	R	R	R	R	4	R	R	1	RR	R	1 (1)	0	R	2	N.D.	2	23
Naval-113	R	R	R	7	R	S	N.D.	R	R	R	R	R	R	R	6	R	R	1	RR	R	2	0	R	1	N.D.	2	21
BC-5	I	R	I	3	R	R	N.D.	R	R	R	R	R	R	R	8	R	R	1	RR	R	2	0	R	2	N.D.	3	24
Naval-83	R	R	R	3	N.D.	R	N.D.	R	R	R	R	R	R	R	6	R	R	1	RS	R	1	0	I	1	N.D.	2	22
Naval-21	S	R	R	3	R	S	N.D.	R	R	R	R	R	R	R	4	R	R	1	RR	R	1	0	R	3	N.D.	2	23
OIFC189	I	R	R	9	R	S	N.D.	R	I	R	R	R	R	R	6	R	R	1	RR	R	2	0	R	1	N.D.	2	24
Naval-78	R	R	R	4	N.D.	S	N.D.	R	I	R	R	R	R	R	4	R	R	1	RR	R	1	0	R	0	N.D.	2	23
IS-123	I	R	R	4	R	S	N.D.	R	S	R	R	R	R	R	4	R	R	1	RR	R	1	0	R	2	N.D.	2	27
OIFC098	S	R	R	5	R	S	N.D.	R	R	R	R	R	R	R	5	R	I	1	RS	R	1	0	R	1	N.D.	2	23
Naval-2	I	R	R	2	R	S	N.D.	R	R	R	R	R	R	R	4	R	R	1	RR	S	0	0	R	0	N.D.	2	22
Naval-17	S	R	S	7	R	S	N.D.	R	R	R	R	R	R	R	6	R	R	1	RR	R	1	0	R	1	N.D.	2	23
IS-235	R	R	R	8	N.D.	I	R	R	N.D.	R	R	N.D.	R	R	6	R	I	1	RR	R	1	0	S	1	R	2	23
IS-251	R	R	R	8	N.D.	R	R	N.D.	N.D.	R	R	N.D.	N.D.	R	6	R	R	1	RR	R	1	1	S	1	R	2	23
MRSN 3942	R	R	R	3 (2)	N.D.	R	N.D.	N.D.	R	R	R	N.D.	N.D.	R	6	R	R	1	RR	N.D.	2	0	R	1	N.D.	2	26
MRSN 4106	R	R	R	3 (1)	N.D.	R	N.D.	N.D.	R	R	R	N.D.	N.D.	R	6	R	R	1	RR	N.D.	2	0	R	1	N.D.	2	26
MRSN 7341	R	R	R	6	N.D.	R	N.D.	N.D.	R	R	R	N.D.	N.D.	R	7	R	R	1	RR	N.D.	2	0	R	1	N.D.	2	23
ACICU	R	S	R	3	N.D.	R	R	N.D.	R	R	N.D.	N.D.	R	R	5 (2)	R	N.D.	1	RS	R	1	0	N.D.	0	R	2	23
WC-694	S	R	R	4	R	S	N.D.	R	I	R	R	R	R	R	4	R	R	1	RR	N.D.	1	0	N.D.	2	N.D.	2	25
Naval-81	R	R	R	4	N.D.	S	N.D.	R	S	S	I	R	R	R	4	R	R	1	RR	R	1	0	R	2	N.D.	2	25
OIFC074	S	S	S	3	R	S	N.D.	R	R	R	R	R	R	R	4	R	I	1	RS	R	1	0	R	2	N.D.	3	25
Naval-57	S	R	I	5	R	R	N.D.	R	R	R	R	R	R	R	5	S	S	0	SS	R	1	0	S	0	N.D.	2	23
OIFC143	S	R	R	8 (2)	R	S	N.D.	R	R	R	R	R	R	R	5	R	S	1	RS	S	2 (1)	0	S	0	N.D.	2	24
MRSN 3405	R	R	R	3 (2)	N.D.	R	N.D.	N.D.	I	R	R	N.D.	N.D.	R	6	R	R	1	RR	N.D.	2	0	R	1	N.D.	2	26
MRSN 3527	R	R	R	2 (2)	N.D.	R	N.D.	N.D.	I	R	R	N.D.	N.D.	R	6	R	R	1	RR	N.D.	2	0	R	1	N.D.	2	25
AB0057	S	R	S	5	N.D.	R	R	R	R	N.D.	R	N.D.	R	R	8	R	N.D.	1	RR	R	2	0	I	2	N.D.	3	23
AYE	R	R	R	9	N.D.	S	S	N.D.	R	R	N.D.	R	R	R	6	R	N.D.	1	RR	R	4	1	N.D.	3	N.D.	3	25
MRSN 7339	S	R	S	6	N.D.	R	N.D.	N.D.	R	R	R	N.D.	N.D.	R	8	R	R	1	RR	N.D.	3	0	R	2	N.D.	3	23
WC-92	S	S	S	2	R	R	N.D.	R	R	R	R	R	R	R	4	S	S	0	SS	N.D.	0	0	N.D.	1	N.D.	2	21
MRSN 58*	S	R	R	4 (1)	N.D.	N.D.	R	N.D.	R	R	R	N.D.	N.D.	N.D.	6	R	I	1	RS	R	3	1	N.D.	1 (1)	N.D.	2	23
WC-743	S	S	S	0	R	S	N.D.	R	R	R	R	R	R	R	5	N.D.	S	0	SS	N.D.	0	0	N.D.	1	N.D.	2	29
WC-692	S	R	R	2	N.D.	S	S	N.D.	N.D.	R	N.D.	N.D.	N.D.	N.D.	5	S	S	0	SS	R	0	0	N.D.	1	N.D.	2	9
Naval-72	S	S	S	2	N.D.	S	N.D.	R	S	S	S	R	R	R	4	S	S	0	SS	S	0	0	S	0	N.D.	2	22
WC-141	S	S	S	1	N.D.	S	N.D.	N.D.	N.D.	S	N.D.	N.D.	N.D.	N.D.	4	S	S	0	SS	N.D.	0	0	N.D.	0	N.D.	2	21
WC-136	S	I	S	0	N.D.	S	S	N.D.	I	S	I	N.D.	N.D.	N.D.	3	N.D.	S	0	SS	S	0	0	N.D.	0	N.D.	2	23
WC-487	S	S	S	1	N.D.	S	N.D.	N.D.	N.D.	S	N.D.	N.D.	N.D.	N.D.	4	S	S	0	SS	N.D.	0	0	N.D.	0	N.D.	2	27
WC-348	S	S	S	4	N.D.	S	N.D.	N.D.	N.D.	S	N.D.	N.D.	N.D.	N.D.	4	S	S	0	SS	N.D.	1	0	N.D.	1	N.D.	2	28
Naval-82	S	S	S	2	N.D.	N.D.	N.D.	N.D.	N.D.	N.D.	N.D.	N.D.	N.D.	N.D.	4	S	S	0	SS	S	0	0	S	0	N.D.	2	24
WC-323	S	S	S	2	N.D.	S	S	N.D.	N.D.	S	N.D.	N.D.	N.D.	N.D.	5	S	S	0	SS	N.D.	1	0	N.D.	0	N.D.	2	24

Key

R Resistant
S Susceptible
I Intermediate
N.D. Not Determined

For each genome AST results are reported if available and the number of drug resistance genes found using CARD are reported for that antibiotic class. The number in () indicates the number of genes found on a plasmid and the number with no () indicates genes found only on the chromosome or contigs. §: RR, resistance-associated Ser84Leu mutation found in gyrA and parC; SS, susceptibility-associate alleles in both gryA and parC; RS, resistance-associate mutation found only in gyrA

Antimicrobial susceptibility tests (AST) were performed on all isolates at the WRAMC clinical laboratory using commercially available BD Phoenix NMIC/ID133 panel (Becton, Dickinson and Company, Franklin Lakes, NJ, USA). Susceptibility was determined according to Phoenix criteria and CLSI M-100-S-19, Vol29, No.3 2009. *For MRSN 58 ast were performed using commerically available Siemens MicroScan panel. Resistance data were published previously for AB0057 (Adams, 2008), ACICU (Longo, 2007) and AYE (Fournier, 2006; Poirel, 2003)

§: RR, resistance-associated Ser84Leu mutation found in *gyrA* and *parC*; SS, susceptibility-associate alleles in both *gryA* and *parC*; RS, resistance-associate mutation found only in *gyrA*
