# Supplementary material for: A novel method of consensus pan-chromosome assembly and large-scale comparative analysis reveal the highly flexible pan-genome of Acinetobacter baumannii
Source: Genome Biol. 2015 Jul 21;16(1):143. doi: 10.1186/s13059-015-0701-6 (PMC4507327; doi:10.1186/s13059-015-0701-6)
Supplement: Additional file 4: Table S3. — All A. baumannii isolates used in this study. [file 13059_2015_701_MOESM4_ESM.pdf]

Table S3. All *A. baumannii* Isolates Used in this Study

#	Strain	Accession	Source	Category	Year	ST	MLST allelic profile (cpn60:fusA:gltA:pyrG:recA:rplB:rpoB)
1	1605	AUWL00000000	Unknown	Global	2006	1	1-1-1-1-5-1-1
2	AB5075 (a.k.a. MRSN 959)	AHAH00000000	Internal	MRSN	2009	1	1-1-1-1-5-1-1
3	UH0207	AYGS00000000	Respiratory	US Hospital	2007	2	2-2-2-2-2-2-2
4	UH0707	AYGR00000000	Blood	US Hospital	2007	2	2-2-2-2-2-2-2
5	UH0807	AYGQ00000000	Respiratory	US Hospital	2007	2	2-2-2-2-2-2-2
6	UH10007	AYGO00000000	Respiratory	US Hospital	2007	2	2-2-2-2-2-2-2
7	UH1007	AYGP00000000	Wound	US Hospital	2007	2	2-2-2-2-2-2-2
8	UH10107	AYGN00000000	Respiratory	US Hospital	2007	2	2-2-2-2-2-2-2
9	UH10707	AYGM00000000	Respiratory	US Hospital	2007	2	2-2-2-2-2-2-2
10	UH10707	AYOI00000000	Respiratory	US Hospital	2007	2	2-2-2-2-2-2-2
11	UH11608	AYGL00000000	Urinary	US Hospital	2008	2	2-2-2-2-2-2-2
12	UH12208	AYGK00000000	Respiratory	US Hospital	2008	79	26-2-2-2-29-4-5
13	UH12308	AYGJ00000000	Urinary	US Hospital	2008	2	2-2-2-2-2-2-2
14	UH12408	AYGI00000000	Respiratory	US Hospital	2008	2	2-2-2-2-2-2-2
15	UH12808	AYGH00000000	Respiratory	US Hospital	2008	2	2-2-2-2-2-2-2
16	UH13908	AYGG00000000	Respiratory	US Hospital	2008	2	2-2-2-2-2-2-2
17	UH14508	AYGF00000000	Blood	US Hospital	2008	2	2-2-2-2-2-2-2
18	UH15208	AYGE00000000	Respiratory	US Hospital	2008	2	2-2-2-2-2-2-2
19	UH16008	AYGD00000000	Wound	US Hospital	2008	2	2-2-2-2-2-2-2
20	UH16108	AYGC00000000	Respiratory	US Hospital	2008	2	2-2-2-2-2-2-2
21	UH16208	AYGB00000000	Respiratory	US Hospital	2008	79	26-2-2-2-29-4-5
22	UH18608	AYGA00000000	Respiratory	US Hospital	2008	2	2-2-2-2-2-2-2
23	UH19608	AYFZ00000000	Urinary	US Hospital	2008	79	26-2-2-2-29-4-5
24	UH19908	AYFY00000000	Respiratory	US Hospital	2008	2	2-2-2-2-2-2-2
25	UH20108	AYFX00000000	Urinary	US Hospital	2008	2	2-2-2-2-2-2-2
26	UH2107	AYFW00000000	Respiratory	US Hospital	2007	2	2-2-2-2-2-2-2
27	UH22908	AYFV00000000	Wound	US Hospital	2008	79	26-2-2-2-29-4-5
28	UH2307	AYFU00000000	Respiratory	US Hospital	2007	2	2-2-2-2-2-2-2
29	UH2707	AYFT00000000	Respiratory	US Hospital	2007	2	2-2-2-2-2-2-2
30	UH2907	AYFS00000000	Respiratory	US Hospital	2007	2	2-2-2-2-2-2-2
31	UH3807	AYFR00000000	Respiratory	US Hospital	2007	2	2-2-2-2-2-2-2
32	UH5107	AYFQ00000000	Respiratory	US Hospital	2007	406	1-1-1-2-65-1-5
33	UH5207	AYFP00000000	Urinary	US Hospital	2007	78	25-3-6-2-28-1-29
34	UH5307	AYFO00000000	Respiratory	US Hospital	2007	2	2-2-2-2-2-2-2
35	UH5707	AYFN00000000	Wound	US Hospital	2007	2	2-2-2-2-2-2-2
36	UH6107	AYFM00000000	Respiratory	US Hospital	2007	2	2-2-2-2-2-2-2
37	UH6207	AYFL00000000	Respiratory	US Hospital	2007	2	2-2-2-2-2-2-2
38	UH6507	AYFK00000000	Urinary	US Hospital	2007	novel	5-2-NEW-2-2-2-5
39	UH6907	AYFJ00000000	Respiratory	US Hospital	2007	79	26-2-2-2-29-4-5
40	UH7007	AYFI00000000	Urinary	US Hospital	2007	2	2-2-2-2-2-2-2
41	UH7607	AYFH00000000	Urinary	US Hospital	2007	79	26-2-2-2-29-4-5
42	UH7707	AYFG00000000	Respiratory	US Hospital	2007	2	2-2-2-2-2-2-2
43	UH7807	AYFF00000000	Respiratory	US Hospital	2007	2	2-2-2-2-2-2-2
44	UH7907	AYFE00000000	Blood	US Hospital	2007	79	26-2-2-2-29-4-5
45	UH8107	AYFD00000000	Respiratory	US Hospital	2007	2	2-2-2-2-2-2-2
46	UH8407	AYFC00000000	Blood	US Hospital	2007	2	2-2-2-2-2-2-2
47	UH8707	AYFB00000000	Misc.	US Hospital	2007	2	2-2-2-2-2-2-2
48	UH8807	AYFA00000000	Respiratory	US Hospital	2007	2	2-2-2-2-2-2-2
49	UH8907	AYEZ00000000	Blood	US Hospital	2007	524	2-97-2-2-2-2-2
50	UH9007	AYEY00000000	Blood	US Hospital	2007	2	2-2-2-2-2-2-2
51	UH9707	AYEX00000000	Urinary	US Hospital	2007	2	2-2-2-2-2-2-2
52	UH9907	AYEW00000000	Respiratory	US Hospital	2007	2	2-2-2-2-2-2-2
53	UH9907	AYOH00000000	Respiratory	US Hospital	2007	2	2-2-2-2-2-2-2
54	OIFC137	AFDK00000000	Urinary	WRAIR	2003	3	3-3-2-2-3-1-3
55	OIFC032	AFCZ00000000	Wound	WRAIR	2003	32	1-1-2-2-3-4-4
56	OIFC109	ALAL00000000	Wound	WRAIR	2003	3	3-3-2-2-3-1-3
57	OIFC143	AFDL00000000	Wound	WRAIR	2003	25	3-3-2-4-7-2-4
58	OIFC189	AFDM00000000	Wound	WRAIR	2003	2	2-2-2-2-2-2-2
59	Canada BC-5	AFDN00000000	Misc.	WRAIR	2007	1	1-1-1-1-5-1-1
60	Naval-17	AFDO00000000	Wound	WRAIR	2006	2	2-2-2-2-2-2-2
61	Naval-18	AFDA00000000	Wound	WRAIR	2006	25	3-3-2-4-7-2-4
62	Naval-81	AFDB00000000	Blood	WRAIR	2006	3	3-3-2-2-3-1-3
63	IS-123	ALII00000000	Wound	WRAIR	2009	3	3-3-2-2-3-1-3
64	OIFC074	AMDE00000000	Misc.	WRAIR	2003	19	1-2-1-1-5-1-1
65	OIFC098	AMDF00000000	Misc.	WRAIR	2003	10	1-3-2-1-4-4-4
66	OIFC180	AMDQ00000000	Misc.	WRAIR	2003	2	2-2-2-2-2-2-2
67	Naval-13	AMDR00000000	Wound	WRAIR	2006	3	3-3-2-2-3-1-3
68	IS-235	AMEI00000000	Blood	WRAIR	2008	1	1-1-1-1-5-1-1
69	IS-251	AMEJ00000000	Respiratory	WRAIR	2008	1	1-1-1-1-5-1-1
70	OIFC0162	AMFH00000000	Respiratory	WRAIR	2003	412	1-52-2-2-67-4-5
71	Naval-72	AMFI00000000	Wound	WRAIR	2006	405	5-3-16-4-29-1-60
72	Naval-83	AMFK00000000	Wound	WRAIR	2006	20	3-1-1-1-5-1-1
73	OIFC110	AMFL00000000	Misc.	WRAIR	2003	515	56-3-2-2-9-4-14
74	IS-143	AMGE00000000	Wound	WRAIR	2008	414	2-2-2-2-2-37-2
75	IS-116	AMGF00000000	Wound	WRAIR	2008	136	3-2-19-25-5-2-5
76	WC-692	AMGG00000000	Skin	WRAIR	2008	513	56-3-55-2-9-4-14
77	IS-58	AMGH00000000	Respiratory	WRAIR	2008	1	1-1-1-1-5-1-1
78	WC-487	AMZR00000000	Skin	WRAIR	2008	410	20-26-26-14-26-16-23
79	WC-348	AMZT00000000	Skin	WRAIR	2008	412	1-52-2-2-67-4-5
80	Naval-113	AMZU00000000	Wound	WRAIR	2006	2	2-2-2-2-2-2-2
81	Naval-82	AMSW00000000	Blood	WRAIR	2006	428	3-1-2-3-6-1-16
82	Naval-2	AMSX00000000	Blood	WRAIR	2006	2	2-2-2-2-2-2-2
83	Naval-21	AMSY00000000	Wound	WRAIR	2006	19	1-2-1-1-5-1-1
84	Canada BC1	AMSZ00000000	Misc.	WRAIR	2007	1	1-1-1-1-5-1-1
85	WC-A-694	AMTA00000000	Misc.	WRAIR	2008	3	3-3-2-2-3-1-3
86	OIFC035	AMTB00000000	Wound	WRAIR	2003	403	3-2-6-1-3-4-56
87	Naval-57	AMFP00000000	Wound	WRAIR	2006	155	3-2-2-2-44-4-4

#	Strain	Accession	Source	Category	Year	ST	MLST allelic profile (cpn60:fusA:gltA:pyrG:recA:rplB:rpoB)
88	OIFC087	AMFS00000000	Misc.	WRAIR	2003	32	1-1-2-2-3-4-4
89	OIFC099	AMFT00000000	Misc.	WRAIR	2003	32	1-1-2-2-3-4-4
90	WC-A-92	AMFU00000000	Misc.	WRAIR	2007	431	1-4-2-1-70-1-2
91	OIFC065	AMFV00000000	Wound	WRAIR	2003	136	3-2-19-25-5-2-5
92	OIFC047	AMFW00000000	Misc.	WRAIR	2003	novel	1-75-2-2-67-1-2
93	OIFC338	AMFX00000000	Misc.	WRAIR	2003	2	2-2-2-2-2-2-2
94	OIFC111	AMFY00000000	Misc.	WRAIR	2003	49	3-3-6-2-3-1-5
95	Naval-78	AMFZ00000000	Wound	WRAIR	2006	2	2-2-2-2-2-2-2
96	AA-014	AMGA00000000	Wound	WRAIR	2008	158	41-42-13-1-5-4-14
97	MRSN 3405	JPIA00000000	Wound	MRSN	2011	94	1-2-2-1-5-1-1
98	MRSN 3527	JPHZ00000000	Wound	MRSN	2011	81	1-1-1-1-5-1-2
99	MRSN 3942	JPHY00000000	Wound	MRSN	2011	94	1-2-2-1-5-1-1
100	MRSN 4106	JPHX00000000	Wound	MRSN	2011	94	1-2-2-1-5-1-1
101	MRSN 58	JPHW00000000	Wound	MRSN	2010	1	1-1-1-1-5-1-1
102	MRSN 7339	JPHV00000000	Wound	MRSN	2004	1	1-1-1-1-5-1-1
103	MRSN 7341	JPIB00000000	Respiratory	MRSN	2004	2	2-2-2-2-2-2-2
104	ATCC 17978	CP000521.1	Misc.	Global	1951	437	3-2-2-2-30-4-28
105	AYE	CU459141.1	Urinary	Global	2001	1	1-1-1-1-5-1-1
106	SDF	CU468230.2	Misc.	Global	2000	17	3-29-30-1-9-1-4
107	ACICU	CP000863.1	Internal	Global	2005	2	2-2-2-2-2-2-2
108	AB307-0294	CP001172.1	Blood	Global	1994	1	1-1-1-1-5-1-1
109	AB0057	CP001182.1	Blood	WRAIR	2004	1	1-1-1-1-5-1-1
110	1656-2	CP001921.1	Respiratory	Global	2004-2005	2	2-2-2-2-2-2-2
111	TCDC-AB0715	CP002522.1	Misc.	Global	2007-2009	2	2-2-2-2-2-2-2
112	MDR-ZJ06	CP001937.1	Blood	Global	2006	2	2-2-2-2-2-2-2
113	TYTH-1	CP003856.1	Blood	Global	2008	2	2-2-2-2-2-2-2
114	MDR-TJ	CP003500.1	Misc.	Global	before 2011	2	2-2-2-2-2-2-2
115	D1279779	CP003967.1	Blood	Global	2009	267	12-37-2-2-3-2-14
116	BJAB07104	CP003846.1	Blood	Global	5/2007-4/2008	2	2-2-2-2-2-2-2
117	BJAB0715	CP003847.1	Misc.	Global	5/2007-4/2008	23	1-3-10-1-4-4-4
118	BJAB0868	CP003849.1	Internal	Global	5/2007-4/2008	2	2-2-2-2-2-2-2
119	September 13, 1910	AEOZ00000000	Misc.	Global	2007	2	2-2-2-2-2-2-2
120	December 3, 1910	AEOY00000000	Misc.	Global	2006	2	2-2-2-2-2-2-2
121	June 21, 1911	AEPA00000000	Misc.	Global	2009	25	3-3-2-4-7-2-4
122	6013113	ACYR00000000	Skin	Global	2007	81	1-1-1-1-5-1-2
123	6013150	ACYQ00000000	Skin	Global	2007	81	1-1-1-1-5-1-2
124	6014059	ACYS00000000	Skin	Global	2007	2	2-2-2-2-2-2-2
125	A118	AEOW00000000	Blood	Global	1995	404	3-49-61-2-5-2-4
126	AB056	ADGZ00000000	Blood	WRAIR	2004	1	1-1-1-2-65-1-5
127	AB058	ADHA00000000	Blood	WRAIR	2003	20	1-1-1-2-65-1-5
128	AB_TG2030	AMIJ00000000	Respiratory	Global	2001	406	1-1-1-2-65-1-5
129	AB_TG2031	AMIK00000000	Respiratory	Global	2001	406	1-1-1-2-65-1-5
130	AB_TG2032	AMIL00000000	Respiratory	Global	2006	406	1-1-1-2-65-1-5
131	AB_TG2631	AMIM00000000	Blood	Global	2007	2	2-2-2-2-2-2-2
132	AB_TG27323	AMIN00000000	Unknown	Global	2005	2	2-2-2-2-2-2-2
133	AB_TG27327	AMIO00000000	Wound	Global	2005	2	2-2-2-2-2-2-2
134	AB_TG27331	AMIP00000000	Respiratory	Global	2005	2	2-2-2-2-2-2-2
135	AB_TG27335	AMIQ00000000	Unknown	Global	2005	2	2-2-2-2-2-2-2
136	AB_TG27339	AMIR00000000	Respiratory	Global	2005	241	40-3-15-2-40-4-4
137	AB_TG27343	AMIS00000000	Wound	Global	2005	422	26-72-2-2-29-4-5
138	AB059	ADHB00000000	Blood	WRAIR	2004	1	1-1-1-1-5-1-1
139	AB_TG5064	AMIU00000000	Blood	Global	2007	2	2-2-2-2-2-2-2
140	AC12	ALAM00000000	Blood	Global	2011	2	2-2-2-2-2-2-2
141	AC30	ALXD00000000	Respiratory	Global	2011	2	2-2-2-2-2-2-2
142	ANC 4097	APRF00000000	Misc.	Global	2011	1	1-1-1-1-5-1-1
143	ATCC 19606 = CIP 70.34	APRG00000000	Wound	Global	before 1949	52	3-2-2-7-9-1-5
144	Ab11111	AKAQ00000000	Misc.	Global	unknown	2	2-2-2-2-2-2-2
145	Ab33333	AKAS00000000	Misc.	Global	unknown	419	3-71-2-2-5-4-14
146	Ab44444	AKAT00000000	Misc.	Global	unknown	2	2-2-2-2-2-2-2
147	BZICU-2	ALOH00000000	Respiratory	Global	2010	218	1-5-40-2-7-1-1
148	AB210	AEOX00000000	Misc.	Global	2005	2	2-2-2-2-2-2-2
149	MSP4-16	AODW00000000	Misc.	Global	2010	52	3-2-2-7-9-1-5
150	NIPH 1362	APOR00000000	Unknown	Global	2000	47	2-13-2-2-2-2-2
151	NIPH 146	APOU00000000	Unknown	Global	1993	25	3-3-2-4-7-2-4
152	NIPH 1669	APOQ00000000	Unknown	Global	1997	3	3-3-2-2-3-1-3
153	NIPH 1734	APOX00000000	Unknown	Global	2001	15	6-6-8-2-3-5-4
154	NIPH 190	APPL00000000	Unknown	Global	1993	9	3-1-5-3-6-1-3
155	NIPH 201	APQV00000000	Respiratory	Global	1992	38	3-2-15-6-6-4-5
156	NIPH 2061	APOW00000000	Unknown	Global	2003	2	2-2-2-2-2-2-2
157	NIPH 24	APOF00000000	Urinary	Global	1991	2	2-2-2-2-2-2-2
158	NIPH 290	APRD00000000	Urinary	Global	1994	1	1-1-1-1-5-1-1
159	AB900	ABXK00000000	Skin	WRAIR	2003	49	3-3-6-2-3-1-5
160	NIPH 329	APQY00000000	Respiratory	Global	1994	11	1-2-6-2-3-4-4
161	NIPH 335	APQX00000000	Respiratory	Global	1994	10	1-3-2-1-4-4-4
162	NIPH 410	ATGJ00000000	Blood	Global	1996	39	10-4-3-2-13-1-2
163	NIPH 527	APQW00000000	Unknown	Global	1984	1	1-1-1-1-5-1-1
164	NIPH 528	APRB00000000	Unknown	Global	1982	2	2-2-2-2-2-2-2
165	NIPH 60	APPM00000000	Respiratory	Global	1992	34	8-1-14-3-12-1-13
166	NIPH 601	APQZ00000000	Urinary	Global	1993	40	1-2-2-2-5-1-14
167	NIPH 615	APOV00000000	Respiratory	Global	1994	12	3-5-7-1-7-2-6
168	NIPH 67	APRA00000000	Respiratory	Global	1992	35	9-3-2-2-5-4-14
169	NIPH 70	APRC00000000	Respiratory	Global	1992	36	1-2-2-2-3-1-2
170	ABNIH1	AFSZ00000000	Unknown	Global	2007	novel	NEW-2-2-2-2-2-2
171	NIPH 80	APRE00000000	Blood	Global	1993	37	3-2-2-2-7-1-2
172	PR07	APWV00000000	Blood	Global	2012	239	1-4-2-2-7-1-4
173	TG19582	AMIV00000000	Unknown	Global	unknown	1	1-1-1-1-5-1-1
174	W6976	AIEG00000000	Misc.	Global	2009	2	2-2-2-2-2-2-2
175	W7282	AIEH00000000	Internal	Global	2009	2	2-2-2-2-2-2-2

#	Strain	Accession	Source	Category	Year	ST	MLST allelic profile (cpn60:fusA:gltA:pyrG:recA:rplB:rpoB)
176	ZWS1122	AMGR00000000	Blood	Global	2011	2	2-2-2-2-2-2-2
177	ZWS1219	AMGS00000000	Blood	Global	2011	2	2-2-2-2-2-2-2
178	ABNIH2	AFTA00000000	Unknown	Global	2007	2	2-2-2-2-2-2-2
179	ABNIH3	AFTB00000000	Unknown	Global	2007	415	2-2-2-2-68-2-2
180	ABNIH4	AFTC00000000	Unknown	Global	2007	2	2-2-2-2-2-2-2
181	UMB001	AEPK00000000	Blood	Global	2008	2	2-2-2-2-2-2-2
182	UMB002	AEPL00000000	Skin	Global	2008	16	7-7-2-2-8-4-4
183	UMB003	AEPM00000000	Wound	Global	2008	25	3-3-2-4-7-2-4
184	WM99c	AERY00000000	Misc.	Global	1999	2	2-2-2-2-2-2-2
185	48055	AOSP00000000	Respiratory	Global	2010	2	2-2-2-2-2-2-2
186	53264	ALPW00000000	Respiratory	Global	2010	2	2-2-2-2-2-2-2
187	AB-HKU3-08	ATHY00000000	Respiratory	Global	2008	215	27-2-7-2-2-1-2
188	AB-HKU3-10	ATHZ00000000	Misc.	Global	2010	215	27-2-7-2-2-1-2
189	AB1H8	ANNC00000000	Respiratory	Global	2005	2	2-2-2-2-2-2-2
190	AB405E4	ANND00000000	Blood	Global	2006	516	3-4-2-2-9-1-2
191	AB4857 (a.k.a. MRSN 939)	AHAG00000000	Internal	MRSN	2009	3	3-3-2-2-3-1-3
192	AB4A3	AOLU00000000	Wound	Global	2006	255	3-37-2-2-42-1-14
193	AB5256 (a.k.a. MRSN 961)	AHAI00000000	Blood	MRSN	2009	25	3-3-2-4-7-2-4
194	AB5711 (a.k.a. MRSN 1310)	AHAJ00000000	Blood	MRSN	2009	2	2-2-2-2-2-2-2
195	ABIsac_ColiR	CAKB00000000	Respiratory	Global	2011	novel	2-NEW-2-2-2-2-2
196	ABIsac_ColiS	CAKA00000000	Respiratory	Global	2011	2	2-2-2-2-2-2-2
197	ABNIH10	APAZ00000000	Blood	Global	2009	1	1-1-1-1-5-1-1
198	ABNIH11	APBA00000000	Respiratory	Global	2009	1	1-1-1-1-5-1-1
199	ABNIH13	APBB00000000	Respiratory	Global	2007	2	2-2-2-2-2-2-2
200	ABNIH14	APBC00000000	Respiratory	Global	2007	2	2-2-2-2-2-2-2
201	ABNIH15	APBD00000000	Respiratory	Global	2007	2	2-2-2-2-2-2-2
202	ABNIH16	APBE00000000	Respiratory	Global	2007	2	2-2-2-2-2-2-2
203	ABNIH17	APBF00000000	Respiratory	Global	2007	2	2-2-2-2-2-2-2
204	ABNIH18	APBG00000000	Respiratory	Global	2007	2	2-2-2-2-2-2-2
205	ABNIH19	APBH00000000	Respiratory	Global	2009	1	1-1-1-1-5-1-1
206	ABNIH20	APBI00000000	Respiratory	Global	2009	2	2-2-2-2-2-2-2
207	ABNIH22	APBJ00000000	Respiratory	Global	2009	2	2-2-2-2-2-2-2
208	ABNIH23	APBK00000000	Respiratory	Global	2009	2	2-2-2-2-2-2-2
209	ABNIH24	APBL00000000	Respiratory	Global	2008	2	2-2-2-2-2-2-2
210	ABNIH25	APBM00000000	Respiratory	Global	2007	2	2-2-2-2-2-2-2
211	ABNIH26	AOGD00000000	Respiratory	Global	2007	2	2-2-2-2-2-2-2
212	ABNIH5	APAW00000000	Respiratory	Global	2007	2	2-2-2-2-2-2-2
213	ABNIH6	APAX00000000	Respiratory	Global	2009	1	1-1-1-1-5-1-1
214	ABNIH7	APAY00000000	Respiratory	Global	2009	novel	1-1-1-NEW-5-1-1
215	AB_1536-8	AMHA00000000	Unknown	Global	2006	413	1-3-2-2-5-8-12
216	AB_1582-8	AMHB00000000	Respiratory	Global	2006	2	2-2-2-2-2-2-2
217	AB_1583-8	AMHC00000000	Unknown	Global	2006	422	26-72-2-2-29-4-5
218	AB_1594-8	AMHD00000000	Respiratory	Global	2006	417	1-2-2-2-11-1-5
219	AB_1595-8	AMHE00000000	Urinary	Global	2006	2	2-2-2-2-2-2-2
220	AB_1649-8	AMHF00000000	Respiratory	Global	2006	113	3-3-3-4-7-4-4
221	AB_1650-8	AMHG00000000	Internal	Global	2006	113	3-3-3-4-7-4-4
222	AB_1766_8	AMJO00000000	Blood	Global	2006	2	2-2-2-2-2-2-2
223	AB_2007-09-110-01-7	AMHH00000000	Surface	Global	2007	429	3-74-2-3-6-1-16
224	AB_2007-16-25-01-7	AMHI00000000	Surface	Global	2007	241	40-3-15-2-40-4-4
225	AB_2007-16-27-01	AMHJ00000000	Surface	Global	2007	241	40-3-15-2-40-4-4
226	AB_2008-15-34-7	AMHK00000000	Respiratory	Global	2008	2	2-2-2-2-2-2-2
227	AB_2008-15-45	AMHL00000000	Respiratory	Global	2008	415	2-2-2-2-68-2-2
228	AB_2008-15-52	AMHM00000000	Wound	Global	2008	416	1-2-2-2-4-1-4
229	AB_2008-15-69	AMHN00000000	Unknown	Global	2008	25	3-3-2-4-7-2-4
230	AB_2008-15-70	AMHO00000000	Respiratory	Global	2008	415	2-2-2-2-68-2-2
231	AB_2008-15-71	AMHP00000000	Respiratory	Global	2008	416	1-2-2-2-4-1-4
232	AB_2008-23-01-01-7	AMHQ00000000	Surface	Global	2008	417	1-2-2-2-11-1-5
233	AB_2008-23-07-01-7	AMHR00000000	Surface	Global	2008	2	2-2-2-2-2-2-2
234	AB_2009-04-01-7	AMHS00000000	Wound	Global	2009	417	1-2-2-2-11-1-5
235	AB_2009-04-02-7	AMHT00000000	Respiratory	Global	2009	2	2-2-2-2-2-2-2
236	AB_515-8	AMHU00000000	Respiratory	Global	unknown	2	2-2-2-2-2-2-2
237	AB_908-12	AMHV00000000	Surface	Global	2007	2	2-2-2-2-2-2-2
238	AB_908-13	AMHW00000000	Urinary	Global	2007	1	1-1-1-1-5-1-1
239	AB_908-14-7	AMHX00000000	Respiratory	Global	2007	2	2-2-2-2-2-2-2
240	AB_909-01-7	AMHY00000000	Blood	Global	2007	417	1-2-2-2-11-1-5
241	AB_909-02-7	AMHZ00000000	Respiratory	Global	2007	1	1-1-1-1-5-1-1
242	AB_909-05	AMIA00000000	Respiratory	Global	2007	2	2-2-2-2-2-2-2
243	AB_909-14-7	AMIB00000000	Wound	Global	2007	2	2-2-2-2-2-2-2
244	AB_TG19617	AMID00000000	Unknown	Global	unknown	438	3-2-2-7-9-4-5
245	AB_TG2018	AMIE00000000	Respiratory	Global	2006	417	1-2-2-2-11-1-5
246	AB_TG2022	AMIF00000000	Blood	Global	2006	2	2-2-2-2-2-2-2
247	AB_TG2023	AMIG00000000	Blood	Global	2006	2	2-2-2-2-2-2-2
248	AB_TG2026	AMIH00000000	Wound	Global	2006	2	2-2-2-2-2-2-2
249	AB_TG2028	AMII00000000	Respiratory	Global	2001	406	1-1-1-2-65-1-5
