# Supplementary material for: A novel method of consensus pan-chromosome assembly and large-scale comparative analysis reveal the highly flexible pan-genome of Acinetobacter baumannii
Source: Genome Biol. 2015 Jul 21;16(1):143. doi: 10.1186/s13059-015-0701-6 (PMC4507327; doi:10.1186/s13059-015-0701-6)
Supplement: Additional file 10: Table S4. — Chromosomally encoded antibiotic resistance genes found within fGIs and fGRs. [file 13059_2015_701_MOESM10_ESM.pdf]

Table S4. Chromosomally-encoded Antibiotic Resistance Genes Found Within fGIs and fGRs

cluster_id	Assembly_fGIs	fGR_id	Paralog	DR Class	Description
4425	58	CL_INS_20	4425	lin/str/phe/lin/mac	aminoglycoside/hydroxyurea antibiotic resistance kinase
4426	58	CL_INS_20	4426	lin/str/phe/lin/mac	streptomycin 3''-kinase
4421	58	CL_INS_20	4330	Antibiotic Efflux - Tetracycline	transporter, major facilitator family protein
4409	44	CL_INS_20	237	beta-lactam	resolvase, N-terminal domain protein
234	44	CL_INS_20	234	Sulfonamide	dihydropteroate synthase
4410	44	CL_INS_20	---	beta-lactam	beta-lactamase
4420	44	CL_INS_20	116	aminoglycoside	streptomycin 3''-adenylyltransferase
5954	65	CL_INS_20	5954	Antibiotic Efflux	ABC transporter, ATP-binding protein
5955	65	CL_INS_20	116	lin/str/phe/lin/mac	phosphotransferase enzyme family protein
5151	65	CL_INS_20	234	Sulfonamide	dihydropteroate synthase
4417	65	CL_INS_20	4417	chloramphenicol	chloramphenicol O-acetyltransferase
6232	65	CL_INS_20	116	aminoglycoside	streptomycin 3''-adenylyltransferase
5951	65	CL_INS_20	---	aminoglycoside	aminoglycoside resistance ribosomal RNA methyltransferase, FmrO/ArmA family
5949	65	CL_INS_20	231	aminoglycoside	
4418	44	CL_INS_20	116	aminoglycoside	FR47-like protein
8930	3085	CL_INS_20	116	aminoglycoside	gentamicin 2''-nucleotidyltransferase
4946	569	CL_INS_20	---	aminoglycoside	acetyltransferase (GNAT) domain protein
7967	569	CL_INS_20	116	aminoglycoside	streptomycin 3''-adenylyltransferase domain protein
5713	212	CL_INS_20	5713	Antibiotic Efflux	drug resistance transporter, Bcr/CflA family
5160	44	CL_INS_20	---	chloramphenicol	chloramphenicol O-acetyltransferase
5731	439	CL_INS_20	---	Antibiotic Efflux	drug resistance transporter, Bcr/CflA subfamily
9666	46	CL_INS_20	294	Antibiotic Efflux	H-NS histone family protein
5729	439	CL_INS_20	4330	Antibiotic Efflux - Tetracycline	transporter, major facilitator family protein
15537	459	CL_INS_20	---	beta-lactam	metallo-beta-lactamase domain protein
12525	112	CL_INS_20	---	Antibiotic Efflux	putative multidrug efflux pump BpeE
12526	112	CL_INS_20	2490	Antibiotic Efflux	multidrug efflux pump BpeF
12527	112	CL_INS_20	---	Antibiotic Efflux	outer membrane efflux protein OprM
4298	16	CL_INS_49	4298	Antibiotic Efflux	cation efflux system protein CusA
1903	540	CL_INS_81	1903	beta-lactam	putative hemagglutinin
5926	223	CL_INS_93	294	Antibiotic Efflux	H-NS histone family protein
4772	401	CL_INS_100	1556	beta-lactam	penicillin-binding protein, transpeptidase domain protein
17354	903	CL_INS_214	5713	Antibiotic Efflux	drug resistance transporter, Bcr/CflA family
7769	422	CL_INS_243	1556	beta-lactam	penicillin-binding protein, transpeptidase domain protein
6736	166	CL_INS_245	6736	beta-lactam	filamentous hemagglutinin family N-terminal domain protein
4330	2982	CL_INS_311	4330	Antibiotic Efflux - Tetracycline	transporter, major facilitator family protein
3908	775	CL_INS_20, CL_INS_100	234	Sulfonamide	dihydropteroate synthase
7766	426	CL_INS_20, CL_INS_157	---	trimethoprim	dihydrofolate reductase
7400	816	CL_INS_135, CL_INS_136	1903	beta-lactam	putative hemagglutinin
9951	684	CL_INS_283, CL_INS_284	1556	beta-lactam	penicillin-binding protein, transpeptidase domain protein
7765	426	CL_INS_20, CL_INS_157	---	aminoglycoside	acetyltransferase, GNAT family
4244	41	CL_INS_20, CL_INS_74	---	Antibiotic Efflux	MATE efflux family protein
4925	606	CL_INS_50, CL_INS_51, CL_INS_233	---	beta-lactam	beta-lactamase
11977	355	CL_INS_74, CL_INS_257	---	Antibiotic Efflux	efflux transporter, RND family, MFP subunit
11978	355	CL_INS_74, CL_INS_257	---	Antibiotic Efflux	multidrug efflux protein
15516	2301	CL_INS_78, CL_INS_79	6182	aminoglycoside	choline/ethanolamine kinase
