# Supplementary material for: A novel method of consensus pan-chromosome assembly and large-scale comparative analysis reveal the highly flexible pan-genome of Acinetobacter baumannii
Source: Genome Biol. 2015 Jul 21;16(1):143. doi: 10.1186/s13059-015-0701-6 (PMC4507327; doi:10.1186/s13059-015-0701-6)
Supplement: Additional file 11: Table S5. — Resistance island target sites. [file 13059_2015_701_MOESM11_ESM.pdf]

Table S5. Resistance Island Target Sites

RI insertion locus	ACICU gene ID	Reference RI	Reference isolate	Antibiotic resistance gene cassette	References*
**(a) RI insertion hot spots and reference RIs used for the identification of RI signatures**					
**comM**	ACICU_00241	AbaR3	AB0057	aacC1-orfP-orfQ-aadA1	Krizova et al. 2011
		AbGRI1	MDR-TJ	sul2, tetA-tetR, strB-strA, bla_{OXA}-23	Nigro et al. 2013
		AbaR4	D36	bla_{OXA}-23	Hamidian and Hall, 2011
**pho**	ACICU_00473	AbaR4	AB0057	bla_{OXA}-23	Adams et al. 2008, Seputiene et al. 2012
**astA**	ACICU_01137 to ACICU_01145	AbGRI2-2	MDR-TJ	aacC1-$(orfP)_3$-orfQ-aadA1	Nigro et al. 2013
**Acetyl-transferase**	ACICU_02399	Tn1548	TYTH-1	sul1-aadA-cat-aac6, armA	Galimand et al. 2005, Wright et al. 2014
**Hypothetical protein**	ACICU_02698	AbaR27	BJAB0715	tetA, strA-strB, sul2	Zhu et al. 2013
**(b) Novel RI-related genomic features identified in *A. baumannii* isolates**					
**Acetyl-transferase**	ACICU_02399	Non-RI genomic fragment	n.a.	A 7.8 kb genomic fragment with >90% identity to *A. calcoaceticus*	Di Nocera et al. 2011, Sahl et al. 2011, this study
**Acyl-CoA synthetase**	ACICU_00139 to ACICU_00143	Composite IS26 RI	n.a.	sulI-qacEdelta1-aadB-intI1 , tetR-tetA	Domingues et al. 2012, this study

*References cited in this table:

Adams MD, Goglin K, Molyneaux N, Hujer KM, Lavender H, Jamison JJ, MacDonald IJ, Martin KM, Russo T, Campagnari AA et al. 2008. Comparative genome sequence analysis of multidrug-resistant *Acinetobacter baumannii*. J Bacteriol 190(24): 8053-8064.

Di Nocera PP, Rocco F, Giannouli M, Triassi M, Zarrilli R. 2011. Genome organization of epidemic *Acinetobacter baumannii* strains. BMC Microbiol 11: 224.

Domingues S, Harms K, Fricke WF, Johnsen PJ, da Silva GJ, Nielsen KM. 2012. Natural transformation facilitates transfer of transposons, integrons and gene cassettes between bacterial species. PLoS pathogens 8(8): e1002837.

Galimand M, Sabtcheva S, Courvalin P, Lambert T. 2005. Worldwide disseminated *armA* aminoglycoside resistance methylase gene is borne by composite transposon Tn*1548*. J Antimicrob Chemother 49(7): 2949-2953.

Hamidian M, Hall RM. 2011. AbaR4 replaces AbaR3 in a carbapenem-resistant *Acinetobacter baumannii* isolate belonging to global clone 1 from an Australian hospital. J Antimicrob Chemother 66(11): 2484-2491.

Krizova L, Dijkshoorn L, Nemec A. 2011. Diversity and evolution of AbaR genomic resistance islands in *Acinetobacter baumannii* strains of European clone I. Antimicrob Agents Chemother 55(7): 3201-3206.

Nigro SJ, Farrugia DN, Paulsen IT, Hall RM. 2013. A novel family of genomic resistance islands, AbGRI2, contributing to aminoglycoside resistance in *Acinetobacter baumannii* isolates belonging to global clone 2. J Antimicrob Chemother 68(3): 554-557.

Sahl JW, Johnson JK, Harris AD, Phillippy AM, Hsiao WW, Thom KA, Rasko DA. 2011. Genomic comparison of multi-drug resistant invasive and colonizing *Acinetobacter baumannii* isolated from diverse human body sites reveals genomic plasticity. BMC Genomics 12: 291.

Seputiene V, Povilonis J, Suziedeliene E. 2012. Novel variants of AbaR resistance islands with a common backbone in *Acinetobacter baumannii* isolates of European clone II. Antimicrob Agents Chemother 56(4): 1969-1973.

Wright MS, Haft DH, Harkins DM, Perez F, Hujer KM, Bajaksouzian S, Benard MF, Jacobs MR, Bonomo RA, Adams MD. 2014. New insights into dissemination and variation of the health care-associated pathogen *Acinetobacter baumannii* from genomic analysis. MBio 5(1): e00963-00913.

Zhu L, Yan Z, Zhang Z, Zhou Q, Zhou J, Wakeland EK, Fang X, Xuan Z, Shen D, Li QZ. 2013. Complete genome analysis of three *Acinetobacter baumannii* clinical isolates in China for insight into the diversification of drug resistance elements. PLoS One 8(6): e66584.
