# Supplementary material for: A novel method of consensus pan-chromosome assembly and large-scale comparative analysis reveal the highly flexible pan-genome of Acinetobacter baumannii
Source: Genome Biol. 2015 Jul 21;16(1):143. doi: 10.1186/s13059-015-0701-6 (PMC4507327; doi:10.1186/s13059-015-0701-6)
Supplement: Additional file 12: Table S6. — a Distribution of RI-positive isolates among A. baumannii genomes analyzed. b Summary of major RI signatures identified among CC1, CC2, CC3, and ST 25 isolates. All RI signatures identified in (c) CC1, (d) CC2, (e) CC3, (f) ST25, and (g) other isolates. h A list of RI target genes showing total gene length detected when intact and having no RI insertion, or carrying a RI insertion with junction fragments. i Total gene length of the comM target gene detected in a collection of finished A. baumannii genomes. [file 13059_2015_701_MOESM12_ESM.pdf]

Table S6. (A) Distribution of RI-positive isolates among *A. baumannii* genomes analyzed

Clone type	WRAIR			MRSN			US hospital			Global			Total		
	Isolate analyzed	RI positive	% positive	Isolate analyzed	RI positive	% positive	Isolate analyzed	RI positive	% positive	Isolate analyzed	RI positive	% positive	Isolate analyzed	RI positive	% positive
**CC1**	12	10	83.3	4	4	100.0	0	0	0.0	16	12	75.0	32	26	81.3
**CC2**	8	7	87.5	2	2	100.0	39	37	94.9	69	59	85.5	118	105	89.0
**CC3**	6	6	100.0	1	1	100.0	0	0	0.0	1	1	100.0	8	8	100.0
**ST25**	2	2	100.0	1	1	100.0	0	0	0.0	4	4	100.0	7	7	100.0
**Other**	20	8	40.0	3	3	100.0	10	1	10.0	49	15	30.6	82	27	32.9
**Total**	48	33	68.8	11	11	100.0	49	38	77.6	139	91	65.5	**247**	**173**	70.0

Table S6. (B) Summary of major RI signatures identified among CC1, CC2, CC3 and ST 25 isolates

Clone type	RI insertion locus							Number of isolates positive for an RI signature[1]	Examples of isolates[2]
	comM		*pho*	*astA*	AcetylT		AcylCS		
	AbaR3/ AbaR4	AbGRI1	AbaR4	AbGRI2	Tn1548	7.8 kb non-RI	Composite IS26		
CC1	■							15	NIPH 527, NIPH 290, AYE, *Naval-83, IS-235, IS-251, IS-58, MRSN 58*
	■		■					7	AB0057, *MRSN 7339, Canada BC-5, Canada BC1*
CC2	■							2	ABIsac_ColiS, ABIsac_ColiR
		■						39	NIPH 528, 1656-2, *NIPH 24, OIFC338*
		■			■			46	AB210, MDR-ZJ06, TYTH-1, BJAB07104, BJAB0868
		■		■				13	WM99c, TCDC-AB0715, *OIFC189, Naval-113, OIFC180, Naval-17*
		■		■	■			1	MDR-TJ
CC3							■	8	NIPH 1669, *OIFC137, OIFC109, Naval-81, Naval-13, WC-A-694, IS-123*, AB4857
ST25[3]						■		7	NIPH 146, *OIFC143, Naval-18*, UMB003, AB_2008-15-69, 4190, AB5256
Total								138	

Key:

(1) A total 35 isolates belonging to non-major clone types or non-major RI signatures not shown. RI classification for all RI-positive isolates can be found in Table S5C-G.

(2) Isolate name: finished genomes (blue), pre-2000 isolates (red), sequenced in this study (italics)

(3) A 7.8 kb non-RI type genomic insertion with 99% identity to *A. calcoaceticus* was identified at the acetylT gene locus in ST25 isolates

Table S6. (C) RI signatures identified in CC1 isolates

RI signature	Isolate	ST type	Allele summary
comM/AbaR3	NIPH 527	1	1-1-1-1-5-1-1
comM/AbaR3	NIPH 290	1	1-1-1-1-5-1-1
comM/AbaR3	AYE	1	1-1-1-1-5-1-1
comM/AbaR3	IS-235	1	1-1-1-1-5-1-1
comM/AbaR3	IS-251	1	1-1-1-1-5-1-1
comM/AbaR3	IS-58	1	1-1-1-1-5-1-1
comM/AbaR3	MRSN 58	1	1-1-1-1-5-1-1
comM/AbaR3	ANC 4097	1	1-1-1-1-5-1-1
comM/AbaR3	AB5075	1	1-1-1-1-5-1-1
comM/AbaR4	AB056	1	1-1-1-1-5-1-1
comM/AbaR4	ABNIH10	1	1-1-1-1-5-1-1
comM/AbaR4	ABNIH11	1	1-1-1-1-5-1-1
comM/AbaR4	ABNIH6	1	1-1-1-1-5-1-1
comM/AbaR3 + pho	MRSN 7339	1	1-1-1-1-5-1-1
comM/AbaR3 + pho	AB0057	1	1-1-1-1-5-1-1
comM/AbaR3 + pho	1605	1	1-1-1-1-5-1-1
comM/AbaR3 + pho	Canada BC-5	1	1-1-1-1-5-1-1
comM/AbaR3 + pho	Canada BC1	1	1-1-1-1-5-1-1
comM/AbaR4 + pho	AB_908-13	1	1-1-1-1-5-1-1
comM/AbaR4 + pho	AB_909-02-7	1	1-1-1-1-5-1-1
comM/other	ABNIH19	1	1-1-1-1-5-1-1
comM/other	OIFC074	19	1-2-1-1-5-1-1
comM/other	Naval-21	19	1-2-1-1-5-1-1
comM/AbaR3	Naval-83	20	3-1-1-1-5-1-1
comM/AbaR4	MRSN 3527	81	1-1-1-1-5-1-2
comM/AbaR4	ABNIH7	novel	1-1-1-NEW-5-1-1

Key:
AbaR3 = Aba3-like
AbaR4 = Aba4-like
AbGRI1 = AbGRI1-like
acetylT = acetyltransferase
acylCS = acyl-CoA-synthetase

Table S6. (D) RI signatures identified in CC2 isolates

RI signature	Isolate	ST type	Allele summary	Comments
comM/AbGRI1	NIPH 528	2	2-2-2-2-2-2-2	
comM/AbGRI1	NIPH 24	2	2-2-2-2-2-2-2	
comM/AbGRI1	OIFC338	2	2-2-2-2-2-2-2	
comM/AbGRI1	NIPH 2061	2	2-2-2-2-2-2-2	
comM/AbGRI1	AB_1582-8	2	2-2-2-2-2-2-2	
comM/AbGRI1	AB_TG2022	2	2-2-2-2-2-2-2	
comM/AbGRI1	AB_TG2023	2	2-2-2-2-2-2-2	
comM/AbGRI1	UH0707	2	2-2-2-2-2-2-2	
comM/AbGRI1	UH0807	2	2-2-2-2-2-2-2	
comM/AbGRI1	UH10707	2	2-2-2-2-2-2-2	
comM/AbGRI1	UH2707	2	2-2-2-2-2-2-2	
comM/AbGRI1	UH2907	2	2-2-2-2-2-2-2	
comM/AbGRI1	UH7707	2	2-2-2-2-2-2-2	
comM/AbGRI1	AB_TG5064	2	2-2-2-2-2-2-2	
comM/AbGRI1	ABNIH2	2	2-2-2-2-2-2-2	
comM/AbGRI1	ABNIH13	2	2-2-2-2-2-2-2	
comM/AbGRI1	ABNIH14	2	2-2-2-2-2-2-2	
comM/AbGRI1	ABNIH15	2	2-2-2-2-2-2-2	
comM/AbGRI1	ABNIH16	2	2-2-2-2-2-2-2	
comM/AbGRI1	ABNIH17	2	2-2-2-2-2-2-2	
comM/AbGRI1	ABNIH26	2	2-2-2-2-2-2-2	
comM/AbGRI1	AB_908-12	2	2-2-2-2-2-2-2	
comM/AbGRI1	AB_908-14-7	2	2-2-2-2-2-2-2	
comM/AbGRI1	AB_909-05	2	2-2-2-2-2-2-2	
comM/AbGRI1	UH12308	2	2-2-2-2-2-2-2	
comM/AbGRI1	UH12408	2	2-2-2-2-2-2-2	
comM/AbGRI1	UH12808	2	2-2-2-2-2-2-2	
comM/AbGRI1	UH16108	2	2-2-2-2-2-2-2	
comM/AbGRI1	ABNIH24	2	2-2-2-2-2-2-2	
comM/AbGRI1	AB5711	2	2-2-2-2-2-2-2	
comM/AbGRI1	ABNIH20	2	2-2-2-2-2-2-2	
comM/AbGRI1	ABNIH22	2	2-2-2-2-2-2-2	
comM/AbGRI1	ABNIH23	2	2-2-2-2-2-2-2	
comM/AbGRI1	AB_2009-04-02-7	2	2-2-2-2-2-2-2	
comM/AbGRI1	1656-2	2	2-2-2-2-2-2-2	
comM/AbGRI1	Ab11111	2	2-2-2-2-2-2-2	
comM/AbGRI1 + acetylT	AB_TG27323	2	2-2-2-2-2-2-2	
comM/AbGRI1 + acetylT	AB_TG27327	2	2-2-2-2-2-2-2	
comM/AbGRI1 + acetylT	AB_TG27331	2	2-2-2-2-2-2-2	
comM/AbGRI1 + acetylT	AB_TG27335	2	2-2-2-2-2-2-2	
comM/AbGRI1 + acetylT	AB210	2	2-2-2-2-2-2-2	
comM/AbGRI1 + acetylT	MDR-ZJ06	2	2-2-2-2-2-2-2	
comM/AbGRI1 + acetylT	AB_1766_8	2	2-2-2-2-2-2-2	
comM/AbGRI1 + acetylT	AB_TG2026	2	2-2-2-2-2-2-2	
comM/AbGRI1 + acetylT	UH0207	2	2-2-2-2-2-2-2	
comM/AbGRI1 + acetylT	UH10107	2	2-2-2-2-2-2-2	
comM/AbGRI1 + acetylT	UH3807	2	2-2-2-2-2-2-2	
comM/AbGRI1 + acetylT	UH5307	2	2-2-2-2-2-2-2	

RI signature	Isolate	ST type	Allele summary	Comments
comM/AbGRI1 + acetylT	UH5707	2	2-2-2-2-2-2-2	
comM/AbGRI1 + acetylT	UH6107	2	2-2-2-2-2-2-2	
comM/AbGRI1 + acetylT	UH6207	2	2-2-2-2-2-2-2	
comM/AbGRI1 + acetylT	UH7807	2	2-2-2-2-2-2-2	
comM/AbGRI1 + acetylT	UH8107	2	2-2-2-2-2-2-2	
comM/AbGRI1 + acetylT	UH8807	2	2-2-2-2-2-2-2	
comM/AbGRI1 + acetylT	UH9007	2	2-2-2-2-2-2-2	
comM/AbGRI1 + acetylT	UH9707	2	2-2-2-2-2-2-2	
comM/AbGRI1 + acetylT	UH9907	2	2-2-2-2-2-2-2	
comM/AbGRI1 + acetylT	AB_TG2631	2	2-2-2-2-2-2-2	
comM/AbGRI1 + acetylT	AB_909-14-7	2	2-2-2-2-2-2-2	
comM/AbGRI1 + acetylT	UH11608	2	2-2-2-2-2-2-2	
comM/AbGRI1 + acetylT	UH13908	2	2-2-2-2-2-2-2	
comM/AbGRI1 + acetylT	UH14508	2	2-2-2-2-2-2-2	
comM/AbGRI1 + acetylT	UH15208	2	2-2-2-2-2-2-2	
comM/AbGRI1 + acetylT	UH16008	2	2-2-2-2-2-2-2	
comM/AbGRI1 + acetylT	UH19908	2	2-2-2-2-2-2-2	
comM/AbGRI1 + acetylT	TYTH-1	2	2-2-2-2-2-2-2	
comM/AbGRI1 + acetylT	AB_2008-23-07-01-7	2	2-2-2-2-2-2-2	
comM/AbGRI1 + acetylT	W6976	2	2-2-2-2-2-2-2	
comM/AbGRI1 + acetylT	W7282	2	2-2-2-2-2-2-2	
comM/AbGRI1 + acetylT	AC12	2	2-2-2-2-2-2-2	
comM/AbGRI1 + acetylT	AC30	2	2-2-2-2-2-2-2	
comM/AbGRI1 + acetylT	ZWS1122	2	2-2-2-2-2-2-2	
comM/AbGRI1 + acetylT	ZWS1219	2	2-2-2-2-2-2-2	
comM/AbGRI1 + acetylT	BJAB07104	2	2-2-2-2-2-2-2	plasmid
comM/AbGRI1 + acetylT	BJAB0868	2	2-2-2-2-2-2-2	plasmid
comM/AbGRI1 + acetylT	AB_515-8	2	2-2-2-2-2-2-2	only 5' gene fragment found
comM/AbGRI1 + acetylT	UH10007	2	2-2-2-2-2-2-2	only 3' gene fragment found
comM/AbGRI1 + acetylT	UH8407	2	2-2-2-2-2-2-2	partial, but have resistance genes
comM/AbGRI1 + acetylT	UH8707	2	2-2-2-2-2-2-2	partial, but have resistance genes
comM/AbGRI1 + acetylT	AB_1595-8	2	2-2-2-2-2-2-2	only 5' gene fragment found
comM/AbGRI1 + acetylT	UH20108	2	2-2-2-2-2-2-2	partial, no resistance genes
comM/AbGRI1 + acetylT	UH18608	2	2-2-2-2-2-2-2	partial, no resistance genes
comM/AbGRI1 + astA	WM99c	2	2-2-2-2-2-2-2	
comM/AbGRI1 + astA	OIFC189	2	2-2-2-2-2-2-2	
comM/AbGRI1 + astA	AB1H8	2	2-2-2-2-2-2-2	
comM/AbGRI1 + astA	Naval-113	2	2-2-2-2-2-2-2	
comM/AbGRI1 + astA	UH1007	2	2-2-2-2-2-2-2	
comM/AbGRI1 + astA	UH2107	2	2-2-2-2-2-2-2	
comM/AbGRI1 + astA	UH2307	2	2-2-2-2-2-2-2	
comM/AbGRI1 + astA	48055	2	2-2-2-2-2-2-2	
comM/AbGRI1 + astA	53264	2	2-2-2-2-2-2-2	
comM/AbGRI1 + astA	TCDC-AB0715	2	2-2-2-2-2-2-2	
comM/AbGRI1 + astA	OIFC180	2	2-2-2-2-2-2-2	
comM/AbGRI1 + astA	Naval-17	2	2-2-2-2-2-2-2	
comM/AbGRI1 + astA	6014059	2	2-2-2-2-2-2-2	
astA	Naval-78	2	2-2-2-2-2-2-2	
comM/AbGRI1 + astA + acetylT	MDR-TJ	2	2-2-2-2-2-2-2	
comM/other	MRSN 7341	2	2-2-2-2-2-2-2	

RI signature	Isolate	ST type	Allele summary	Comments
comM/other	ACICU	2	2-2-2-2-2-2-2	
comM/AbaR4	ABIsac_ColiS	2	2-2-2-2-2-2-2	
comM/AbaR4	ABIsac_ColiR	novel	2-NEW-2-2-2-2-2	
comM/AbGRI1	ABNIH3	415	2-2-2-2-68-2-2	
comM/AbGRI1	AB_2008-15-45	415	2-2-2-2-68-2-2	
comM/AbGRI1	AB_2008-15-70	415	2-2-2-2-68-2-2	
astA	IS-143	414	2-2-2-2-2-37-2	

Key:

AbaR3 = Aba3-like
AbaR4 = Aba4-like
AbGRI1 = AbGRI1-like
acetylT = acetyltransferase
acylCS = acyl-CoA-synthetase

Table S6. (E) RI signatures identified in CC3 isolates

RI signature	Isolate	ST type	Allele summary
acylCS/composite IS26	NIPH 1669	3	3-3-2-2-3-1-3
acylCS/composite IS26	OIFC137	3	3-3-2-2-3-1-3
acylCS/composite IS26	OIFC109	3	3-3-2-2-3-1-3
acylCS/composite IS26	Naval-81	3	3-3-2-2-3-1-3
acylCS/composite IS26	Naval-13	3	3-3-2-2-3-1-3
acylCS/composite IS26	WC-A-694	3	3-3-2-2-3-1-3
acylCS/composite IS26	IS-123	3	3-3-2-2-3-1-3
acylCS/composite IS26	AB4857	3	3-3-2-2-3-1-3

Key:
AbaR3 = Aba3-like
AbaR4 = Aba4-like
AbGRI1 = AbGRI1-like
acetylT = acetyltransferase
acylCS = acyl-CoA-synthetase

Table S6. (F) RI signatures identified in ST25 isolates

RI signature	Isolate	ST type	Allele summary
acetylT/7.8kb non-RI	NIPH 146	25	3-3-2-4-7-2-4
acetylT/7.8kb non-RI	UMB003	25	3-3-2-4-7-2-4
acetylT/7.8kb non-RI	AB_2008-15-69	25	3-3-2-4-7-2-4
acetylT/7.8kb non-RI	4190	25	3-3-2-4-7-2-4
acetylT/7.8kb non-RI	OIFC143	25	3-3-2-4-7-2-4
acetylT/7.8kb non-RI	Naval-18	25	3-3-2-4-7-2-4
acetylT/7.8kb non-RI	AB5256	25	3-3-2-4-7-2-4

Key:
AbaR3 = Aba3-like
AbaR4 = Aba4-like
AbGRI1 = AbGRI1-like
acetylT = acetyltransferase
acylCS = acyl-CoA-synthetase

Table S6. (G) RI signatures identified in other isolates

RI signature	Isolate	ST type	Allele summary
comM/other	NIPH 1734	15	6-6-8-2-3-5-4
comM/heavy metal resistance	BJAB0715	23	1-3-10-1-4-4-4
comM/other	OIFC032	32	1-1-2-2-3-4-4
comM/other	OIFC087	32	1-1-2-2-3-4-4
comM/other	OIFC099	32	1-1-2-2-3-4-4
comM/other	NIPH 410	39	10-4-3-2-13-1-2
comM/heavy metal resistance	OIFC111	49	3-3-6-2-3-1-5
comM/heavy metal resistance	AB900	49	3-3-6-2-3-1-5
comM/heavy metal resistance	UH5207	78	25-3-6-2-28-1-29
comM/AbaR4	MRSN 3405	94	1-2-2-1-5-1-1
comM/AbaR4	MRSN 3942	94	1-2-2-1-5-1-1
comM/AbaR4	MRSN 4106	94	1-2-2-1-5-1-1
comM/heavy metal resistance	IS-116	136	3-2-19-25-5-2-5
comM/AbGRI1 + acetylT	AB-HKU3-08	215	27-2-7-2-2-1-2
comM/AbGRI1 + acetylT	AB-HKU3-10	215	27-2-7-2-2-1-2
comM/AbaR3	AB4A3	255	3-37-2-2-42-1-14
comM/heavy metal resistance	AB_TG2018	417	1-2-2-2-11-1-5
comM/heavy metal resistance	AB_909-01-7	417	1-2-2-2-11-1-5
comM/heavy metal resistance	AB_2008-23-01-01-7	417	1-2-2-2-11-1-5
comM/heavy metal resistance	AB_2009-04-01-7	417	1-2-2-2-11-1-5
comM/heavy metal resistance	AB_TG19617	438	3-2-2-7-9-4-5
comM/heavy metal resistance	AB_2007-09-110-01-7	429	3-74-2-3-6-1-16
comM/heavy metal resistance	WC-348	412	1-52-2-2-67-4-5
comM/other	ATCC 17978	437	3-2-2-2-30-4-28
comM/other	AB_TG27343	422	26-72-2-2-29-4-5
comM/other	AB_1583-8	422	26-72-2-2-29-4-5
comM/other	WC-A-92	431	1-4-2-1-70-1-2

Table S6. (H) A list of RI target genes showing total gene length detected when (i) intact with no RI insertion, and (ii) carrying RI insertion with junction fragments.

Host gene	Gene ID	GenBank accession	Gene hit lengths when intact (bp)	Junction fragments when RI inserted into host gene (bp)		Gene hit lengths when carrying RI (bp)
comM	ACINNAV82_0188	gb|AMSW01000123.1|:6206-7693	1,488	1-841 (841)	836-1488 (653)	1,494
pho	ABAYE3309	gi|169147133:3355198-3357390	2,193	1-100 (100)	89-2193 (2105)	2,205
astA	AB57_1175	gb|CP001182.1|:1258256-1259662	1,407	1-101 (101)		847
	AB57_1209	gb|CP001182.1|:c1300006-1298483	1,524	1-746 (746)		
acetylT	ACICU_02398	gb|CP000863.1|:2534525-2535643	1,119			
	ACICU_02399	gb|CP000863.1|:c2536153-2535701	453	1-412 (412)	404-453 (50)	1,581
EJP43116	ACIN5032_2618	gb|AFCZ02000002.1|:2672143-2673987	1,845	1-1319 (1319)	1315-1845 (531)	1,850

Table S6. (I) Total gene length of the *comM* target gene detected in a collection of finished *A. baumannii* genomes.

Finished genomes	GenBank accession	Host gene	Host gene start	Host gene end	Insertion detected in host gene (bp)	Total host gene hit length (bp)	RI insertion (this study)	RI signature (this study)	References related to RI	
AB0057	CP001182.2	comM	262,919	327,400	64,482	1,494	yes	comM/AbaR3	Adams et al, 2008	AbaR3
AYE	CU459141.1	comM	3,609,192	3,696,928	87,737	1,494	yes	comM/AbaR3	Fournier et al, 2006	AbaR1
1656-2	CP001921.1	comM	257,120	292,600	35,481	1,494	yes	comM/AbaRI1	Seputiene et al, 2012	AbaR4d
TCDC-AB0715	CP002522.1	comM	245,943	291,133	45,191	1,494	yes	comM/AbaRI1	Seputiene et al, 2013	AbaR4e
MDR-ZJ06	CP001937.1	comM	254,818	294,993	40,176	1,494	yes	comM/AbaRI1	Zhou et al, 2011	AbaR22, AbGRI2-3
TYTH-1	CP003856.1	comM	474,895	518,045	43,151	1,494	yes	comM/AbaRI1	Liu et al, 2013	RI_{TYTH-1}
MDR-TJ	CP003500.1	comM	3,671,921	3,715,056	43,136	1,494	yes	comM/AbaRI1	Huang et al, 2012	RI_{MDR-TJ}, AbGRI2-2
BJAB07104	CP003846.1	comM	263,697	308,036	44,340	1,494	yes	comM/AbaRI1	Zhu et al, 2013	AbaR25
BJAB0868	CP003849.1	comM	260,642	303,790	43,149	1,494	yes	comM/AbaRI1	Zhu et al, 2013	AbaR26
ACICU	CP000863.1	comM	264,298	265,138	841	841	yes*	comM/other	Iacono et al, 2008	AbaR2 (truncated)
ATCC 17978	CP000521.1	comM	229,708	244,303	14,596	1,494	yes	comM/other	Adams et al, 2008	no resistance determinants
AB307-0294	CP001172.1	comM	3,525,766	3,527,253	1,488	1,488	no	not assigned	Not available	
D1279779	CP003967.2	comM	236,082	237,569	1,488	1,488	no	not assigned	Not available	

*Only one junction fragment was detected by this method. ACICU carries a much shorter and truncated version of RI with only 8.9 kb (Iacono et al, 2008).
