# Supplementary material for: A novel method of consensus pan-chromosome assembly and large-scale comparative analysis reveal the highly flexible pan-genome of Acinetobacter baumannii
Source: Genome Biol. 2015 Jul 21;16(1):143. doi: 10.1186/s13059-015-0701-6 (PMC4507327; doi:10.1186/s13059-015-0701-6)
Supplement: Additional file 17: Table S7. — Virulence and fitness factors used in this study. [file 13059_2015_701_MOESM17_ESM.pdf]

Table S7. Virulence and Fitness Factors used in this Study

Row	Locus Tag	ACICU gene ID	AYE gene ID	ATCC gene ID	Annotation	References*	Gene Type
1	AB57_2080	ACICU_01823	ABAYE1823		AdeC - efflux cluster 1	Sahl et al. 2013	Efflux
2	AB57_2081	ACICU_01824	ABAYE1822	A1S_1750	AdeB - efflux cluster 1	Sahl et al. 2013	Efflux
3	AB57_2082	ACICU_01825	ABAYE1821	A1S_1751	AdeA - efflux cluster 1	Sahl et al. 2013	Efflux
4	AB57_2083	ACICU_01826	ABAYE1820	A1S_1753	AdeR (regulator) - efflux cluster 1	Sahl et al. 2013	Efflux
5	AB57_2084	ACICU_01827	ABAYE1819	A1S_1754	AdeS (regulator) - efflux cluster 1	Sahl et al. 2013	Efflux
6	A1S_2303	ACICU_02502	ABAYE1176	A1S_2303	AdeL (regulator) - efflux cluster 2	Coyne et al. 2010	Efflux
7	A1S_2304	ACICU_02504	ABAYE1174	A1S_2304	AdeF - efflux cluster 2	Coyne et al. 2010	Efflux
8	A1S_2305	ACICU_02505	ABAYE1173	A1S_2305	AdeG - efflux cluster 2	Coyne et al. 2010	Efflux
9	A1S_2306	ACICU_02506	ABAYE1172	A1S_2306	AdeH - efflux cluster 2	Coyne et al. 2010	Efflux
10	AB57_3156	ACICU_02987	ABAYE0748	A1S_2735	AdeI - efflux cluster 3	Sahl et al. 2013	Efflux
11	AB57_3158	ACICU_02989	ABAYE0746	A1S_2737	AdeK - efflux cluster 3	Sahl et al. 2013	Efflux
12	ABK1_3039	ACICU_02988	ABAYE0747	A1S_2736	AdeJ - efflux cluster 3	Sahl et al. 2013	Efflux
13	ABTJ_00008	ACICU_03668	ABAYE0008	A1S_0008	adeT.1-RND family efflux pump	Huang et al. 2012	Efflux
14	ABTJ_00009	ACICU_03667	ABAYE0009	A1S_0009	adeT.2-RND family efflux pump	Huang et al. 2012	Efflux
15	ABTJ_00010	ACICU_03666	ABAYE0010	A1S_0010	adeT.3-RND family efflux pump	Huang et al. 2012	Efflux
16	ABTJ_00064	ACICU_03616	ABAYE0061	A1S_3420	abeM.2-MATE family efflux pump	Huang et al. 2012	Efflux
17	ABTJ_00108	ACICU_03570	ABAYE0110	A1S_3371	abeM.3-MATE family efflux pump	Huang et al. 2012	Efflux
18	ABTJ_00399	ACICU_03292	ABAYE0396	A1S_3092	adeT.4-RND family efflux pump	Huang et al. 2012	Efflux
19	ABTJ_00612	ACICU_03093	ABAYE0636	A1S_2844	qacEdelta1 .1-SMR family efflux pump	Huang et al. 2012	Efflux
20	ABTJ_01221	ACICU_02497	ABAYE1181	A1S_2298	qacEdelta1 .2-SMR family efflux pump	Huang et al. 2012	Efflux
21	ABTJ_01876	ACICU_01828		A1S_1755	adeT.5-RND family efflux pump	Huang et al. 2012	Efflux
22	ABTJ_03383	ACICU_00404	ABAYE3381	A1S_0395	abeM.1-MATE family efflux pump	Huang et al. 2012	Efflux
23	ABTJ_03564		ABAYE3597		tetA(B)-MFS family efflux pump	Huang et al. 2012	Efflux
24	ABTJ_00275	ACICU_03413	ABAYE0270	A1S_3218	czcB/A.2-cobalt–zinc–cadmium resistance	Huang et al. 2012	Heavy metals
25	ABTJ_00276	ACICU_03412	ABAYE0271	A1S_3217	czcB/A.1-cobalt–zinc–cadmium resistance	Huang et al. 2012	Heavy metals
26	ABTJ_02215	ACICU_01494	ABAYE2198	A1S_1454	acr3-arsenical resistance protein	Huang et al. 2012	Heavy metals
27	ABTJ_03277	ACICU_00507	ABAYE3267	A1S_0498	czcD.2-cobalt–zinc–cadmium resistance	Huang et al. 2012	Heavy metals
28	ABTJ_03832	ACICU_00012	ABAYE3897	A1S_2977	czcD.1-cobalt–zinc–cadmium resistance	Huang et al. 2012	Heavy metals
29	ABAYE1319	ACICU_02420	ABAYE1319	A1S_2218	CsuA/B - type I pili cluster 1	Eijkelkamp et al. 2011	Type I pili
30	ABAYE1320	ACICU_02418	ABAYE1320	A1S_2217	CsuA - type I pili cluster 1	Eijkelkamp et al. 2011	Type I pili
31	ABAYE1321	ACICU_02417	ABAYE1321	A1S_2216	CsuB - type I pili cluster 1	Eijkelkamp et al. 2011	Type I pili
32	ABAYE1322	ACICU_02416	ABAYE1322	A1S_2215	CsuC - type I pili cluster 1 - chaperone	Eijkelkamp et al. 2011	Type I pili
33	ABAYE1323	ACICU_02415	ABAYE1323	A1S_2214	CsuD - type I pili cluster 1 - usher	Eijkelkamp et al. 2011	Type I pili
34	ABAYE1324	ACICU_02414	ABAYE1324	A1S_2213	CsuE - type I pili cluster 1 - tip adhesion	Eijkelkamp et al. 2011	Type I pili
35	A1S_1507	ACICU_01548	ABAYE2138	A1S_1507	Type I pili cluster 2.1	Eijkelkamp et al. 2011	Type I pili
36	A1S_1508	ACICU_01549	ABAYE2137	A1S_1508	Type I pili cluster 2.2	Eijkelkamp et al. 2011	Type I pili
37	A1S_1509	ACICU_01550	ABAYE2133	A1S_1509	Type I pili cluster 2.3	Eijkelkamp et al. 2011	Type I pili
38	A1S_1510	ACICU_01551	ABAYE2132	A1S_1510	Type I pili cluster 2.4	Eijkelkamp et al. 2011	Type I pili
39	A1S_2088		ABAYE1473	A1S_2088	Type I pili cluster 3.1	Eijkelkamp et al. 2011	Type I pili
40	A1S_2089		ABAYE1472	A1S_2089	Type I pili cluster 3.2	Eijkelkamp et al. 2011	Type I pili
41	A1S_2090		ABAYE1471	A1S_2090	Type I pili cluster 3.3	Eijkelkamp et al. 2011	Type I pili
42	A1S_2091		ABAYE1470	A1S_2091	Type I pili cluster 3.4	Eijkelkamp et al. 2011	Type I pili
43	A1S_0327	ACICU_00343	ABAYE3446	A1S_0327	pilD - type IV pili cluster 1	Eijkelkamp et al. 2011	Type IV pili
44	A1S_0328	ACICU_00344	ABAYE3445	A1S_0328	pilC - type IV pili cluster 1	Eijkelkamp et al. 2011	Type IV pili
45	A1S_0329	ACICU_00345	ABAYE3444	A1S_0329	pilB - type IV pili cluster 1	Eijkelkamp et al. 2011	Type IV pili
46	A1S_0896	ACICU_00847	ABAYE2919	A1S_0896	pilU - type IV pili cluster 2	Eijkelkamp et al. 2011	Type IV pili

Row	Locus Tag	ACICU gene ID	AYE gene ID	ATCC gene ID	Annotation	References	Gene Type
**47**	A1S_0897	ACICU_00848	ABAYE2918	A1S_0897	pilT - type IV pili cluster 2	Eijkelkamp et al. 2011	Type IV pili
48	A1S_3191	ACICU_03390	ABAYE0294	A1S_3191	comQ - type IV pili cluster 3	Eijkelkamp et al. 2011	Type IV pili
49	A1S_3192	ACICU_03391	ABAYE0293	A1S_3192	comL - type IV pili cluster 3	Eijkelkamp et al. 2011	Type IV pili
50	A1S_3193	ACICU_03392	ABAYE0292	A1S_3193	comO - type IV pili cluster 3	Eijkelkamp et al. 2011	Type IV pili
51	A1S_3194	ACICU_03393	ABAYE0291	A1S_3194	comN - type IV pili cluster 3	Eijkelkamp et al. 2011	Type IV pili
52	A1S_3195	ACICU_03394	ABAYE0290	A1S_3195	comM - type IV pili cluster 3	Eijkelkamp et al. 2011	Type IV pili
53	A1S_1559	ACICU_01606	ABAYE2074	A1S_1559	pilZ - type IV pili cluster 4	Eijkelkamp et al. 2011	Type IV pili
54	A1S_2811	ACICU_03059	ABAYE0671	A1S_2811	chpA-like - type IV pili cluster 5	Eijkelkamp et al. 2011	Type IV pili
55	A1S_2812	ACICU_03060	ABAYE0670	A1S_2812	pilJ - type IV pili cluster 5	Eijkelkamp et al. 2011	Type IV pili
56	A1S_2813	ACICU_03061	ABAYE0669	A1S_2813	pilI - type IV pili cluster 5	Eijkelkamp et al. 2011	Type IV pili
57	A1S_2814	ACICU_03062	ABAYE0668	A1S_2814	pilH - type IV pili cluster 5	Eijkelkamp et al. 2011	Type IV pili
58	A1S_2815	ACICU_03063	ABAYE0667	A1S_2815	pilG - type IV pili cluster 5	Eijkelkamp et al. 2011	Type IV pili
59	A1S_0232	ACICU_00257	ABAYE3535	A1S_0232	A1S0232 - type IV pili cluster 6	Eijkelkamp et al. 2011	Type IV pili
60	A1S_0233				A1S0233 - type IV pili cluster 6	Eijkelkamp et al. 2011	Type IV pili
61	A1S_0234				pilR? - type IV pili cluster 6	Eijkelkamp et al. 2011	Type IV pili
62	A1S_0235	ACICU_00258	ABAYE3534	A1S_0235	pilS? - type IV pili cluster 6	Eijkelkamp et al. 2011	Type IV pili
63	A1S_1063	ACICU_01101	ABAYE2648	A1S_1063	biopolymer transport protein ExbD/TolR	Di Nocera et al. 2011	hemin utilization
64	A1S_1065	ACICU_01102	ABAYE2647	A1S_1065	biopolymer transport protein	Di Nocera et al. 2011	hemin utilization
65	ABAYE2040	ACICU_01639	ABAYE2040	A1S_1613	GsiA-glutathione import ATP-binding protein	Di Nocera et al. 2011	hemin utilization
66	ABAYE2041	ACICU_01638	ABAYE2041	A1S_1612	ABC-type dipeptide/oligopeptide/nickel transport system permease component	Di Nocera et al. 2011	hemin utilization
67	ABAYE2042	ACICU_01637	ABAYE2042	A1S_1611	ABC-type dipeptide/oligopeptide/nickel transport system permease component	Di Nocera et al. 2011	hemin utilization
68	ABAYE2043	ACICU_01636	ABAYE2043	A1S_1610	Zn-dependent oligopeptidase	Di Nocera et al. 2011	hemin utilization
69	ABAYE2044	ACICU_01635	ABAYE2044	A1S_1609	heme-binding protein A	Di Nocera et al. 2011	hemin utilization
70	ABAYE2045	ACICU_01634	ABAYE2045	A1S_1608	heme-binding protein A	Di Nocera et al. 2011	hemin utilization
71	ABAYE2046	ACICU_01633	ABAYE2046	A1S_1607	TonB-dependent receptor	Di Nocera et al. 2011	hemin utilization
72	A1S_1647	ACICU_01672	ABAYE2008	A1S_1647	siderophore cluster 1.1 - siderophore biosynthesis protein	Eijkelkamp et al. 2011	Iron scavenging
73	A1S_1648	ACICU_01673	ABAYE2007	A1S_1648	siderophore cluster 1.2 - lysine/ornithine N-monooxygenase	Eijkelkamp et al. 2011	Iron scavenging
74	A1S_1649	ACICU_01674	ABAYE2006	A1S_1649	siderophore cluster 1.3 - MFS superfamily MDR protein	Eijkelkamp et al. 2011	Iron scavenging
75	A1S_1650				siderophore cluster 1.4	Eijkelkamp et al. 2011	Iron scavenging
76	A1S_1651	ACICU_01675	ABAYE2005	A1S_1651	siderophore cluster 1.5	Eijkelkamp et al. 2011	Iron scavenging
77	A1S_1652	ACICU_01676	ABAYE2004	A1S_1652	siderophore cluster 1.6 - siderophore biosynthesis protein	Eijkelkamp et al. 2011	Iron scavenging
78	A1S_1653	ACICU_01677	ABAYE2003	A1S_1653	siderophore cluster 1.7	Eijkelkamp et al. 2011	Iron scavenging
79	A1S_1654	ACICU_01678	ABAYE2002	A1S_1654	siderophore cluster 1.8 - demethylmenaquinone methyltransferase	Eijkelkamp et al. 2011	Iron scavenging
80	A1S_1655	ACICU_01679	ABAYE2001	A1S_1655	siderophore cluster 1.9 - ferric siderophore receptor protein	Eijkelkamp et al. 2011	Iron scavenging
81	A1S_1656	ACICU_01681	ABAYE1999	A1S_1656	siderophore cluster 1.10 - PepSY-associated transmembrane helix family protein	Eijkelkamp et al. 2011	Iron scavenging
82	A1S_1657	ACICU_01683	ABAYE1997	A1S_1657	siderophore cluster 1.11	Eijkelkamp et al. 2011	Iron scavenging
83	A1S_2562			A1S_2562	siderophore cluster 2.1 - putative MATE efflux pumps	Eijkelkamp et al. 2011	Iron scavenging
84	A1S_2563			A1S_2563	siderophore cluster 2.2 - recognition and uptake of the ferric siderophore	Eijkelkamp et al. 2011	Iron scavenging
85	A1S_2564				siderophore cluster 2.3 - recognition and uptake of the ferric siderophore	Eijkelkamp et al. 2011	Iron scavenging
86	A1S_2565			A1S_2565	siderophore cluster 2.4 - putative efflux pumps	Eijkelkamp et al. 2011	Iron scavenging
87	A1S_2566			A1S_2566	siderophore cluster 2.5 - recognition and uptake of the ferric siderophore	Eijkelkamp et al. 2011	Iron scavenging
88	A1S_2567			A1S_2567	siderophore cluster 2.6	Eijkelkamp et al. 2011	Iron scavenging
89	A1S_2568			A1S_2568	siderophore cluster 2.7	Eijkelkamp et al. 2011	Iron scavenging
90	A1S_2569				siderophore cluster 2.8	Eijkelkamp et al. 2011	Iron scavenging
91	A1S_2570			A1S_2570	siderophore cluster 2.9	Eijkelkamp et al. 2011	Iron scavenging
92	A1S_2571			A1S_2571	siderophore cluster 2.10	Eijkelkamp et al. 2011	Iron scavenging
93	A1S_2572			A1S_2572	siderophore cluster 2.11	Eijkelkamp et al. 2011	Iron scavenging

Row	Locus Tag	ACICU gene ID	AYE gene ID	ATCC gene ID	Annotation	References	Gene Type
94	A1S_2573				siderophore cluster 2.12	Eijkelkamp et al. 2011	Iron scavenging
95	A1S_2574			A1S_2574	siderophore cluster 2.13-enterobactin synthase subunit E	Eijkelkamp et al. 2011	Iron scavenging
96	A1S_2575			A1S_2575	siderophore cluster 2.14	Eijkelkamp et al. 2011	Iron scavenging
97	A1S_2576				siderophore cluster 2.15	Eijkelkamp et al. 2011	Iron scavenging
98	A1S_2577			A1S_2577	siderophore cluster 2.16	Eijkelkamp et al. 2011	Iron scavenging
99	A1S_2578			A1S_2578	siderophore cluster 2.17	Eijkelkamp et al. 2011	Iron scavenging
100	A1S_2579			A1S_2579	siderophore cluster 2.18-2,3-dihydro-2,3-dihydroxybenzoate dehydrogenase	Eijkelkamp et al. 2011	Iron scavenging
101	A1S_2580			A1S_2580	siderophore cluster 2.19-bifunctional isochorismate lyase / aryl carrier protein	Eijkelkamp et al. 2011	Iron scavenging
102	A1S_2581			A1S_2581	siderophore cluster 2.20-isochorismate synthase	Eijkelkamp et al. 2011	Iron scavenging
103	A1S_2372	ACICU_02570	ABAYE1104	A1S_2372	siderophore cluster 3.1 - acinetobactin-isochorismate synthase	Eijkelkamp et al. 2011	Iron scavenging
104	A1S_2373	ACICU_02571	ABAYE1103	A1S_2373	siderophore cluster 3.2 - acinetobactin - BasI	Eijkelkamp et al. 2011	Iron scavenging
105	A1S_2374	ACICU_02572	ABAYE1102	A1S_2374	siderophore cluster 3.3 - acinetobactin - thioesterase	Eijkelkamp et al. 2011	Iron scavenging
106	A1S_2375	ACICU_02573	ABAYE1101	A1S_2375	siderophore cluster 3.4 - acinetobactin	Eijkelkamp et al. 2011	Iron scavenging
107	A1S_2376				siderophore cluster 3.5 - acinetobactin	Eijkelkamp et al. 2011	Iron scavenging
108	A1S_2377				siderophore cluster 3.6 - acinetobactin	Eijkelkamp et al. 2011	Iron scavenging
109	A1S_2378	ACICU_02574	ABAYE1100	A1S_2378	siderophore cluster 3.7 - acinetobactin	Eijkelkamp et al. 2011	Iron scavenging
110	A1S_2379	ACICU_02575			siderophore cluster 3.8 - acinetobactin	Eijkelkamp et al. 2011	Iron scavenging
111	A1S_2380	ACICU_02576	ABAYE1098	A1S_2379	siderophore cluster 3.9 - acinetobactin-bifunctional isochorismate lyase / aryl carrier protein	Eijkelkamp et al. 2011	Iron scavenging
112	A1S_2381	ACICU_02577	ABAYE1097	A1S_2380	siderophore cluster 3.10 - acinetobactin-enterobactin synthase subunit E	Eijkelkamp et al. 2011	Iron scavenging
113	A1S_2382	ACICU_02578	ABAYE1096	A1S_2381	siderophore cluster 3.11 - acinetobactin - BasE	Eijkelkamp et al. 2011	Iron scavenging
114	A1S_2383	ACICU_02579	ABAYE1095	A1S_2383	siderophore cluster 3.12 - acinetobactin - BasD	Eijkelkamp et al. 2011	Iron scavenging
115	A1S_2384	ACICU_02580	ABAYE1094	A1S_2384	siderophore cluster 3.13 - acinetobactin - BasC-acinetobactin siderophore biosynthesis protein	Eijkelkamp et al. 2011	Iron scavenging
116	A1S_2385	ACICU_02581	ABAYE1093	A1S_2385	siderophore cluster 3.14 - acinetobactin - BauA-ferric acinetobactin receptor	Eijkelkamp et al. 2011	Iron scavenging
117	A1S_2386	ACICU_02582	ABAYE1092	A1S_2386	siderophore cluster 3.15 - acinetobactin - BauB-ferric acinetobactin binding protein	Eijkelkamp et al. 2011	Iron scavenging
118	A1S_2387	ACICU_02583	ABAYE1091	A1S_2387	siderophore cluster 3.16 - acinetobactin - BauE-ferric acinetobactin transport system ATP-binding protein	Eijkelkamp et al. 2011	Iron scavenging
119	A1S_2388	ACICU_02584	ABAYE1090	A1S_2388	siderophore cluster 3.17 - acinetobactin - BauC-ferric acinetobactin transport system permease	Eijkelkamp et al. 2011	Iron scavenging
120	A1S_2389	ACICU_02585	ABAYE1089	A1S_2389	siderophore cluster 3.18 - acinetobactin - BauD-ferric acinetobactin transport system permease	Eijkelkamp et al. 2011	Iron scavenging
121	A1S_2390	ACICU_02587	ABAYE1087	A1S_2390	siderophore cluster 3.19 - acinetobactin - BasB-non-ribosomal peptide synthase	Eijkelkamp et al. 2011	Iron scavenging
122	A1S_2391	ACICU_02588	ABAYE1086	A1S_2391	siderophore cluster 3.20 - acinetobactin - BasA-acinetobactin biosynthesis protein	Eijkelkamp et al. 2011	Iron scavenging
123	A1S_2392	ACICU_02589	ABAYE1085	A1S_2392	siderophore cluster 3.21 - acinetobactin - BauF-acinetobactin utilization protein	Eijkelkamp et al. 2011	Iron scavenging
124	AAN28929.1				siderophore cluster 4.10 - putative siderophore receptor OM73	Eijkelkamp et al. 2011; AAN28929.1	Iron scavenging
125	AAN28930.1				siderophore cluster 4.9 - putative RND family efflux protein P114	Eijkelkamp et al. 2011; AAN28930.1	Iron scavenging
126	AAN28931.1				siderophore cluster 4.8 - isochorismatase DhbB	Eijkelkamp et al. 2011; AAN28931.1	Iron scavenging
127	AAN28932.1				siderophore cluster 4.7 - 2,3-dihydroxybenzoate-AMP ligase DhbE	Eijkelkamp et al. 2011; AAN28932.1	Iron scavenging
128	AAN28933.1				siderophore cluster 4.6 - isochorismate synthase DhbC	Eijkelkamp et al. 2011; AAN28933.1	Iron scavenging
129	AAN28934.1				siderophore cluster 4.5 - 2,3-dihydro-2,3-dihydroxybenzoate dehydrogenase DhbA	Eijkelkamp et al. 2011; AAN28934.1	Iron scavenging
130	AAN28935.1				siderophore cluster 4.4 - putative ferric enterobactin esterase Fes-like protein	Eijkelkamp et al. 2011; AAN28935.1	Iron scavenging
131	AAN28936.1				siderophore cluster 4.3 - catechol siderophore synthase DhbF-like protein	Eijkelkamp et al. 2011; AAN28936.1	Iron scavenging
132	AAN28937.1				siderophore cluster 4.2 - putative siderophore export protein P45	Eijkelkamp et al. 2011; AAN28937.1	Iron scavenging
133	AAN28938.1				siderophore cluster 4.1 - putative phosphopantetheinyl transferase	Eijkelkamp et al. 2011; AAN28938.1	Iron scavenging
134	ABAYE1888	ACICU_01791	ABAYE1888		entB - siderophore cluster 5 - isochorismatase	Eijkelkamp et al. 2011	Iron scavenging
135	ABAYE1889	ACICU_01790	ABAYE1889		entA - siderophore cluster 5 - 2,3-dihydro-2,3-dihydroxybenzoate dehydrogenase	Eijkelkamp et al. 2011	Iron scavenging
136	ABAYE1644	ACICU_02032	ABAYE1644	A1S_1921	TonB dependent ferrisiderophore receptor protein	Sahl et al. 2013	Iron scavenging
137	A1S_0979	ACICU_00941	ABAYE2813	A1S_0979	NfuA-like	Fiester and Actis 2013	protection vs oxidative stress
138	A1S_0550			A1S_0550	tssI-vgrG.1	Weber et al. 2013	T6SS
139	A1S_1288			A1S_1288	tssI-vgrG.2	Weber et al. 2013	T6SS
140	A1S_1289				tssI-vgrG.3	Weber et al. 2013	T6SS

Row	Locus Tag	ACICU gene ID	AYE gene ID	ATCC gene ID	Annotation	References	Gene Type
141	A1S_1293				tssB.1-T6SS.1	Weber et al. 2013	T6SS
142	A1S_1294	ACICU_01296	ABAYE2415	A1S_1294	tssB.2-T6SS.2	Weber et al. 2013	T6SS
143	A1S_1295	ACICU_01297	ABAYE2414	A1S_1295	tssC-T6SS.3	Weber et al. 2013	T6SS
144	A1S_1296	ACICU_01298	ABAYE2413	A1S_1296	tssD-T6SS.4	Weber et al. 2013	T6SS
145	A1S_1297	ACICU_01299	ABAYE2412	A1S_1297	tssE-T6SS.5	Weber et al. 2013	T6SS
146	A1S_1298	ACICU_01300	ABAYE2411	A1S_1298	tssF.1-T6SS.6	Weber et al. 2013	T6SS
147	A1S_1299				tssF.2-T6SS.7	Weber et al. 2013	T6SS
148	A1S_1300	ACICU_01301	ABAYE2410	A1S_1300	tssG-T6SS.8	Weber et al. 2013	T6SS
149	A1S_1301	ACICU_01302	ABAYE2409	A1S_1301	T6SS.9	Weber et al. 2013	T6SS
150	A1S_1302	ACICU_01303	ABAYE2408	A1S_1302	tssM.1-T6SS.10	Weber et al. 2013	T6SS
151	A1S_1303				tssM.2-T6SS.11	Weber et al. 2013	T6SS
152	A1S_1304	ACICU_01304	ABAYE2407	A1S_1304	tagF-T6SS.12	Weber et al. 2013	T6SS
153	A1S_1305	ACICU_01305	ABAYE2406	A1S_1305	tagN-T6SS.13	Weber et al. 2013	T6SS
154	A1S_1306	ACICU_01306	ABAYE2405	A1S_1306	T6SS.14	Weber et al. 2013	T6SS
155	A1S_1307	ACICU_01307	ABAYE2404	A1S_1307	tssH-T6SS.15	Weber et al. 2013	T6SS
156	A1S_1308	ACICU_01308	ABAYE2403	A1S_1308	tssA-T6SS.16	Weber et al. 2013	T6SS
157	A1S_1309	ACICU_01309	ABAYE2402	A1S_1309	tssK-T6SS.17	Weber et al. 2013	T6SS
158	A1S_1310	ACICU_01310	ABAYE2401	A1S_1310	tssL-T6SS.18	Weber et al. 2013	T6SS
159	A1S_3364	ACICU_03563	ABAYE0118	A1S_3364	tssI-vgrG.4	Weber et al. 2013	T6SS
160	A1S_0748				BfmR-response regulator receiver domain protein		Biofilm
161	A1S_0749	ACICU_00706	ABAYE3063	A1S_0749	BfmS-two-component system sensor kinase protein		Biofilm
162					pgaA.1	Choi et al. 2009	Biofilm
163	A1S_0938	ACICU_00900	ABAYE2855	A1S_0938	pgaB.2	Choi et al. 2009	Biofilm
164	A1S_0939	ACICU_00901	ABAYE2854	A1S_0939	pgaC.2	Choi et al. 2009	Biofilm
165	A1S_0940	ACICU_00902	ABAYE2853	A1S_0940	pgaD.2	Choi et al. 2009	Biofilm
166	A1S_2160	ACICU_02362	ABAYE1396	A1S_2160	pgaC.1	Choi et al. 2009	Biofilm
167	A1S_2161	ACICU_02363	ABAYE1395	A1S_2161	pgaB.1	Choi et al. 2009	Biofilm
168	A1S_2162	ACICU_02365	ABAYE1394	A1S_2162	pgaA.1	Choi et al. 2009	Biofilm
169	A1S_3792	ACICU_02361	ABAYE1397	A1S_3792	pgaD.1	Choi et al. 2009	Biofilm
170	AB57_0932	ACICU_00835	ABAYE2931	A1S_0884	OmpA		Biofilm
171	ABAYE0459	ACICU_03228	ABAYE0459	A1S_3032	TonB2	Zimbler et al. 2013; ZP_05829294	TonB
172	ABAYE2047	ACICU_01632	ABAYE2047	A1S_3708	tonB1-TonB1	Zimbler et al. 2013; ZP_05827390	TonB
173	ABAYE2049	ACICU_01631	ABAYE2049	A1S_1605	exbB1-TonB1	Zimbler et al. 2013; ZP_05827390	TonB
174	ABAYE2050	ACICU_01630	ABAYE2050	A1S_1604	exbD1.1-TonB1	Zimbler et al. 2013; ZP_05827390	TonB
175	ABAYE2051	ACICU_01629	ABAYE2051	A1S_1603	exbD1.2-TonB1	Zimbler et al. 2013; ZP_05827390	TonB
176	ABAYE3318	ACICU_00464	ABAYE3318	A1S_0454	exbD3-TonB3	Zimbler et al. 2013; ZP_05828403	TonB
177	ABAYE3319	ACICU_00463	ABAYE3319	A1S_0453	exbB3-TonB3	Zimbler et al. 2013; ZP_05828403	TonB
178	ABAYE3320	ACICU_00462	ABAYE3320	A1S_0452	tonB3-TonB3	Zimbler et al. 2013; ZP_05828403	TonB

*References cited in this table:

Choi AH, Slamti L, Avci FY, Pier GB, Maira-Litran T. 2009. The *pgaABCD* locus of *Acinetobacter baumannii* encodes the production of poly-beta-1-6-N-acetylglucosamine, which is critical for biofilm formation.J Bacteriol 191(19): 5953-5963.

Coyne S, Courvalin P, Perichon B. 2011. Efflux-mediated antibiotic resistance in *Acinetobacter* spp. Antimicrob Agents Chemother 55(3): 947-953.

Di Nocera PP, Rocco F, Giannouli M, Triassi M, Zarrilli R. 2011. Genome organization of epidemic *Acinetobacter baumannii* strains. BMC Microbiol 11: 224.

Eijkelkamp BA, Stroeher UH, Hassan KA, Papadimitrious MS, Paulsen IT, Brown MH. 2011. Adherence and motility characteristics of clinical *Acinetobacter baumannii* isolates. FEMS Microbiol Lett 323(1): 44-51.

Fiester SE, Actis LA. 2013. Stress responses in the opportunistic pathogen *Acinetobacter baumannii*. Future Microbiol 8(3): 353-365.

Huang H, Yang ZL, Wu XM, Wang Y, Liu YJ, Luo H, Lv X, Gan YR, Song SD, Gao F. 2012. Complete genome sequence of *Acinetobacter baumannii* MDR-TJ and insights into its mechanism of antibiotic resistance. J Antimicrob Chemother 67(12): 2825-2832.

Sahl JW, Gillece JD, Schupp JM, Waddell VG, Driebe EM, Engelthaler DM, Keim P. 2013. Evolution of a pathogen: a comparative genomics analysis identifies a genetic pathway to pathogenesis in *Acinetobacter*. PLoS One 8(1): e54287.

Weber BS, Miyata ST, Iwashkiw JA, Mortensen BL, Skaar EP, Pukatzki S, Feldman MF. 2013. Genomic and functional analysis of the type VI secretion system in *Acinetobacter*. PLoS One 8(1): e55142.

Zimbler DL, Arivett BA, Beckett AC, Menke SM, Actis LA. 2013. Functional features of TonB energy transduction systems of *Acinetobacter baumannii*. Infect Immun 81(9): 3382-3394.
