# Supplementary material for: A novel method of consensus pan-chromosome assembly and large-scale comparative analysis reveal the highly flexible pan-genome of Acinetobacter baumannii
Source: Genome Biol. 2015 Jul 21;16(1):143. doi: 10.1186/s13059-015-0701-6 (PMC4507327; doi:10.1186/s13059-015-0701-6)
Supplement: Additional file 20: Table S8. — Diversity of type I pili gene clusters in isolates analyzed. [file 13059_2015_701_MOESM20_ESM.pdf]

Table S8. Diversity of type I pili gene clusters in isolates analyzed

Isolates	Genome category	Source	Year	ST	Allele summary	Country	Type I pili clusters		
							1 (Csu)	2	3
AYE	Global	Urinary	2001	1	1-1-1-1-5-1-1	France	+	+	+
ACICU	Global	Internal	2005	2	2-2-2-2-2-2-2	Italy	+	+	-
SDF	Global	Misc.	2000	17	3-29-30-1-9-1-4	France	-	-	-
NIPH 335	Global	Respiratory	1994	10	1-3-2-1-4-4-4	Czech Republic	-	+	+
OIFC098	WRAIR	Misc.	2003	10	1-3-2-1-4-4-4	Germany	-	+	+
MDR-ZJ06	Global	Blood	2006	2	2-2-2-2-2-2-2	China	-	+	-
UH0207	US Hospital	Respiratory	2007	2	2-2-2-2-2-2-2	USA	-	+	-
UH3807	US Hospital	Respiratory	2007	2	2-2-2-2-2-2-2	USA	-	+	-
UH6207	US Hospital	Respiratory	2007	2	2-2-2-2-2-2-2	USA	-	+	-
UH8807	US Hospital	Respiratory	2007	2	2-2-2-2-2-2-2	USA	-	+	-
UH8907	US Hospital	Blood	2007	524	2-97-2-2-2-2-2	USA	-	+	-
UH11608	US Hospital	Urinary	2008	2	2-2-2-2-2-2-2	USA	-	+	-
UH13908	US Hospital	Respiratory	2008	2	2-2-2-2-2-2-2	USA	-	+	-
UH18608	US Hospital	Respiratory	2008	2	2-2-2-2-2-2-2	USA	-	+	-
UH20108	US Hospital	Urinary	2008	2	2-2-2-2-2-2-2	USA	-	+	-
NIPH 60	Global	Respiratory	1992	34	8-1-14-3-12-1-13	Czech Republic	+	-	+
NIPH 528	Global	Unknown	1982	2	2-2-2-2-2-2-2	Netherlands	+	+	-
NIPH 24	Global	Urinary	1991	2	2-2-2-2-2-2-2	Czech Republic	+	+	-
WM99c	Global	Misc.	1999	2	2-2-2-2-2-2-2	Australia	+	+	-
OIFC180	WRAIR	Misc.	2003	2	2-2-2-2-2-2-2	USA	+	+	-
OIFC338	WRAIR	Misc.	2003	2	2-2-2-2-2-2-2	USA	+	+	-
NIPH 2061	Global	Unknown	2003	2	2-2-2-2-2-2-2	Czech Republic	+	+	-
OIFC189	WRAIR	Wound	2003	2	2-2-2-2-2-2-2	USA	+	+	-
MRSN 7341	MRSN	Respiratory	2004	2	2-2-2-2-2-2-2	USA	+	+	-
AB210	Global	Misc.	2005	2	2-2-2-2-2-2-2	England	+	+	-
AB_TG27331	Global	Respiratory	2005	2	2-2-2-2-2-2-2	USA	+	+	-
AB1H8	Global	Respiratory	2005	2	2-2-2-2-2-2-2	Hong Kong	+	+	-
AB_TG27323	Global	Unknown	2005	2	2-2-2-2-2-2-2	USA	+	+	-
AB_TG27335	Global	Unknown	2005	2	2-2-2-2-2-2-2	USA	+	+	-
AB_TG27327	Global	Wound	2005	2	2-2-2-2-2-2-2	USA	+	+	-
AB_1766_8	Global	Blood	2006	2	2-2-2-2-2-2-2	USA	+	+	-
AB_TG2022	Global	Blood	2006	2	2-2-2-2-2-2-2	USA	+	+	-
AB_TG2023	Global	Blood	2006	2	2-2-2-2-2-2-2	USA	+	+	-
Naval-2	WRAIR	Blood	2006	2	2-2-2-2-2-2-2	USA	+	+	-
3990	Global	Misc.	2006	2	2-2-2-2-2-2-2	Italy	+	+	-
AB_1582-8	Global	Respiratory	2006	2	2-2-2-2-2-2-2	USA	+	+	-
AB_1595-8	Global	Urinary	2006	2	2-2-2-2-2-2-2	USA	+	+	-
AB_TG2026	Global	Wound	2006	2	2-2-2-2-2-2-2	USA	+	+	-
Naval-113	WRAIR	Wound	2006	2	2-2-2-2-2-2-2	USA	+	+	-
Naval-17	WRAIR	Wound	2006	2	2-2-2-2-2-2-2	USA	+	+	-
Naval-78	WRAIR	Wound	2006	2	2-2-2-2-2-2-2	USA	+	+	-
AB_TG2631	Global	Blood	2007	2	2-2-2-2-2-2-2	USA	+	+	-
AB_TG5064	Global	Blood	2007	2	2-2-2-2-2-2-2	USA	+	+	-
UH0707	US Hospital	Blood	2007	2	2-2-2-2-2-2-2	USA	+	+	-
UH8407	US Hospital	Blood	2007	2	2-2-2-2-2-2-2	USA	+	+	-
UH9007	US Hospital	Blood	2007	2	2-2-2-2-2-2-2	USA	+	+	-
UH8707	US Hospital	Misc.	2007	2	2-2-2-2-2-2-2	USA	+	+	-
AB_908-14-7	Global	Respiratory	2007	2	2-2-2-2-2-2-2	0	+	+	-
AB_909-05	Global	Respiratory	2007	2	2-2-2-2-2-2-2	0	+	+	-
ABNIH13	Global	Respiratory	2007	2	2-2-2-2-2-2-2	USA	+	+	-
ABNIH14	Global	Respiratory	2007	2	2-2-2-2-2-2-2	USA	+	+	-
ABNIH15	Global	Respiratory	2007	2	2-2-2-2-2-2-2	USA	+	+	-
ABNIH16	Global	Respiratory	2007	2	2-2-2-2-2-2-2	USA	+	+	-
ABNIH17	Global	Respiratory	2007	2	2-2-2-2-2-2-2	USA	+	+	-
ABNIH18	Global	Respiratory	2007	2	2-2-2-2-2-2-2	USA	+	+	-
ABNIH25	Global	Respiratory	2007	2	2-2-2-2-2-2-2	USA	+	+	-
ABNIH26	Global	Respiratory	2007	2	2-2-2-2-2-2-2	USA	+	+	-
ABNIH5	Global	Respiratory	2007	2	2-2-2-2-2-2-2	USA	+	+	-
UH0807	US Hospital	Respiratory	2007	2	2-2-2-2-2-2-2	USA	+	+	-
UH10007	US Hospital	Respiratory	2007	2	2-2-2-2-2-2-2	USA	+	+	-
UH10107	US Hospital	Respiratory	2007	2	2-2-2-2-2-2-2	USA	+	+	-
UH10707	US Hospital	Respiratory	2007	2	2-2-2-2-2-2-2	USA	+	+	-
UH2107	US Hospital	Respiratory	2007	2	2-2-2-2-2-2-2	USA	+	+	-
UH2307	US Hospital	Respiratory	2007	2	2-2-2-2-2-2-2	USA	+	+	-
UH2707	US Hospital	Respiratory	2007	2	2-2-2-2-2-2-2	USA	+	+	-
UH2907	US Hospital	Respiratory	2007	2	2-2-2-2-2-2-2	USA	+	+	-
UH5307	US Hospital	Respiratory	2007	2	2-2-2-2-2-2-2	USA	+	+	-
UH6107	US Hospital	Respiratory	2007	2	2-2-2-2-2-2-2	USA	+	+	-
UH7707	US Hospital	Respiratory	2007	2	2-2-2-2-2-2-2	USA	+	+	-
UH7807	US Hospital	Respiratory	2007	2	2-2-2-2-2-2-2	USA	+	+	-
UH8107	US Hospital	Respiratory	2007	2	2-2-2-2-2-2-2	USA	+	+	-
UH9907	US Hospital	Respiratory	2007	2	2-2-2-2-2-2-2	USA	+	+	-

Key: Strain name - finished genomes (blue), pre-2000 isolates (red), sequenced in this study (italics)

Isolates	Genome category	Source	Year	ST	Allele summary	Country	Type I pili		
							1 (Csu)	2	3

6014059	Global	Skin	2007	2	2-2-2-2-2-2-2	England	+	+	-
AB_908-12	Global	Surface	2007	2	2-2-2-2-2-2-2	0	+	+	-
ABNIH2	Global	Unknown	2007	2	2-2-2-2-2-2-2	USA	+	+	-
ABNIH4	Global	Unknown	2007	2	2-2-2-2-2-2-2	USA	+	+	-
UH7007	US Hospital	Urinary	2007	2	2-2-2-2-2-2-2	USA	+	+	-
UH9707	US Hospital	Urinary	2007	2	2-2-2-2-2-2-2	USA	+	+	-
AB_909-14-7	Global	Wound	2007	2	2-2-2-2-2-2-2	0	+	+	-
UH1007	US Hospital	Wound	2007	2	2-2-2-2-2-2-2	USA	+	+	-
UH5707	US Hospital	Wound	2007	2	2-2-2-2-2-2-2	USA	+	+	-
TYTH-1	Global	Blood	2008	2	2-2-2-2-2-2-2	Taiwan	+	+	-
UH14508	US Hospital	Blood	2008	2	2-2-2-2-2-2-2	USA	+	+	-
UMB001	Global	Blood	2008	2	2-2-2-2-2-2-2	USA	+	+	-
AB_2008-15-34-7	Global	Respiratory	2008	2	2-2-2-2-2-2-2	0	+	+	-
ABNIH24	Global	Respiratory	2008	2	2-2-2-2-2-2-2	USA	+	+	-
UH12408	US Hospital	Respiratory	2008	2	2-2-2-2-2-2-2	USA	+	+	-
UH12808	US Hospital	Respiratory	2008	2	2-2-2-2-2-2-2	USA	+	+	-
UH15208	US Hospital	Respiratory	2008	2	2-2-2-2-2-2-2	USA	+	+	-
UH16108	US Hospital	Respiratory	2008	2	2-2-2-2-2-2-2	USA	+	+	-
UH19908	US Hospital	Respiratory	2008	2	2-2-2-2-2-2-2	USA	+	+	-
AB_2008-23-07-01-7	Global	Surface	2008	2	2-2-2-2-2-2-2	0	+	+	-
UH12308	US Hospital	Urinary	2008	2	2-2-2-2-2-2-2	USA	+	+	-
UH16008	US Hospital	Wound	2008	2	2-2-2-2-2-2-2	USA	+	+	-
AB5711	MRSN	Blood	2009	2	2-2-2-2-2-2-2	USA	+	+	-
W7282	Global	Internal	2009	2	2-2-2-2-2-2-2	England	+	+	-
W6976	Global	Misc.	2009	2	2-2-2-2-2-2-2	England	+	+	-
AB_2009-04-02-7	Global	Respiratory	2009	2	2-2-2-2-2-2-2	0	+	+	-
ABNIH20	Global	Respiratory	2009	2	2-2-2-2-2-2-2	USA	+	+	-
ABNIH22	Global	Respiratory	2009	2	2-2-2-2-2-2-2	USA	+	+	-
ABNIH23	Global	Respiratory	2009	2	2-2-2-2-2-2-2	USA	+	+	-
48055	Global	Respiratory	2010	2	2-2-2-2-2-2-2	Denmark	+	+	-
53264	Global	Respiratory	2010	2	2-2-2-2-2-2-2	Denmark	+	+	-
AC12	Global	Blood	2011	2	2-2-2-2-2-2-2	Malaysia	+	+	-
ZWS1122	Global	Blood	2011	2	2-2-2-2-2-2-2	China	+	+	-
ZWS1219	Global	Blood	2011	2	2-2-2-2-2-2-2	China	+	+	-
ABIsac_ColiS	Global	Respiratory	2011	2	2-2-2-2-2-2-2	France	+	+	-
AC30	Global	Respiratory	2011	2	2-2-2-2-2-2-2	Malaysia	+	+	-
1656-2	Global	Respiratory	2004-2005	2	2-2-2-2-2-2-2	Korea	+	+	-
TCDC-AB0715	Global	Misc.	2007-2009	2	2-2-2-2-2-2-2	Taiwan	+	+	-
BJAB07104	Global	Blood	5/2007-4/2008	2	2-2-2-2-2-2-2	China	+	+	-
BJAB0868	Global	Internal	5/2007-4/2008	2	2-2-2-2-2-2-2	China	+	+	-
MDR-TJ	Global	Misc.	before 2011	2	2-2-2-2-2-2-2	China	+	+	-
Ab11111	Global	Misc.	unknown	2	2-2-2-2-2-2-2	0	+	+	-
Ab44444	Global	Misc.	unknown	2	2-2-2-2-2-2-2	0	+	+	-
AB_515-8	Global	Respiratory	unknown	2	2-2-2-2-2-2-2	USA	+	+	-
NIPH 190	Global	Unknown	1993	9	3-1-5-3-6-1-3	Czech Republic	+	+	-
NIPH 615	Global	Respiratory	1994	12	3-5-7-1-7-2-6	Czech Republic	+	+	-
NIPH 146	Global	Unknown	1993	25	3-3-2-4-7-2-4	Czech Republic	+	+	-
OIFC143	WRAIR	Wound	2003	25	3-3-2-4-7-2-4	USA	+	+	-
Naval-18	WRAIR	Wound	2006	25	3-3-2-4-7-2-4	USA	+	+	-
AB_2008-15-69	Global	Unknown	2008	25	3-3-2-4-7-2-4	0	+	+	-
UMB003	Global	Wound	2008	25	3-3-2-4-7-2-4	USA	+	+	-
AB5256	MRSN	Blood	2009	25	3-3-2-4-7-2-4	USA	+	+	-
4190	Global	Misc.	2009	25	3-3-2-4-7-2-4	Italy	+	+	-
NIPH 67	Global	Respiratory	1992	35	9-3-2-2-5-4-14	Czech Republic	+	+	-
NIPH 80	Global	Blood	1993	37	3-2-2-2-7-1-2	Czech Republic	+	+	-
NIPH 1362	Global	Unknown	2000	47	2-13-2-2-2-2-2	Czech Republic	+	+	-
UH7907	US Hospital	Blood	2007	79	26-2-2-2-29-4-5	USA	+	+	-
UH6907	US Hospital	Respiratory	2007	79	26-2-2-2-29-4-5	USA	+	+	-
UH7607	US Hospital	Urinary	2007	79	26-2-2-2-29-4-5	USA	+	+	-
UH12208	US Hospital	Respiratory	2008	79	26-2-2-2-29-4-5	USA	+	+	-
UH16208	US Hospital	Respiratory	2008	79	26-2-2-2-29-4-5	USA	+	+	-
UH19608	US Hospital	Urinary	2008	79	26-2-2-2-29-4-5	USA	+	+	-
UH22908	US Hospital	Wound	2008	79	26-2-2-2-29-4-5	USA	+	+	-
AB_1650-8	Global	Internal	2006	113	3-3-3-4-7-4-4	USA	+	+	-
AB_1649-8	Global	Respiratory	2006	113	3-3-3-4-7-4-4	USA	+	+	-
AB-HKU3-08	Global	Respiratory	2008	215	27-2-7-2-2-1-2	Hong Kong	+	+	-
AB-HKU3-10	Global	Misc.	2010	215	27-2-7-2-2-1-2	Hong Kong	+	+	-
AB_TG27343	Global	Wound	2005	422	26-72-2-2-29-4-5	USA	+	+	-
Naval-82	WRAIR	Blood	2006	428	3-1-2-3-6-1-16	USA	+	+	-
AB_1583-8	Global	Unknown	2006	422	26-72-2-2-29-4-5	USA	+	+	-
AB_2007-09-110-01-7	Global	Surface	2007	405	5-3-16-4-29-1-60	0	+	+	-
ABNIH1	Global	Unknown	2007	novel	NEW-2-2-2-2-2-2	USA	+	+	-
ABNIH3	Global	Unknown	2007	415	2-2-2-2-68-2-2	USA	+	+	-
AB_2008-15-45	Global	Respiratory	2008	415	2-2-2-2-68-2-2	0	+	+	-
AB_2008-15-70	Global	Respiratory	2008	415	2-2-2-2-68-2-2	0	+	+	-
IS-143	Global	Wound	2008	414	2-2-2-2-2-37-2	Iraq	+	+	-
ABIsac_ColiR	Global	Respiratory	2011	novel	2-NEW-2-2-2-2-2	France	+	+	-

Key: Strain name - finished genomes (blue), pre-2000 isolates (red), sequenced in this study (italics)
