# Supplementary material for: A novel method of consensus pan-chromosome assembly and large-scale comparative analysis reveal the highly flexible pan-genome of Acinetobacter baumannii
Source: Genome Biol. 2015 Jul 21;16(1):143. doi: 10.1186/s13059-015-0701-6 (PMC4507327; doi:10.1186/s13059-015-0701-6)
Supplement: Additional file 23: — Command line arguments used for running NCBI. blastall , panoct.pl , paralog_matchtable.pl , and gene_order.pl . [file 13059_2015_701_MOESM23_ESM.pdf]

Commands used for the pan-genome analysis

BLASTP:

```
$ blastall -p blastp -d combined.fasta -m9 -F F -e 0.00001
```

Ortholog clustering:

```
$ panoct.pl -b results -t combined.blast -f db.list -g combined.att_file -P
combined.fasta -S Y -L 1 -M Y -H Y -V Y -N Y -F 1.33 -G y -c 0,25,50,75,100 -T
```

Merging clusters of paralogous proteins

```
$ paralog_matchtable.pl –M matchtable_0_1.txt –P paralogs.txt > matchtable_paralog.txt
```

Assembly of pan-chromosome:

```
$ gene_order.pl -W cluster_weights.txt -M 75_core_adjacency_vector.txt -m
0_core_adjacency_vector.txt -p CL -t ../db.list -T 0 -C centroids.fasta -A Core.att -a
fGI.att -I fGI_stats.txt -P > consensus.txt
```
